# An *N*-phosphinoamidinato borasilenide: a vinyl-analogous anion containing a base-stabilised B=Si double bond†

**DOI:** 10.1039/d5sc00047e

**Published:** 2025-03-10

**Authors:** Si Jia Isabel Phang, Zheng-Feng Zhang, Ming-Der Su, Cheuk-Wai So

**Affiliations:** a School of Chemistry, Chemical Engineering and Biotechnology, Nanyang Technological University Singapore 637371 Singapore CWSo@ntu.edu.sg; b Department of Applied Chemistry, National Chiayi University Chiayi 60004 Taiwan; c Department of Medicinal and Applied Chemistry, Kaohsiung Medical University Kaohsiung 80708 Taiwan

## Abstract

Borasilenes, which feature a heterodinuclear Si=B double bond, show interesting reactivities, due to two proximal reactive sites—boron and silicon—each with distinct electronic properties. However, borasilenes remain relatively rare due to the challenge of stabilising them. To achieve a stable borasilene, both the boron and silicon centers must be supported by sterically hindered ligands, which can, however, interfere with their potential applications. In this work, we report the synthesis of an *N*-phosphinoamidinato potassium borasilenide (compound 3), which is a vinyl-analogous anion containing a base-stabilised B=Si^−^ double bond. This B=Si^−^ double bond is functional and exhibits new patterns of reactivity towards CuCl(PMe_3_), [IrCl(cod)]_2_, Me_3_SiOTf, and MeOTf, leading to the formation of a transition metal π-complex, boron–silicon-containing metallacycle, neutral borasilene and borylsilane, respectively.

## Abstract 2

An *N*-phosphinoamidinato potassium borasilenide containing an anionic Si=B double bond is a versatile building block for transferring a B=Si double bond or B/Si atoms into inorganic and organic substrates.

## Graphical abstract

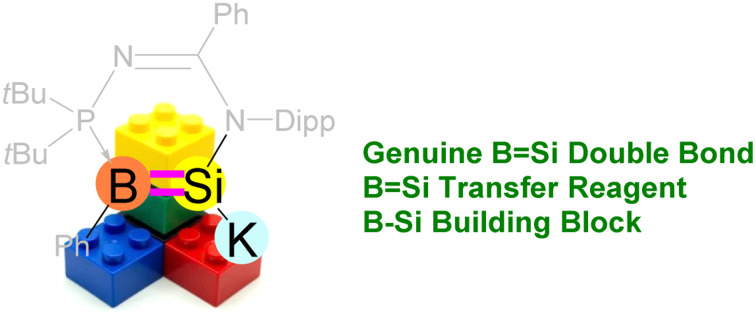

## Introduction

**Fig. 1:**
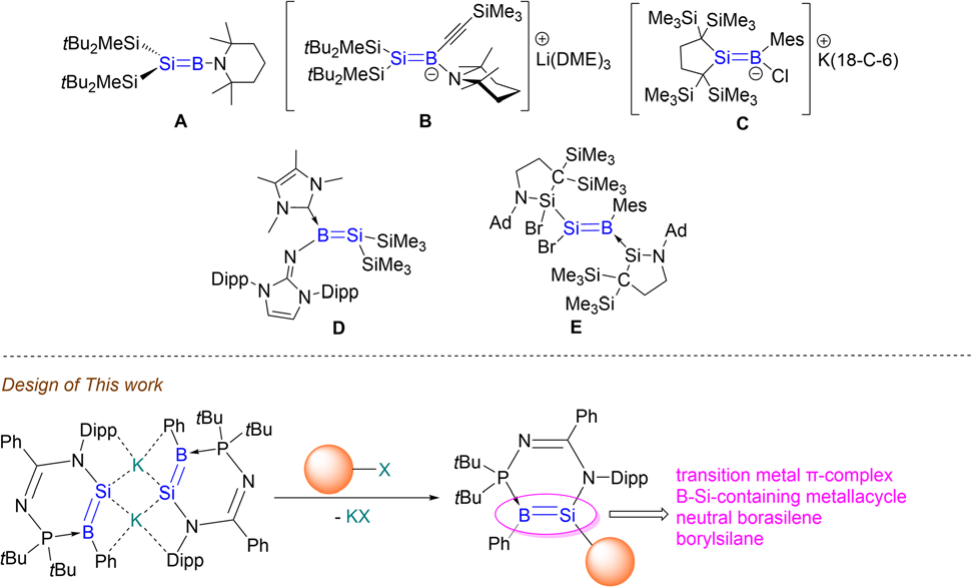
Reported examples of compounds composed of a Si=B double bond.

Emerging silicon and boron compounds have been at the center of main-group chemistry owing to their potential in small molecule activation,^1,2^ catalysis^3–9^ and materials science.^10,11^ Since the successful isolation of tetramesityldisilene^12^ in 1981 and NHC-diborene in 2007,^13^ an influx of compounds containing Si=Si double bonds, kinetically stabilised by sterically hindered ligands, have been isolated.^14–16^ Compounds possessing B=B double bonds are still in the developmental stages due to the electron deficient boron centers that require stabilisation through coordination with Lewis base ligands.^17–23^ These homodiatomic multiple bonds exhibit unprecedented reactivity patterns toward small molecules as well as intriguing photophysical properties.^24–28^ It is envisioned that borasilene, composed of a heterodinuclear B=Si double bond, should exhibit a different array of reactivities compared to their homodiatomic counterparts. This can be attributed to the two proximal reactive sites—boron and silicon—each with distinct electronic properties. Such hypothesis is evidenced by silylboronic esters or silylboranes,^29^ where the Si–B single bonds serve as versatile reactive sites for the introduction of silyl and/or boryl functionalities into organic frameworks depending on reaction conditions. As such, borasilene should be a worthwhile synthetic target. Despite their potential for synthetic applications, borasilenes are relatively rare, with only a few reported examples. To date, only one example of a stable borasilene A (Fig. 1), supported by an electron-donating amino substituent on the boron center, has been reported by Sekiguchi *et al.*^30^ Related ate and NHC complexes of borasilenes B–D have been isolated by research groups of Sekiguchi,^30^ Iwamoto^31^ and Inoue,^32^ respectively, which illustrate that the stabilisation of electron deficient boron centers is important. Their reactivity remains largely unexplored, likely due to the steric hindrance of the ligands on the B=Si double bond, which impedes reactivity. Recently, Iwamoto *et al.* reported a borasilene E containing a Br substituent on the silicon center,^33^ whereby its reactivity with anionic nucleophiles led to complex mixtures. This highlights that the use of a functional Si=B double bond in the construction of more complex Si=B double bond-containing π-conjugated systems, Si–B-heterocycles and Si–B-clusters remains a challenge.

We chose to take an alternative approach, where a borasilene derivative is made anionic, to explore its potential for metathesis reactions with a variety of main-group elements, transition metals, and organic substrates. This strategy aims to generate a more reactive Si=B double bond that can undergo functionalisation or construction of more complex boron- and silicon-containing complexes, revealing new patterns of reactivity. This approach is supported by the chemistry of disilenide anions reported by Scheschkewitz *et al.*, where they serve as versatile building blocks for synthesising novel classes of silicon-based molecules.^34,35^ Herein, we report the synthesis of an *N*-phosphinoamidinate-stabilised potassium borasilenide (compound 3), which is a vinyl anion analogue containing a B=Si^−^ double bond. It is a versatile building block for the preparation of an (η^2^-borasilene)-transition metal π-complex, boron–silicon-containing metallacycle, neutral borasilene and borylsilane, respectively.

## Results and discussion

**Scheme 1:**
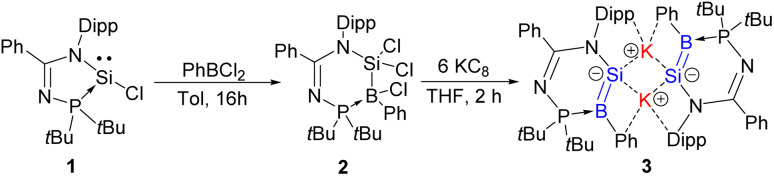
Synthesis of compounds 2 and 3.

**Fig. 2:**
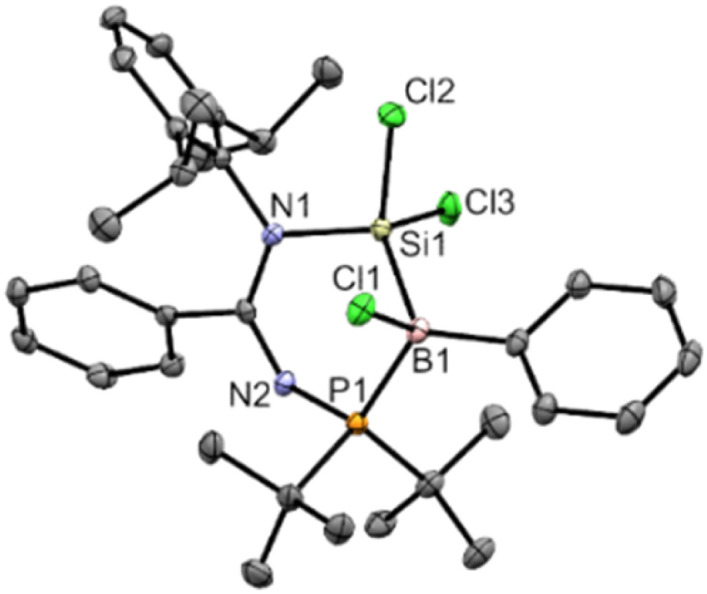
X-ray crystal structure of 2 with thermal ellipsoids at 50% probability. All H atoms are omitted for clarity. Selected bond lengths (Å) and angles (°): Cl1–B1 1.9094(19), Cl2–Si1 2.0460(6), Cl3–Si1 2.0718(6), P1–B1 2.017(2), Si1–N1 1.7806(14), Si1–B1 2.027(2), N1–Si1–B1 112.96(7), P1–B1–Si1 99.17(9), N2–P1–B1 111.59(8).

To begin with, the *N*-phosphinoamidinato chlorosilylene 1 ^36^ underwent oxidative addition with PhBCl_2_ in toluene at room temperature for 16 h to afford the *N*-phosphinoamidinate-bridged borylsilane 2 (Scheme 1), which was isolated as a colorless crystalline solid (yield: 49%) from the concentrated reaction mixture. Compound 2 was characterised by NMR spectroscopy and X-ray crystallography. The ^1^H NMR spectrum displays a set of signals corresponding to the *N*-phosphinoamidinate ligand. The ^31^P{^1^H} and ^11^B{^1^H} NMR signals are at 46.35 ppm and −5.66 ppm, respectively. The latter indicates a four-coordinate boron center. The ^29^Si{^1^H} NMR signal of 2 (3.10 ppm) is broad due to the quadrupolar coupling with the B nucleus, and it is upfield shifted in comparison with compound 1 (7.96 ppm). The molecular structure obtained by X-ray crystallography shows that the ligand is bridging between the boron and silicon centers (Fig. 2). The Si1–B1 bond length is 2.027(2) Å, which is indicative of a single bond. Similar B–Cl bond oxidative addition has been reported by Cui *et al.*^37^

Compound 2 was reacted with excess KC_8_ in THF at room temperature for 2 h to afford the dimeric *N*-phosphinoamidinato potassium borasilenide 3 (Scheme 1), which was isolated as a reddish-brown crystalline solid (yield: 42%) from its concentrated toluene solution. It is stable at room temperature in solution under an inert atmosphere.

**Fig. 3:**
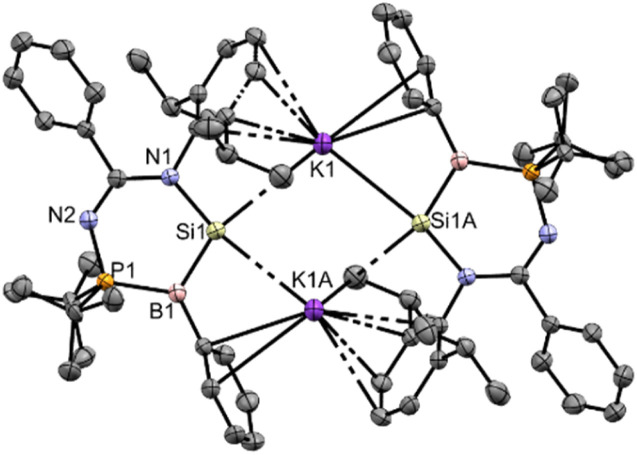
X-ray crystal structure of 3 with thermal ellipsoids at 50% probability. All H atoms are omitted for clarity. Selected bond lengths (Å) and angles (°): K1–Si1 3.3320(12), K1–Si1A 3.5479(13), P1–N2 1.648(3), P1–B1 1.878(4), Si1–N1 1.866(3), Si1–B1 1.889(4), N1–Si1–B1 106.53(15), N2–P1–B1 113.96(16), P1–B1–Si1 113.05(19), K1–Si1–K1A 78.97(3).

Compound 3 was characterised by NMR spectroscopy and X-ray crystallography. The ^31^P{^1^H} NMR signal (47.79 ppm) is broad, which is comparable with that of 2. The ^29^Si{^1^H} NMR signal (208.40 ppm) and ^11^B{^1^H} NMR signal (30.34 ppm) are downfield shifted compared to compound 6 (^29^Si{^1^H} NMR: 110.06 ppm; ^11^B{^1^H} NMR: 25.30 ppm, see below). A similar deshielding effect was found when comparing the ^29^Si{^1^H} NMR of lithium disilenide [(Tip)_2_Si=Si(Tip)Li] (Si=Si^–^: 100.5; Si=Si^–^: 94.5 ppm) to disilene [(Tip)_2_Si=Si(Tip)_2_] (53.4 ppm).^34^ Moreover, the ^29^Si{^1^H} NMR signal (208.40 ppm) is significantly downfield shifted in comparison with the ate- and silylene-complexes of borasilene (C: 58.8, E: 83 ppm),^31,33^ which comprise of planar boron and silicon centers. In addition, the ^11^B{^1^H} NMR signal (30.34 ppm) is downfield shifted in comparison with the phosphine-coordinated boron center in the *N*-phosphinoamidinato diborene (10.5 ppm)^7^ and diboravinyl cation (16.7 ppm),^17^ with a B=B double bond. The UV-Vis spectrum shows two distinct absorption bands at *λ*_max_ = 353 and 500 nm corresponding to 
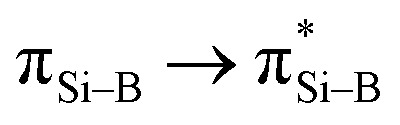
 (HOMO → LUMO+4) and 
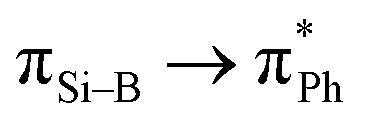
 (HOMO−1 → LUMO), respectively, based on TD-DFT calculations (M06-2X/def2-TZVP). The molecular structure of compound 3 obtained by X-ray crystallography (Fig. 3) shows that it is a contact ion pair, where the Si⋯K distance (3.3320(12), 3.5479(13) Å) is longer than the sum of their covalent radii (Si: 1.11 Å; K: 2.03 Å). The coordination sphere on the K cation is further stabilised by π-type interaction with the C atoms of Ph and Dipp substituents. The N1–Si1–B1 bond angle (106.5(1)°) is smaller than that of compound 2 (112.96(7)°), indicating the presence of a lone pair of electrons on the negatively charged silicon center. The silicon atom adopts a flattened pyramidal geometry, with the Si1, N1, B1, and K1 centers lying on the same plane, while the K1A center is positioned 1.36 Å below the plane. A similar geometry around the Si atom was found in the aluminium center of an aluminyl anion reported by Alridge *et al.*^38^ In addition, the boron atom adopts a trigonal planar geometry (sum of the bond angles: 358.8°). The Si1–B1 bond length (1.889(4) Å) is slightly longer than the Si=B double bond length in the ate- and silylene-complexes of borasilene (C: 1.859(2), E: 1.8882(16) Å),^31,33^ but is shorter than the Si–B single bond length in compound 2. All of this indicates that the Si–B bond in compound 3 has a double bond character.

**Fig. 4:**
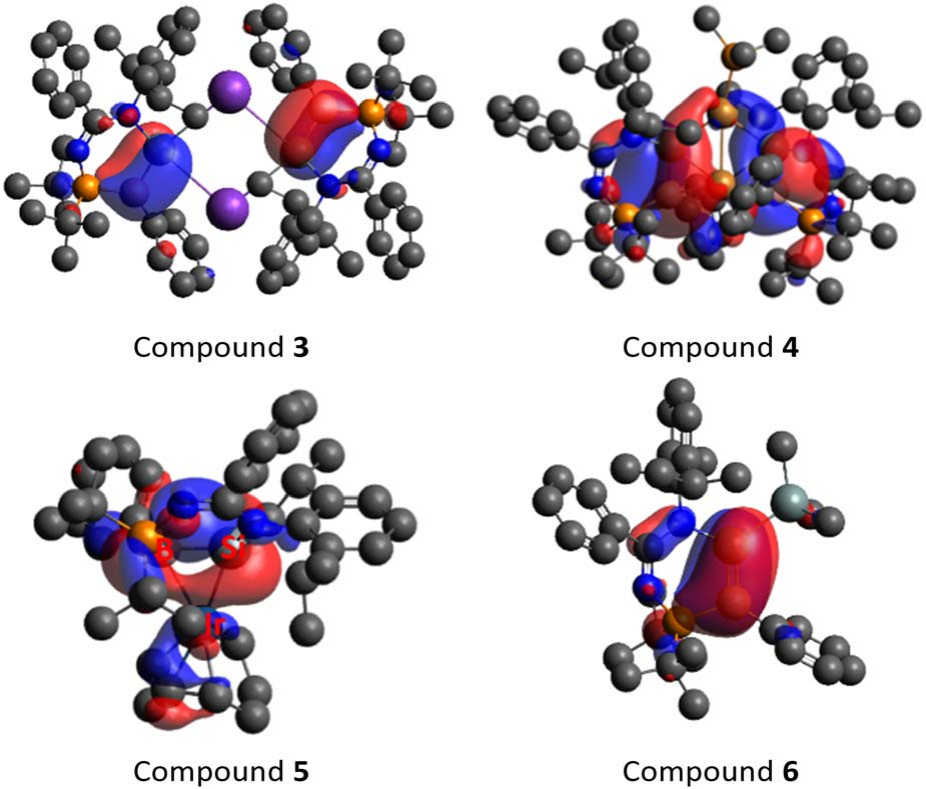
Highest occupied molecular orbitals (HOMO) of compounds 3–6 (M06-2X/def2-TZVP).

DFT calculations (M06-2X/def2-TZVP) were performed to elucidate the electronic structure of compound 3. The highest occupied molecular orbital (HOMO) corresponds to the π orbitals of the Si=B bonds (Fig. 4). The Wiberg Bond Index (WBI) of 1.586 suggests that the Si–B bond has a double bond character. Accordingly, the natural bond orbital (NBO) analysis illustrates that the Si1–B1 π bond arises from the mixing of p orbitals on the boron and silicon atoms. It is slightly polarised toward the B1 atom (56.4% B1 + 43.6% Si1). The Si1–B1 σ bond is formed by the overlapping of the sp^2.42^ hybrids on the Si1 atom and the sp^1.79^ hybrids on the B1 atom. The Si1 atom has no interaction with the K atom, but it has a σ-type lone pair of electrons (sp^0.56^, electron occupancy: 1.87 *e*). These theoretical data illustrate that compound 3 is a vinyl anion analogue containing a B=Si^−^ double bond, being consistent with the conclusion deduced from NMR spectroscopic and X-ray crystallographic data.

Despite the synthesis of disilenide and digermenide anions [R_2_E=E(R)Li] (E = Si or Ge) by Scheschkewitz *et al.*,^34,35^ the preparation of heterodinuclear vinyl anion derivatives [R_2_E′=E(R)M] (M = group 1 metal) remains challenging. Scheschkewitz *et al.* reported the only potassium silagermenide (R_2_Si=GeR′K),^39^ while Apeloig *et al.* reported the synthesis of silenide and germenide anions.^40–43^ Compound 3 is anticipated to be a highly effective reagent for transferring the Si=B double bond to various organic and inorganic substrates to yield boron–silicon-based heterocycles, clusters and complexes.

**Scheme 2:**
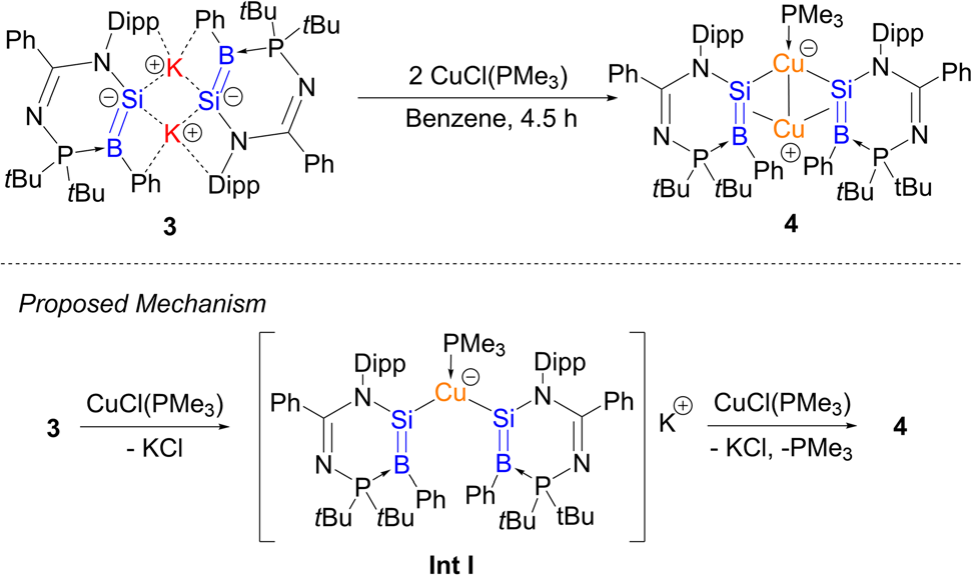
Synthesis of compound 4 and proposed mechanism for the formation of the compound.

First, the reaction of compound 3 with CuCl(PMe_3_) in benzene at room temperature for 4.5 h quantitatively afforded the (bis(borasilenyl)copper)–copper π-complex 4 (Scheme 2). In comparison with homodiatomic counterparts, an η^2^-disilene–copper complex has yet to be reported,^44^ while Braunschweig *et al.* reported some diborene–copper π-complexes.^45^ It is proposed that in the reaction, compound 3 reacts with CuCl(PMe_3_) to form a potassium bis(borasilenyl)cuprate IntI, which further undergoes metathesis reaction and π-coordination with another molecule of CuCl(PMe_3_) to form compound 4.

Compound 4 was isolated as brown crystals from the concentrated reaction mixture, which were characterised by NMR spectroscopy and X-ray crystallography. It is interesting to note that the ^29^Si{^1^H} NMR signal of 4 (234.96 ppm) is in the low-field region very similar to that of 3. The ^11^B{^1^H} NMR signal (23.80 ppm) is upfield shifted in comparison to compound 3. A similar upfield shift was observed from the bis(PMe_3_)bis(9-anthryl)diborene (22 ppm) to its CuCl complex (17.1 ppm).^45^ The UV-Vis spectrum shows one distinct absorption band at *λ*_max_ = 394 nm, which is majorly contributed by 
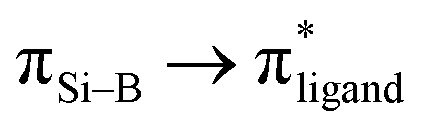
 (HOMO → LUMO+2), based on TD-DFT calculations, and a long absorption tail from 463 to 694 nm.

**Fig. 5:**
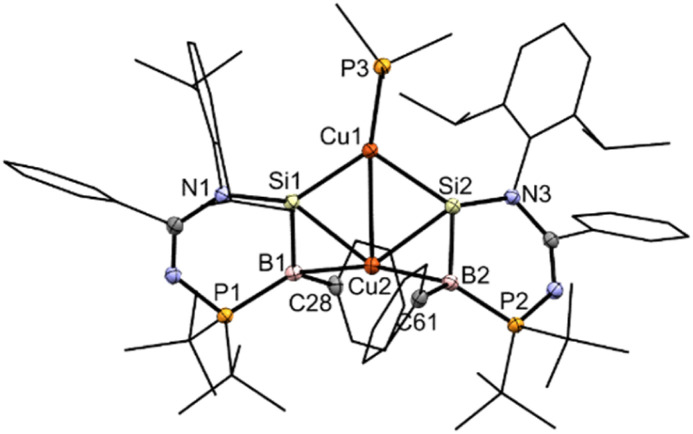
X-ray crystal structure of 4 with thermal ellipsoids at 50% probability. All H atoms are omitted for clarity. Selected bond lengths (Å) and angles (°): B1–Si1 1.921(3), B2–Si2 1.928(2), B1–Cu2 2.265(3), B2–Cu2 2.264(3), Cu1–Si1 2.3192(7), Cu1–Si2 2.3364(7), Cu2–Si2 2.3971(7), Cu2–Si1 2.4210(7), N1–Si1–B1 109.45(10), N1–Si1–Cu1 119.61(7), B1–Si1–Cu1 126.67(8), N3–Si2–B2 111.98(10), N3–Si2–Cu1 121.84(6), B2–Si2–Cu1 120.85(8), C28–B1–P1 121.61(17), C28–B1–Si1 124.45(17), P1–B1–Si1 111.01(13), C61–B2–P2 120.10(16), C61–B2–Si2 123.83(17), P2–B2–Si2 109.92(12).

The molecular structure of compound 4 obtained by X-ray crystallography (Fig. 5) shows that the Cu1 atom is bonded to the two silicon atoms of the Si=B double bonds with the Cu1–Si1/2 bond lengths (2.3192(7), 2.3364(7) Å) longer than the lithium bis(disilenyl)cuprate (2.2412(8), 2.2458(8) Å),^46^ and comparable with the potassium bis(silyl)cuprate (Ph_3_Si)_2_Cu[K(18-crown-6)THF_2_] (2.3480(7) Å).^47^ The Cu1–Si1/2 bond lengths are shorter than the sum of their covalent radii (Cu: 1.32, Si: 1.11 Å). The Si1–B1 and Si2–B2 bond lengths (1.921(3), 1.928(2) Å) are, on average, 0.036 Å longer than those in compound 3, but significantly shorter – by an average of 0.102 Å – than those in compound 2. In addition, the planarity of the Si=B double bonds is retained (sum of the bond angles, excluding Cu2, Si1: 355.73; Si2: 354.67; B1: 357.07; B2: 353.85°). The Cu2–B1/2 (2.265(3), 2.264(3) Å) and Cu2–Si1/2 (2.4210(7), 2.3971(7) Å) bond lengths are longer than that of η^2^-diborene–copper complexes (Cu–B: 2.133(3)–2.149(3) Å)^45^ and the Cu1–Si1/2 bond lengths of compound 4, respectively. All of this indicates that the Si=B π-electrons donate to the Cu2 center, while the Cu2 center with a d^10^-valence electron count exhibits a small degree of π-back bonding to the π*-MO of the Si=B double bonds in the Dewar–Chatt–Duncanson model. In this context, the Cu2 center forms a π-complex with two Si=B double bonds. The Cu1–Cu2 distance (2.7229(4) Å) is in accordance with a weak intramolecular d^10^–d^10^ interaction. DFT calculations show that the HOMO is mainly the Si=B π orbitals (Fig. 4), and the NBO analysis illustrates that the Si–B bond possesses a π orbital with a WBI of 1.52, indicating that the Si–B bond is a double bond. Based on both experimental and theoretical data, compound 4 is a bis(η^2^-borasilene)–copper π-complex.

**Scheme 3:**
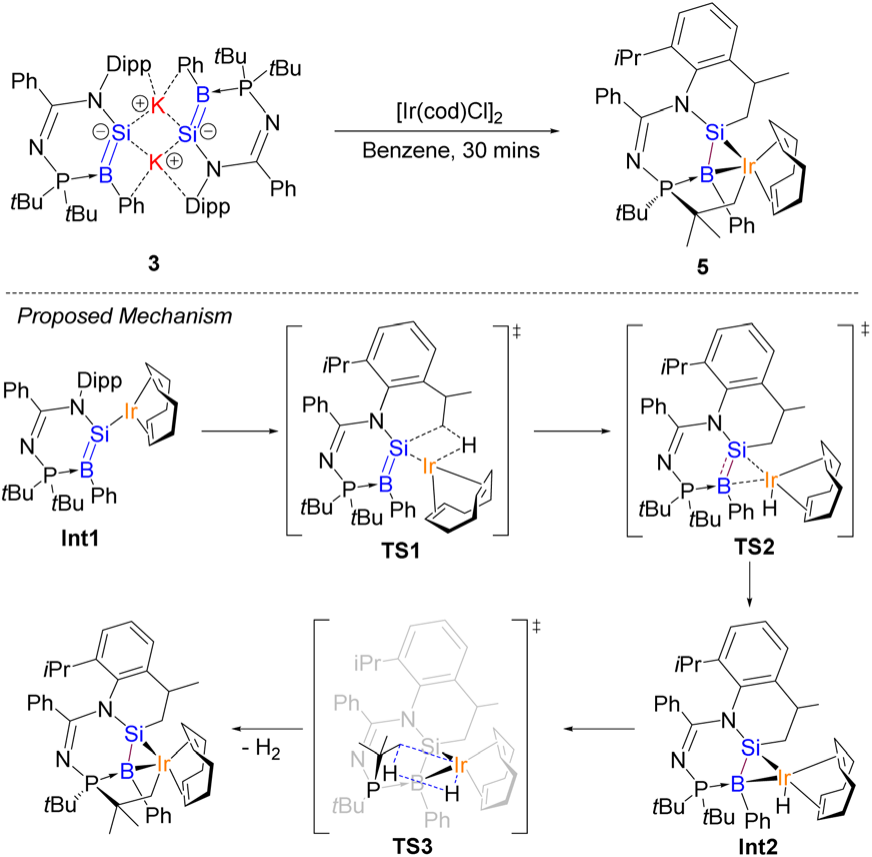
Synthesis of compound 5 and proposed mechanism for the formation of the compound.

Besides copper, compound 3 could also react with heavy transition metal electrophiles, namely iridium. As a proof-of-principle, the reaction of compound 3 with [Ir(cod)Cl]_2_ in benzene at room temperature for 30 min quantitatively affords the B–Si–Ir metallacyclic complex 5 (Scheme 3). We propose that the monomeric derivative of compound 3 first reacts with a unit of [Ir(cod)Cl]_2_ to form an iridium borasilenide intermediate, Int1. Following this, it undergoes a C–H insertion reaction with the iPr of Dipp substituent *via*TS1 to form a neutral borasilene and (cod)hydridoiridium(i) moieties, where the B=Si double bond coordinates with the iridium center *via*TS2 to afford an (η^2^-borasilene)iridium intermediate, Int2. It subsequently undergoes another C–H insertion reaction with the *t*Bu substituent *via*TS3 to form compound 5 and H_2_.

Compound 5 was isolated as orange crystals from the concentrated reaction mixture, which were characterised by NMR spectroscopy and X-ray crystallography. The ^11^B{^1^H} NMR signal (−58.93 ppm) is in the high field region similar to four-coordinate boron metal complexes such as the (CAAC)(dicyano)borylgold complex (−26.2 ppm).^48^ The ^29^Si NMR of compound 5 could not be obtained, due to the presence of quadrupolar coupling with the ^11^B and ^193^Ir nuclei. The UV-Vis spectrum shows a distinct absorption band at 350 nm corresponding to d_*z*^2^_ → p_π_ + d_*xy*_ (HOMO−3 → LUMO), based on TD-DFT calculations.

**Fig. 6:**
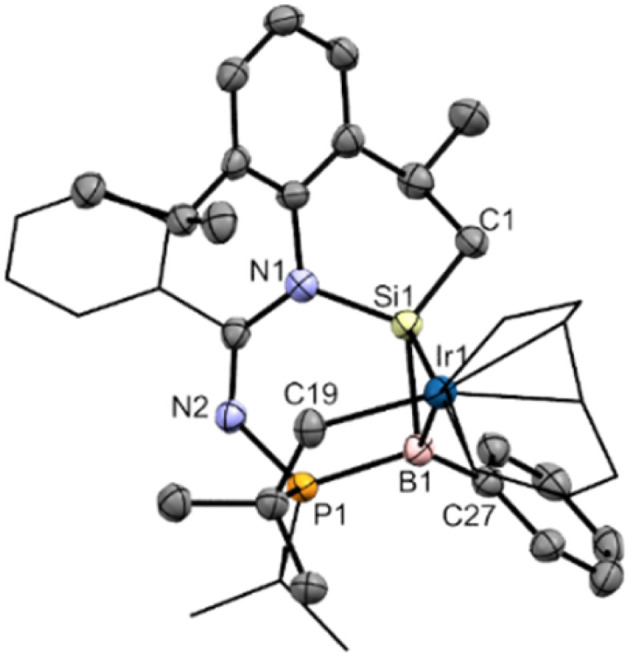
X-ray crystal structure of 5 with thermal ellipsoids at 50% probability. All H atoms are omitted for clarity. Selected bond lengths (Å) and angles (°): B1–Si1 1.989(7), B1–Ir1 2.529(6), Ir1–Si1 2.3714(16), B1–P1 1.920(7), N1–Si1 1.791(5), N2–P1 1.681(5), N1–Si1–C1 100.1(3), N1–Si1–B1 113.6(3), C1–Si1–B1 124.2(3), C27–B1–P1 123.0(4), C27–B1–Si1 118.1(4), P1–B1–Si1 102.1(3).

The molecular structure of compound 5 obtained by X-ray crystallography shows that the Si1–B1 bond (1.989(7) Å) is slightly shorter than that of compound 2 (2.027 Å) by 0.038 Å (Fig. 6). In addition, the planarity of the borasilene moiety was not retained, where both boron and silicon centers adopt a trigonal pyramidal geometry (sum of the bond angles, excluding the Ir center, Si1: 337.9; B1: 343.2°). The Ir1–B1 bond (2.529(6) Å) is longer than the Ir–B_boryl_ bond in PBP-pincer iridium complexes (1.97–2.14 Å),^49^ while the Ir1–Si1 bond (2.371(2) Å) is comparable with that of the octahedral Ir-silyl complexes^50^ (*ca.* 2.39 Å), and shorter than that of the iridium silene complex [Cp*(PMe_3_)Ir(η^2^-CH_2_SiMe_2_)(H)][B(C_6_F_5_)_4_] (2.439(8) Å).^51^ This indicates that the Ir center, with a d^8^-valence electron count, exhibits a large degree of π-back bonding to the π*-MO of the borasilene. The shorter Ir–Si bond length reflects the greater degree of covalent bonding character, therefore elucidating that compound 5 is a metallacyclopropane, with its molecular structure revealing a three-membered B–Si–Ir ring rather than the classical π-complex. DFT calculations support the conclusion as the HOMO is composed of the Si and B p_π_ orbitals overlapped with the Ir d_*x*^2^–*y*^2^_ orbital (Fig. 4), and the NBO analysis illustrates that the Si1–B1 bond possesses solely a σ orbital (Si sp^2.19^ + B sp^3.45^) with a WBI of 1.08, indicating that the Si–B bond is a single bond. In this context, compound 5 is a B–Si–Ir metallacycle. Comparing compounds 4 and 5 with other heavier alkene analogues, the latter also demonstrate intriguing coordination modes with transition metals.^44^ For example, Berry *et al.* showed that a tungsten complex with an unhindered disilene Me_2_Si=SiMe_2_ exhibits properties between those of a π-complex and metallacycle, analogous to the bonding situation observed in transition-metal complexes of olefins.^52^ Scheschkewitz *et al.* demonstrated that the Si=Ge double bond in a disilagermirene–nickel complex did not follow the Dewar–Chatt–Duncanson model, as the Si–Ge σ-electrons, rather than the π-electrons, donate to the nickel center.^53^

**Scheme 4:**
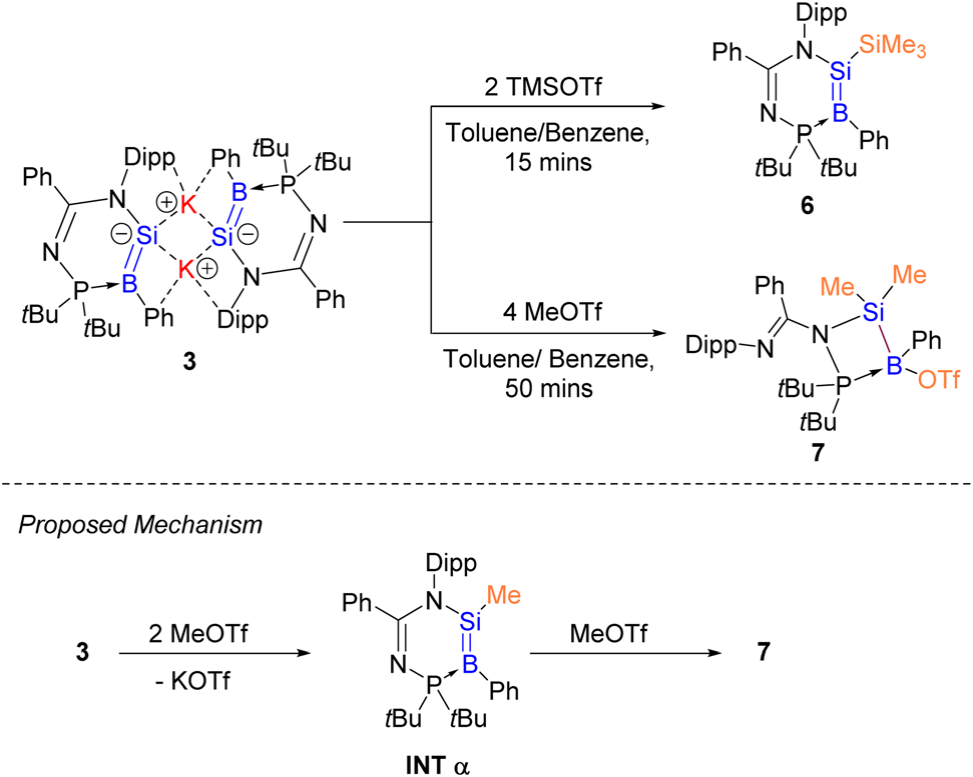
Synthesis of compounds 6 and 7 and proposed mechanism for the formation of compound 7.

To show that compound 3 can generate a free neutral borasilene, Me_3_SiOTf was reacted with compound 3 in benzene at room temperature for 15 min, resulting in the quantitative formation of compound 6 (Scheme 4). Compound 6 was isolated as orange crystals from the concentrated reaction mixture, which were characterised by NMR spectroscopy and X-ray crystallography. The ^11^B{^1^H} NMR signal (25.30 ppm) and ^29^Si{^1^H} NMR signal (110.06 ppm) are upfield shifted in comparison with that of compound 3. The UV-Vis spectrum shows three distinct absorption bands at *λ*_max_ = 279, 372, and 434 nm corresponding to 
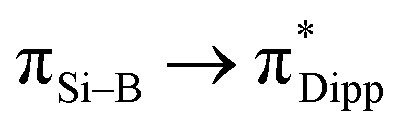
 (HOMO → LUMO+3), 
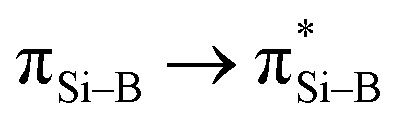
 (HOMO → LUMO+1) and 
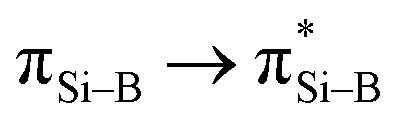
 (HOMO → LUMO), respectively, based on TD-DFT calculations.

**Fig. 7:**
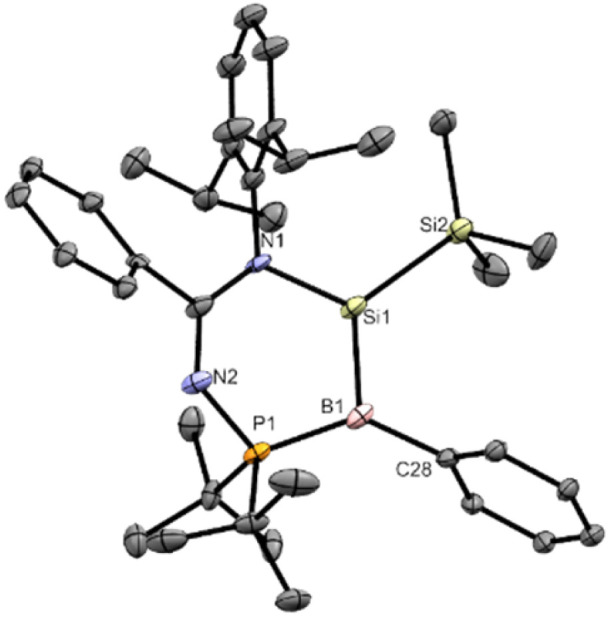
X-ray crystal structure of 6 with thermal ellipsoids at 50% probability. All H atoms are omitted for clarity. Selected bond lengths (Å) and angles (°) of 6: B1–Si1 1.865(6), B1–P1 1.880(7), N1–Si1 1.814(4), N2–P1 1.647(4), Si1–Si2 2.349(2), C28–B1–Si1 118.2(5), C28–B1–P1 134.3(5), Si1–B1–P1 107.2(3), N1–Si1–B1 116.1(3), N1–Si1–Si2 120.99(15), B1–Si1–Si2 122.8(2).

The molecular structure of compound 6 obtained by X-ray crystallography shows that the planes of the planar boron center (sum of the bond angles: 359.7°) and silicon center (359.9°) form a dihedral angle of 9.9° (Fig. 7). The B1–Si1 bond (1.865(6) Å) is comparable to that of compound 3. The electronic structure of compound 6 was elucidated by DFT calculations. The HOMO and HOMO−1 correspond to the B=Si π and σ orbitals respectively (Fig. 4 and S50†). The WBI of 1.66 suggests that the Si=B double bond has a stronger double bond character than that in compound 3. Accordingly, NBO analysis illustrates that the Si1–B1 π bond arises from the mixing of p orbitals on the boron and silicon atoms with even contribution (48.9% B1 + 51.1% Si1), whereas the Si=B double bond in compound 3 is slightly polarised. The Si1–B1 σ bond is formed by the overlapping of the sp^1.28^ hybrids on the Si1 atom and the sp^1.87^ hybrids on the B1 atom. The introduction of the SiMe_3_ functionality could open the possibility of using compound 6 as a B=Si double bond transfer reagent, based on the recent work of a Lewis base-stabilised phosphaborene with a trimethylsilyl functionality reported by Andrada *et al.*^54^

**Fig. 8:**
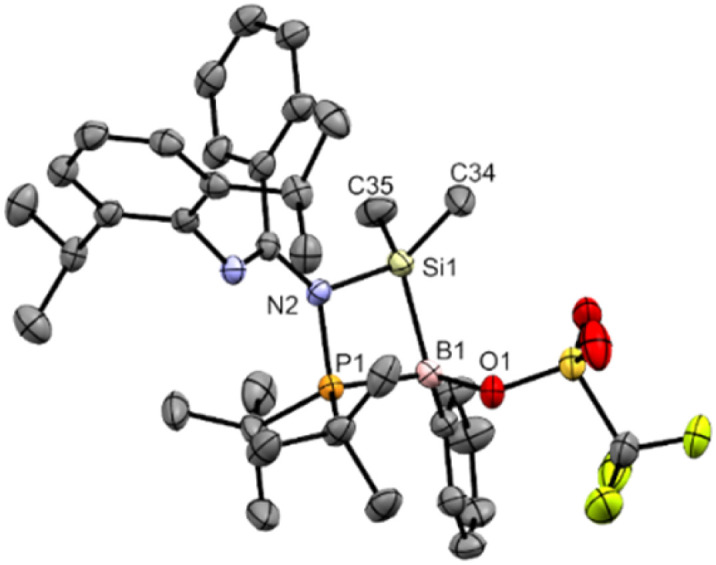
X-ray crystal structure of 7 with thermal ellipsoids at 50% probability. All H atoms are omitted for clarity. Selected bond lengths (Å) and angles (°) of 7: B1–P1 2.033(4), B1–Si1 2.036(4), N2–P1 1.714(3), N2–Si1 1.815(3), P1–Si1 2.7149(12), P1–B1–Si1 83.70(14), P1–N2–Si1 100.54(13), N2–P1–B1 89.24(14), N2–Si1–B1 86.41(13).

It is anticipated that a less sterically hindered substituent could enable the B=Si double bond to undergo further addition reaction or cycloaddition. To prove our hypothesis, compound 3 was reacted with two equivalents of MeOTf in benzene at room temperature for 50 min. We propose that MeOTf reacts with compound 3 to form Intα composed of a less sterically hindered methyl substituent, where the B=Si double bond undergoes further addition reaction with the second molecule of MeOTf to quantitatively form compound 7 (Scheme 4). It should be noted that compound 7 is prone to decompose over time in solution. The molecular structure obtained by X-ray crystallography shows that the *N*-phosphinoamidinate ligand is in a *P*,*N*-chelate fashion, bridged across the B–Si bond to form a four-membered ring. The B–Si bond length (2.036(4) Å) is comparable with that of 2, indicating that it is a single bond (Fig. 8).

## Conclusions

In conclusion, the *N*-phosphinoamidinato potassium borasilenide 3 was synthesised, which is a vinyl anion derivative containing a B=Si^−^ double bond. It is a versatile Si=B double bond transfer agent to a variety of organic and inorganic substrates, namely CuCl(PMe_3_), [IrCl(cod)]_2_, Me_3_SiOTf, and MeOTf, forming an (η^2^-borasilene)-transition metal π-complex, boron–silicon-containing metallacycle, neutral borasilene and borylsilane, respectively.

## Data availability

The data supporting this article have been included as part of the ESI.† Deposition numbers 2394597 for 2, 2394598 for 3, 2394599 for 4, 2394600 for 5, 2394601 for 6, and 2394602 for 7 contain the supplementary crystallographic data for this paper. These data are provided free of charge by the joint Cambridge Crystallographic Data Centre and Fachinformationszentrum Karlsruhe Access Structures service.

## Author contributions

S. J. I. Phang designed and performed the experimental study. Z.-F. Zhang designed and performed the computational study. M.-D. Su and C.-W. So prepared the manuscript.

## Conflicts of interest

There is no conflict of interest among the authors.

## Supplementary Material

SC-OLF-D5SC00047E-s001

SC-OLF-D5SC00047E-s002
