# Supplementary material for: An N-phosphinoamidinato borasilenide: a vinyl-analogous anion containing a base-stabilised B[double bond, length as m-dash]Si double bond
Source: Chem Sci. 2025 Mar 10;16(16):6763–9. doi: 10.1039/d5sc00047e (PMC11920901; doi:10.1039/d5sc00047e)
Supplement: SC-016-D5SC00047E-s001 [file SC-016-D5SC00047E-s001.pdf]

Supplementary Information (SI) for Chemical Science.
This journal is © The Royal Society of Chemistry 2025

# An *N*-Phosphinoamidinato Borasilenide: A Vinyl-Analogous Anion Containing a Base-Stabilised B=Si Double Bond

Si Jia Isabel Phang,[a] Zheng-Feng Zhang,[b] Ming-Der Su,*[b,c] and Cheuk-Wai So*[a]

[a]School of Chemistry, Chemical Engineering and Biotechnology, Nanyang Technological University, Singapore 637371, Singapore, Email: CWSo@ntu.edu.sg.

[b]Department of Applied Chemistry, National Chiayi University, Chiayi 60004, Taiwan, Email: midesu@mail.ncyu.edu.tw.

[c]Department of Medicinal and Applied Chemistry, Kaohsiung Medical University, Kaohsiung 80708, Taiwan.

**Table of Contents**

S1. Experimental Section

S2. Selected NMR Spectra

S3. UV-vis Spectra

S4. X-Ray Data Collection and Structural Refinement

S5. Theoretical Studies

## S1. Experimental Section

**General procedure.** All manipulations were carried out under an argon atmosphere with Schlenk techniques and glovebox. Hexane, toluene and diethyl ether were purified through a MBRAUN solvent purification system. Tetrahydrofuran and benzene were purified by distillation over potassium/benzophenone. Fluorobenzene was purified by distillation over calcium hydride. Benzene-$d_6$ and tetrahydrofuran-$d_8$ were distilled over potassium metal. Chemicals were purchased from Sigma-Aldrich and directly used without purification. Compound **1** and CuCl($PMe_3$) were synthesized according to reported procedures.[S1,S2] $^{1}$H, $^{11}$B{$^{1}$H}, $^{31}$P{$^{1}$H}, $^{13}$C{$^{1}$H}, and $^{29}$Si{$^{1}$H} NMR spectra were measured on a Bruker Avance III 400 with a Dual Resonance Probe (BBFO) or JEOL (ECA 400) spectrometer. Deuterated solvents were used for the recording of NMR spectra, and chemical shifts are given in δ (ppm) and coupling constants *J* in Hz. NMR multiplicities are abbreviated, where s = singlet, d = doublet, m = multiplet, sep = septet and br = broad signal. The solid-state $^{31}$P, $^{29}$Si and $^{11}$B NMR experiments were conducted at 11.7 T on a 500 MHz JEOL NMR spectrometer (JNM-ECZL500G) and equipped with a 3.2 mm double-resonance HXMAS probe. The $^{29}$Si and $^{11}$B solid state NMR spectroscopy were ran using Cross-Polarization Magic Angle Spinning (CPMAS) experiment at 12 kHz with reference to silicone rubber (-21.50 ppm) and $NaBH_4$ (-3.61 ppm), respectively. The $^{31}$P solid state NMR spectroscopy was ran using CPMAS at 6 kHz with reference to $NH_4H_2PO_4$ (2.14 ppm). UV-vis was ran using Shimadzu UV Spectrophotometer UV-1800. HRMS spectra were obtained at the Mass Spectrometry Laboratory in the School of Chemistry, Chemical Engineering and Biotechnology, Nanyang Technological University.

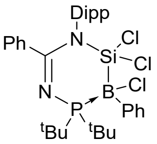


Synthesis of **2.** *N*-phosphinoamidinato chlorosilylene **1** (0.974 g, 2 mmol) and $PhBCl_2$ (0.349 g, 2.2 mmol) were dissolved in toluene in two separate 100 mL flasks. $PhBCl_2$ was added to **1** dropwise at -78 °C and the reaction mixture was allowed to warm to room temperature and stirred for 16 hours. Resulting suspension was filtered and filtrate was concentrated and stored at room temperature to yield colourless crystals**.** Yield: 0.632 g (49%). $^{1}$H NMR ($C_6D_6$, 400 MHz, 25 °C): δ 8.26 (d, 2H, Ar-H, *J* = 7.5 Hz), 7.27 (t, 2H, Ar-H, *J* = 7.5 Hz), 7.18 – 7.07 (m, 5H, Ar-H), 6.92 (dd, 1H, Ar-H, *J* = 6.8, 2.5 Hz), 6.80 (d, 3H, Ar-H, *J* = 3.0 Hz), 4.47 (sep, 1H, C*H*$Me_2$, *J* = 6.8 Hz), 3.27 (sep, 1H, C*H*$Me_2$, *J* = 6.6 Hz), 1.51 (d, 3H, CH(C*H*$_3$)$_2$, *J* = 6.6 Hz), 1.41 (d, 9H, C(C*H*$_3$)$_3$, *J* = 14.3 Hz), 1.36 (d, 3H, CH(C*H*$_3$)$_2$, *J* = 6.8 Hz), 1.33 (d, 3H, CH(C*H*$_3$)$_2$, *J* = 6.5 Hz), 1.21 (d, 9H, C(C*H*$_3$)$_3$, *J* = 14.1 Hz), 0.19 (d, 3H, CH(C*H*$_3$)$_2$, *J* = 6.6 Hz). $^{13}$C{$^{1}$H} NMR (THF-$d_8$, 101 MHz, 25 °C): δ171.93 (d, N*C*N, *J* = 9.6 Hz), 148.77 (Ar-C), 147.74 (Ar-C), 140.72 (Ar-C), 136.01 (Ar-C), 135.79 (Ar-C), 129.96 (Ar-C),129.47 (Ar-C), 129.18 (Ar-C), 127.91 (Ar-C), 127.24 (Ar-C), 125.96 (Ar-C), 125.39 (Ar-C), 41.61 (d, *C*(CH$_3$)$_3$, *J* = 30.8 Hz), 39.22 (d, *C*(CH$_3$)$_3$, *J* = 35.1 Hz), 29.52 (*C*H(CH$_3$)$_2$), 28.90 (C(*C*H$_3$)$_3$), 28.62 (C(*C*H$_3$)$_3$), 27.96 (*C*H(CH$_3$)$_2$), 27.32 (CH(*C*H$_3$)$_2$), 22.77 (CH(*C*H$_3$)$_2$). $^{31}$P{$^{1}$H} NMR ($C_6D_6$, 162 MHz, 25 °C): δ 46.35. $^{11}$B{$^{1}$H} NMR ($C_6D_6$, 128 MHz, 25 °C): δ -5.66. $^{29}$Si{$^{1}$H} ($C_6D_6$, 79 MHz, 25 °C): δ 3.10. HRMS (ESI): m/z calcd for: 645.2327 [(M + H)]$^+$; found: 645.2328.

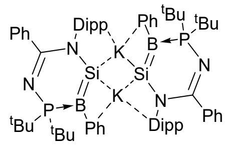


**Synthesis of 3.** THF (30 mL) was added to a 100 mL flask containing **2** (0.646 g, 1 mmol) and excess $KC_8$ (0.811 g, 6 mmol) at -78 °C. The reaction mixture was allowed to warm to room temperature and stirred for 2 hours. The resulting suspension was filtered and volatiles in the filtrate were removed. The crude solid was extracted with toluene and the solution was concentrated to yield reddish-brown crystals. Yield: 0.241 g (42%). $^{1}$H NMR ($C_6D_6$, 400 MHz, 25 °C): δ 7.82 (d, 2H, Ar-H, *J* = 7.5 Hz), 7.53 (d, 2H, Ar-H, *J* = 8.0 Hz), 7.05 – 6.91 (m, 4H, Ar-H), 6.89 – 6.55 (m, 5H, Ar-H), 3.50 (dd, 2H, C*H*$Me_2$, *J* = 13.7, 6.9 Hz), 1.45 (d, 18H,

$C(CH_3)_3$, $J$ = 13.4 Hz), 1.27 (d, 6H, $CH(CH_3)_2$, $J$ = 6.7 Hz), 0.96 (d, 6H, $CH(CH_3)_2$, $J$ = 7.1 Hz). $^{13}C\{^1H\}$ NMR ($C_7D_8$, 101 MHz, 25 °C): δ 165.70 (NCN), 148.54 (Ar-C), 146.37(Ar-C), 132.79 (Ar-C), 129.33 (Ar-C), 129.11 (Ar-C), 128.57 (Ar-C), 127.36 (Ar-C), 127.14 (Ar-C), 125.94 (Ar-C), 125.70 (Ar-C), 124.03 (Ar-C), 122.77 (Ar-C), 36.60 (d, $C(CH_3)_3$, $J$ = 40.1 Hz), 28.53 (d, $C(CH_3)_3$, $J$ = 3.6 Hz), 28.38 ($CH(CH_3)_2$), 26.08 ($CH(CH_3)_2$), 23.23 ($CH(CH_3)_2$). $^{31}P\{^1H\}$ NMR ($C_6D_6$, 162 MHz, 25 °C): δ 47.79. $^{11}B\{^1H\}$ NMR ($C_6D_6$, 128 MHz, 25 °C): δ 30.34 (m). $^{29}Si\{^1H\}$ ($C_6D_6$, 79 MHz, 25 °C): δ 208.40. HRMS (ESI): m/z calcd for: 1157.5718 [(M + H)]$^+$; found: 1157.5732.

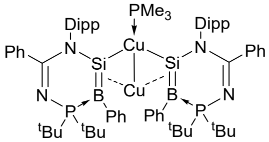


**Synthesis of 4**. Benzene (20 mL) was added to a 100 mL flask containing **3** (0.116 g, 0.1 mmol) and $CuCl(PMe_3)$ (0.0350 g, 0.2 mmol) at room temperature, and the reaction mixture was stirred for 4.5h to quantitatively form compound **4**, traced by $^1H$ and $^{31}P$ NMR spectroscopy. X-ray-crystallography-quality brown crystals (isolated yield: 0.0335 g (23%)) were afforded from the concentrated filtrate. $^1H$ NMR ($C_6D_6$, 400 MHz, 25 °C): δ 8.11 (d, 3H, Ar-H, $J$ = 7.3 Hz), 7.37 (t, 4H, Ar-H, $J$ = 7.4 Hz), 7.20 (d, 6H, Ar-H, $J$ = 7.2 Hz), 7.07 – 6.94 (m, 3H, Ar-H), 6.93 – 6.84 (m, 5H, Ar-H), 6.84 – 6.72 (m, 5H, Ar-H), 3.65 – 3.49 (m, 2H, $CHMe_2$), 3.26 – 3.10 (m, 2H, $CHMe_2$), 1.54 (d, 13H, $C(CH_3)_3$, $J$ = 13.1 Hz), 1.43 (d, 3H, $C(CH_3)_3$, $J$ = 9.3 Hz), 1.39 (d, 8H, $C(CH_3)_3$, $J$ = 13.1 Hz),1.25 (d, 12H, $C(CH_3)_3$, $J$ = 13.1 Hz), 1.20 (d, 6H, $CH(CH_3)_2$, $J$ = 7.1 Hz), 1.15 (dd, 6H, $CH(CH_3)_2$, $J$ = 9.3, 5.4 Hz), 1.13 – 1.07 (m, 3H, $P(CH_3)_3$), 0.96 (d, 6H, $CH(CH_3)_2$, $J$ = 6.7 Hz), 0.65 (br, 6H, $P(CH_3)_3$), 0.24 (d, 6H, $CH(CH_3)_2$, $J$ = 5.8 Hz).$^{13}C\{^1H\}$ NMR ($C_6D_6$, 101 MHz, 25 °C): δ 163.63 (d, NCN, $J$ = 10.3 Hz), 146.77 (Ar-C), 144.96 (Ar-C), 140.62 (Ar-C), 138.52 (Ar-C), 128.89 (Ar-C), 127.31 (Ar-C), 127.18 (Ar-C), 127.03 (Ar-C), 126.90 (Ar-C), 124.73 (Ar-C), 124.02 (Ar-C), 122.98 (Ar-C), 38.35 (d, $C(CH_3)_3$, $J$ = 37.8 Hz), 37.50 (d, $C(CH_3)_3$, $J$ = 40.7 Hz), 34.42 ($CH(CH_3)_2$), 34.28 ($CH(CH_3)_2$), 29.32 ($C(CH_3)_3$), 28.93 ($C(CH_3)_3$), 28.84 ($C(CH_3)_3$), 28.76 ($C(CH_3)_3$), 28.68 ($C(CH_3)_3$), 28.51 ($C(CH_3)_3$), 26.93($CH(CH_3)_2$), 24.75 ($CH(CH_3)_2$), 23.37 ($CH(CH_3)_2$), 22.76 ($CH(CH_3)_2$), 16.26 ($P(CH_3)_3$), 16.11 ($P(CH_3)_3$). $^{31}P\{^1H\}$ NMR ($C_6D_6$, 162 MHz, 25 °C): δ 43.14, -51.05. $^{11}B\{^1H\}$ NMR ($C_6D_6$, 128 MHz, 25 °C): δ 23.80 (br). $^{29}Si\{^1H\}$ ($C_6D_6$, 79 MHz, 25 °C): δ 234.96 (br). HRMS (ESI): m/z calcd for: 1283.5459 [(M + H)]$^+$; found: 1283.5504.

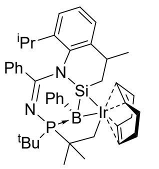


**Synthesis of 5.** Benzene (20 mL) was added to a 100 mL flask containing **3** (0.116 g, 0.1 mmol) and $[Ir(cod)Cl]_2$ (0.0671g, 0.1 mmol) at room temperature. The reaction mixture was stirred for 30 mins to quantitatively form compound **5**, traced by $^1H$ and $^{31}P$ NMR spectroscopy. X-ray-crystallography-quality orange crystals (isolated yield: 0.0268 g (16%)) were afforded from the concentrated filtrate. $^1H$ NMR (THF-$d_8$, 400 MHz, 25 °C): δ 7.80 – 7.69 (m, 2H, Ar-H), 7.29 – 7.09 (m, overlapping signals, 8H, Ar-H), 7.08 – 7.01 (m, 2H, Ar-H), 7.01 – 6.95 (m, 1H, Ar-H), 3.87 (t, 1H, $CHMe_2$, $J$ = 8.3 Hz), 3.81 – 3.70 (m, 1H, cod-H), 3.51 – 3.40 (m, 2H, cod-H), 3.12 – 3.00 (m, 1H, cod-H), 2.97 (sept, 1H, $CHMe_2$, $J$ = 6.7 Hz), 2.85 – 2.67 (m, 1H, cod-H), 2.66 – 2.50 (m, 1H, cod-H), 2.50 – 2.40 (m, 1H, cod-H), 2.40 – 2.32 (m, 1H, cod-H), 2.32 – 2.22 (m, 1H, cod-H), 2.21 – 2.10 (m, 1H, cod-H), 2.07 – 1.97 (m, 2H, cod-H), 1.96 – 1.88 (m, 1H, cod-H), 1.55 (d, 3H, $CH(CH_3)_2$, $J$ = 6.7 Hz), 1.53 – 1.40 (m, 6H, $C(CH_3)_2$), 1.32 (br, 1H, Ir-$CH_2$), 1.19 (d, 9H, $C(CH_3)_3$, $J$ = 13.8 Hz), 1.07 (d, 3H, $CH(CH_3)_2$, J = 6.7 Hz), 1.01 – 0.85 (m, overlapping signals, 2H, Ir-$CH_2$, Si-$CH_2$), 0.47 (d, 3H, $CH(CH_3)_2$, $J$ = 6.7 Hz), -0.09 (dd, 1H, Si-$CH_2$, J = 30.7, 12.2 Hz). $^{13}C\{^1H\}$ NMR (THF-$d_8$ 101 MHz, 25 °C): δ 168.31 (NCN), 145.47(Ar-C), 143.86(Ar-C), 141.47(Ar-C), 139.38 (Ar-C), 138.13 (Ar-C), 130.06 (Ar-C), 129.48 (Ar-C), 127.98 (Ar-C), 127.51 (Ar-C), 126.96 (Ar-C), 125.40 (Ar-C), 122.02 (Ar-C), 77.94 (cod-$CH_2$), 76.42 (cod-$CH_2$), 63.16 (cod-$CH_2$), 57.41 (cod-$CH_2$), 41.03 ($C(CH_3)_2(CH_2Ir)$), 36.96

($C(CH_3)_3$), 36.59 (cod-*C*H), 35.31 (cod-*C*H), 33.57 ($CH(CH_3)_2$), 32.90 (cod-*C*H), 32.24 (cod-*C*H), 28.79 ($CH(CH_3(CH_2Si))$), 28.66 ($CH(CH_3)_2$), 27.02 ($C(CH_3)_3$), 26.30 ($CH(CH_3(CH_2Si))$), 21.64 (overlapping signals, $C(CH_3)_2(CH_2Ir)$), 19.51 ($CH(CH_3(CH_2Si))$).$^{31}$P NMR (THF-$d_8$, 162 MHz, 25 °C): δ 60.75 (br). $^{11}$B{$^1$H} NMR (THF-$d_8$, 128 MHz, 25 °C): δ -58.93 (br). HRMS (ESI): m/z calcd for: 839.3673 [(M + H)]$^+$; found: 839.3658.

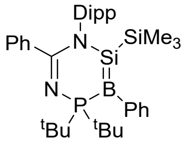


**Synthesis of 6.** TMSOTf (2 mL, 0.1M in toluene, 0.2 mmol) was added to a solution of **3** (0.116 g, 0.1 mmol) in benzene at room temperature. The reaction mixture was stirred for 15 mins to quantitatively form compound **6**, traced by $^1$H and $^{31}$P NMR spectroscopy. X-ray-crystallography-quality orange crystals (isolated yield: 0.021 g (17%)) were afforded from the concentrated filtrate. $^1$H NMR ($C_6D_6$, 400 MHz, 25 °C): δ 7.90 – 7.84 (m, 2H, Ar-H), 7.40 – 7.35 (m, 2H, Ar-H), 7.29 (t, 2H, Ar-H, *J* = 7.4 Hz), 7.11 (dd, 1H, Ar-H, *J* = 7.4, 1.7 Hz), 7.06 (t, 1H, Ar-H, *J* = 7.7 Hz), 6.97 – 6.78 (m, 5H, Ar-H), 3.52 (sept, 2H, C*H*Me$_2$, *J* = 6.9 Hz), 1.39 (d, 18H, $C(CH_3)_3$, *J* = 14.0 Hz), 1.34 (d, 6H, $CH(CH_3)_2$, *J* = 6.8 Hz), 0.93 (d, 6H, $CH(CH_3)_2$, *J* = 6.8 Hz), -0.09 (s, 9H, $(CH_3)_3$). $^{13}$C{$^1$H} NMR ($C_6D_6$, 101 MHz, 25 °C): δ 160.23 (d, N*C*N, *J* = 10.8 Hz), 145.63 (Ar-C), 141.02 (Ar-C), 140.59 (Ar-C), 140.45 (Ar-C), 135.87 (Ar-C), 129.55 (Ar-C), 128.45 (Ar-C), 127.19 (Ar-C), 126.95 (Ar-C), 125.22 (Ar-C), 124.90 (Ar-C), 123.61 (Ar-C), 37.85 (d, $C(CH_3)_3$, *J* = 43.2 Hz), 29.01 ($C(CH_3)_3$), 28.38 ($CH(CH_3)_2$), 28.36 ($CH(CH_3)_2$), 28.02 ($CH(CH_3)_2$), 27.87 ($CH(CH_3)_2$), 25.26 ($C(CH_3)_3$), 24.47 ($CH(CH_3)_2$), 23.70 ($C(CH_3)_3$), 21.93 ($CH(CH_3)_2$), 2.32 ($Si(CH_3)_3$). $^{31}$P{$^1$H} NMR ($C_6D_6$, 162 MHz, 25 °C): 49.66 (br). $^{11}$B{$^1$H} NMR ($C_6D_6$, 128 MHz, 25 °C): δ 25.30 (br). $^{29}$Si{$^1$H} ($C_6D_6$, 79 MHz, 25 °C): δ 110.06 (*Si*=B), -12.64 (d, *Si*Me$_3$, *J* = 12.9 Hz). HRMS (ESI): m/z calcd for: 613.3734 [(M + H)]$^+$; found: 613.3758.

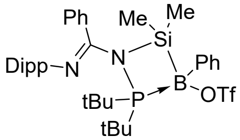


**Synthesis of 7.** MeOTf (2 mL, 0.1M in toluene, 0.2 mmol) was added to a solution of **3** (0.116 g, 0.1 mmol) in benzene at room temperature. The reaction mixture was stirred for 50 mins and resulting suspension was filtered to quantitatively form compound **7**, traced by $^1$H and $^{31}$P NMR spectroscopy. X-ray-crystallography-quality colorless crystals (isolated yield: 0.0448 g (28%)) were afforded from the concentrated filtrate. $^1$H NMR (THF-$d_8$, 400 MHz, 25 °C): δ 7.60 – 7.56 (m, 2H, Ar-H), 7.32 – 7.20 (m, 5H, Ar-H), 7.20 – 7.10 (m, 3H, Ar-H), 7.08 – 6.97 (m, 1H, Ar-H), 6.85 – 6.71 (m, 2H, Ar-H), 3.34 (sept, 1H, C*H*Me$_2$, *J* = 6.9 Hz), 2.80 (sept, 1H, C*H*Me$_2$, *J* = 7.6 Hz), 1.92 (d, 7H, $C(CH_3)_3$, *J* = 15.1 Hz), 1.63 (d, 2H, $C(CH_3)_3$, *J* = 13.8 Hz), 1.37 (overlapping signals, 10H, $CH(CH_3)_2$, $C(CH_3)_3$), 1.19 (d, 3H, $CH(CH_3)_2$, *J* = 6.8 Hz), 0.98 (d, 2H, $C(CH_3)_3$, *J* =13.6 Hz), 0.89 (d, 3H, $CH(CH_3)_2$, *J* = 6.8 Hz), 0.70 (d, 3H, $CH(CH_3)_2$, *J* = 6.9 Hz), 0.36 (s, 3H, $Si(CH_3)_2$), 0.16 (s, 3H, $Si(CH_3)_2$). $^{13}$C{$^1$H} NMR (THF-$d_8$, 101 MHz, 25 °C): δ160.55 (d, N*C*N, *J* = 8.5 Hz), 144.16 (Ar-C), 140.25 (Ar-C), 137.75 (Ar-C), 137.04 (d, Ar-C, *J* = 9.6 Hz), 130.54 (Ar-C), 129.75 (Ar-C), 129.08 (Ar-C), 128.77 (Ar-C), 128.55 (Ar-C), 128.12 (Ar-C), 124.62 (Ar-C), 124.26 (Ar-C), 123.69 (O-$CF_3$), 41.06 (d, $C(CH_3)_3$, *J* = 13.9 Hz), 40.69 (d, $C(CH_3)_3$, *J* = 4.7 Hz), 29.71 ($CH(CH_3)_2$), 29.42 ($C(CH_3)_3$), 28.73 ($CH(CH_3)_2$), 26.81 ($CH(CH_3)_2$), 26.21 ($CH(CH_3)_2$), 26.02 ($C(CH_3)_3$), 23.01 ($CH(CH_3)_2$), 22.86 ($CH(CH_3)_2$), 4.29 (d, $Si(CH_3)_2$, *J* = 8.1 Hz), 2.69 (d, $Si(CH_3)_2$, J = 4.3 Hz). $^{31}$P NMR (THF-$d_8$, 162 MHz, 25 °C): δ 87.11. $^{31}$P solid state NMR (202 MHz, 25 °C): δ 89.60. $^{11}$B and $^{29}$Si solution state NMR signals cannot be obtained. $^{11}$B solid state NMR (160 MHz, 25 °C): 1.73 (m). $^{29}$Si solid state NMR (99 MHz, 25 °C): 31.70 (m). HRMS (ESI): m/z calcd for: 719.3251 [(M + H)]$^+$; found: 719.3262.

## S2. Selected NMR Spectra

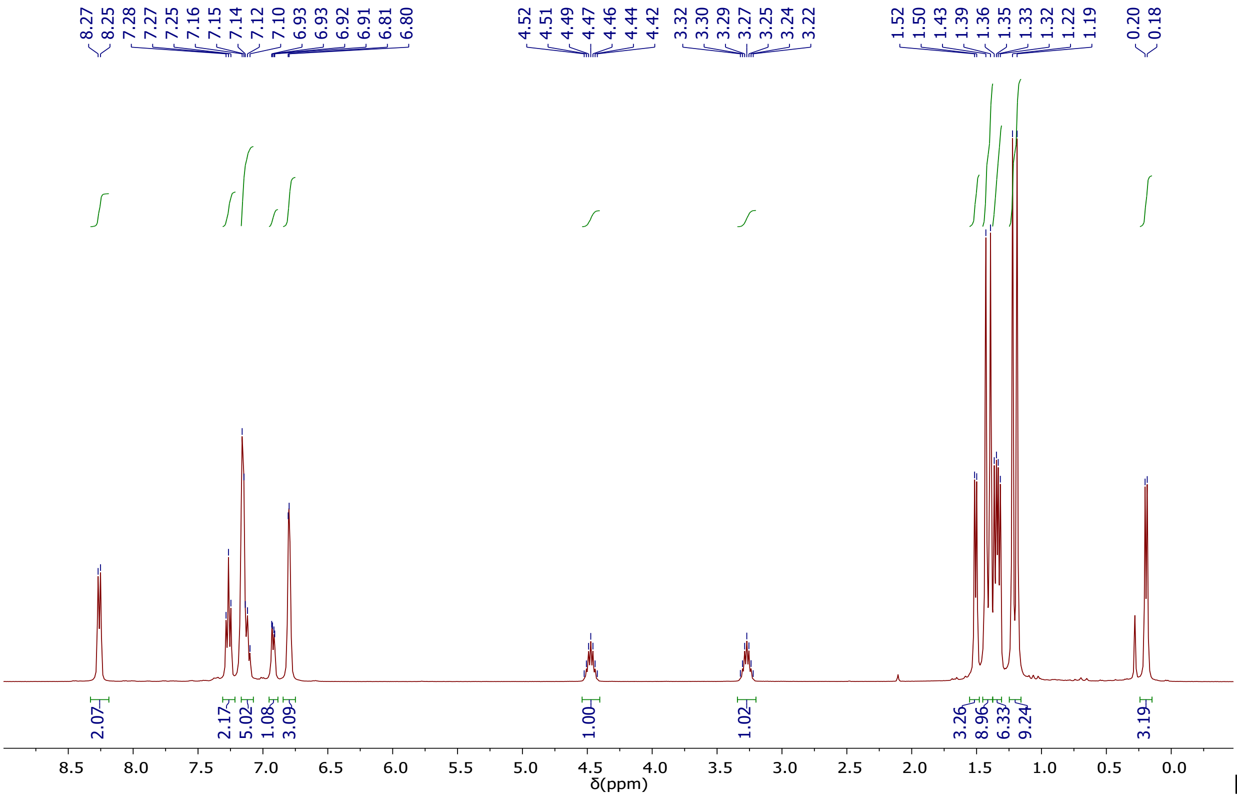


**F**

**igure S1.** $^1$H NMR spectrum of **2**.

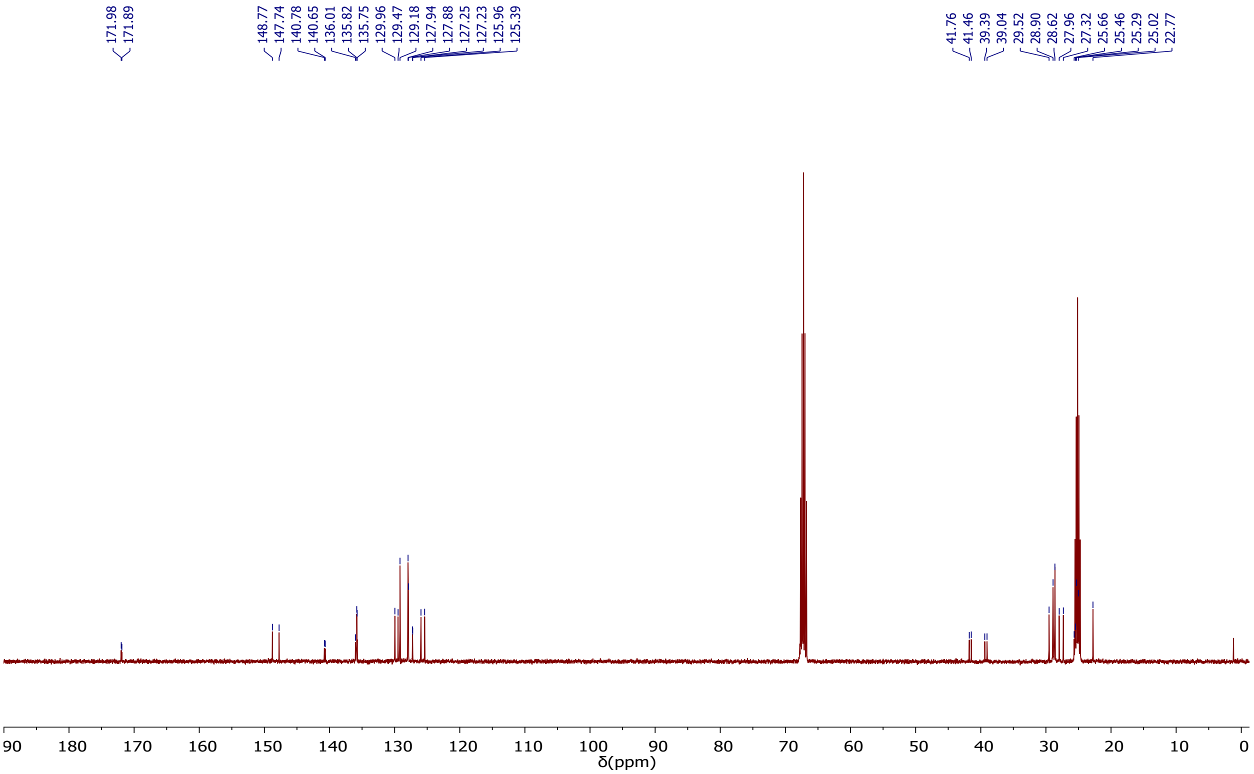


**Figure S2.** $^{13}C\{^{1}H\}$ NMR spectrum of **2**.

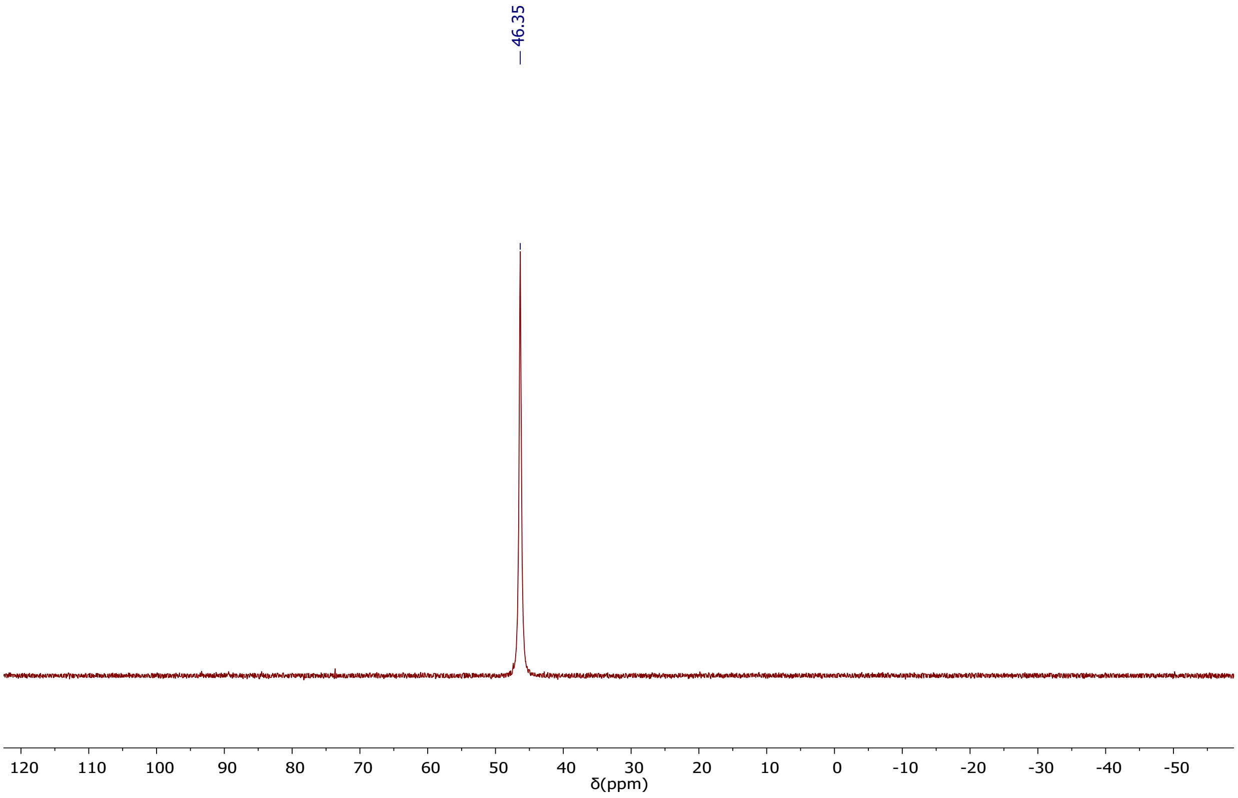


**Figure S3.** $^{31}P\{^{1}H\}$ NMR spectrum of **2**.

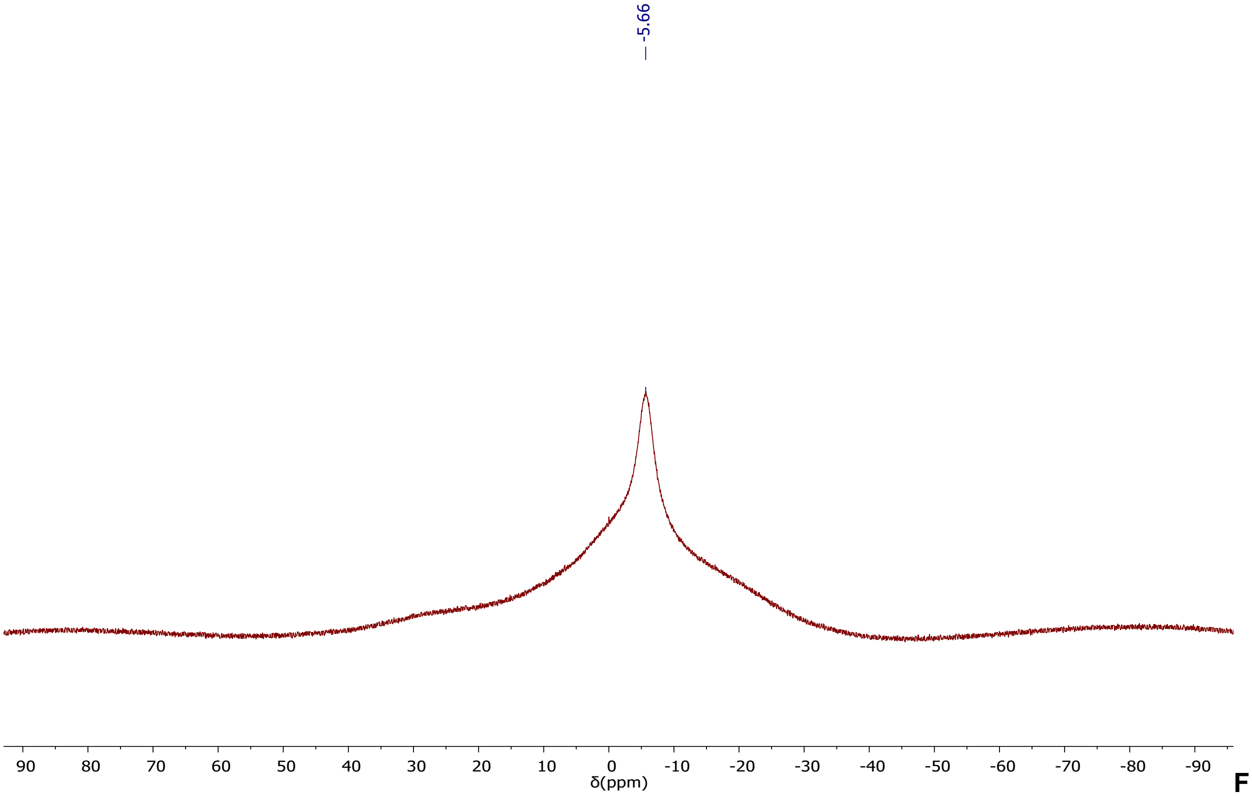


**F**

**igure S4.** $^{11}B\{^{1}H\}$ NMR spectrum of **2**.

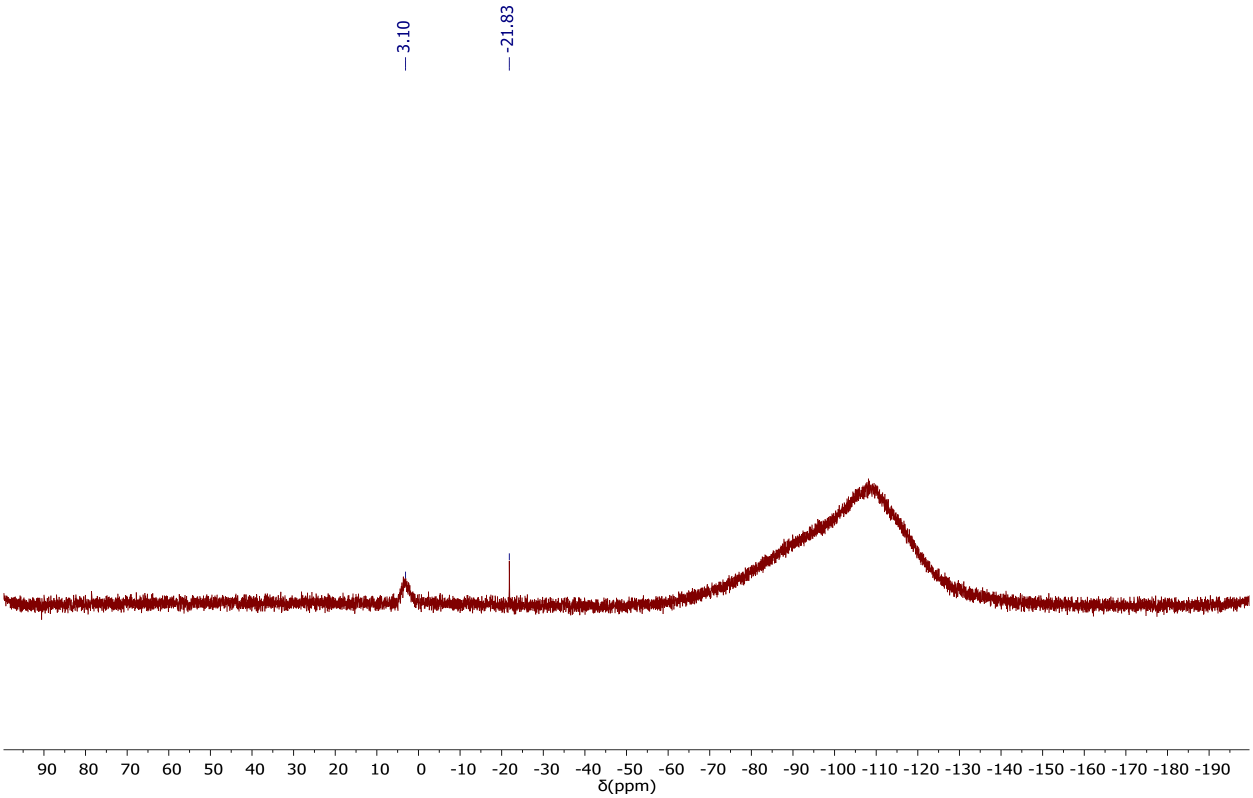


**Figure S5.** $^{29}Si\{^{1}H\}$ NMR spectrum of **2** (peak at -21.83 ppm corresponding to silicone grease).

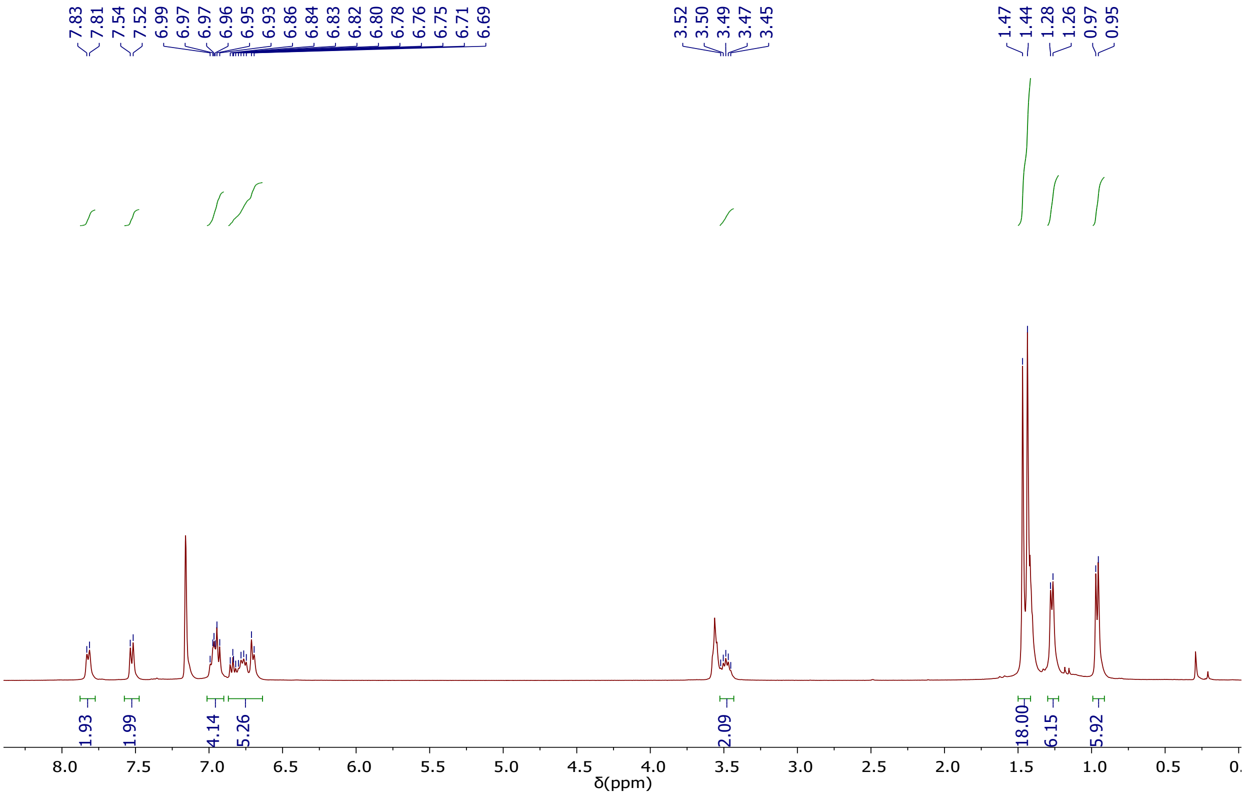


**Figure S6.** $^{1}H$ NMR spectrum of **3**.

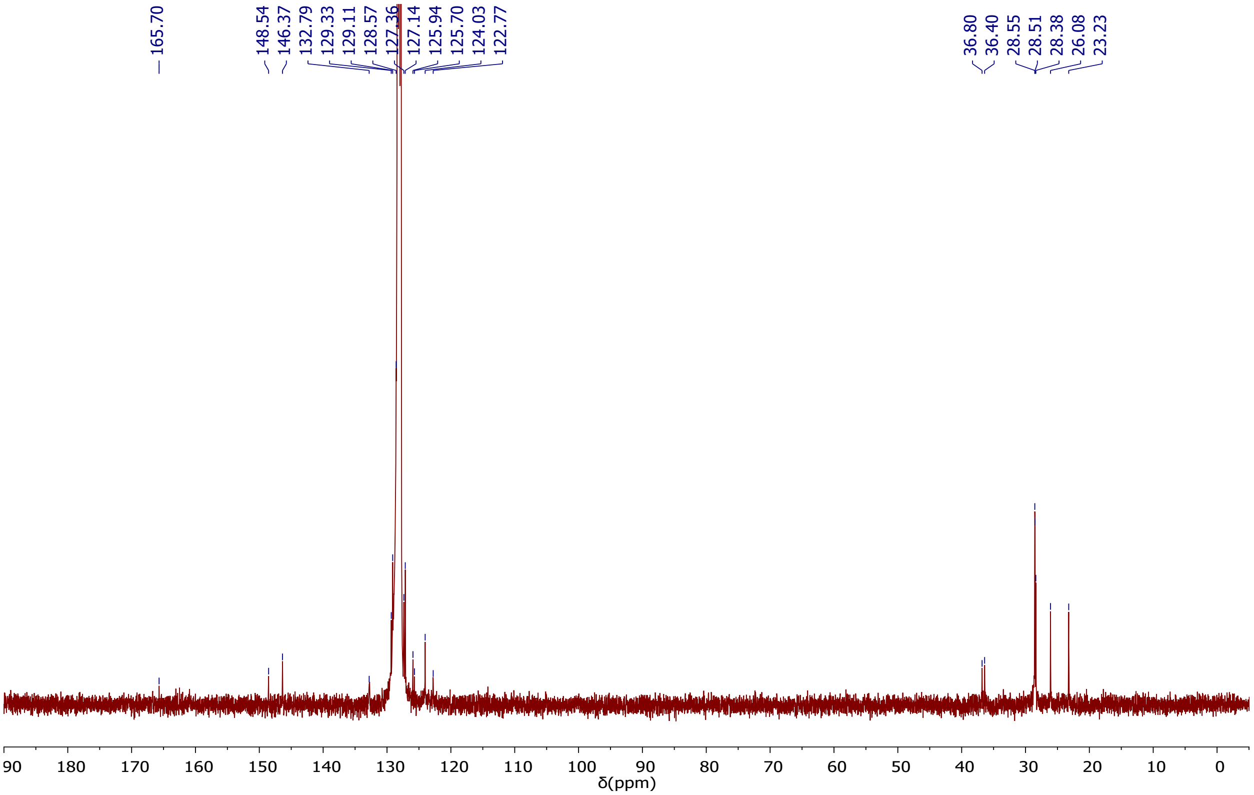


**Figure S7.** $^{13}C\{^{1}H\}$ NMR spectrum of **3**.

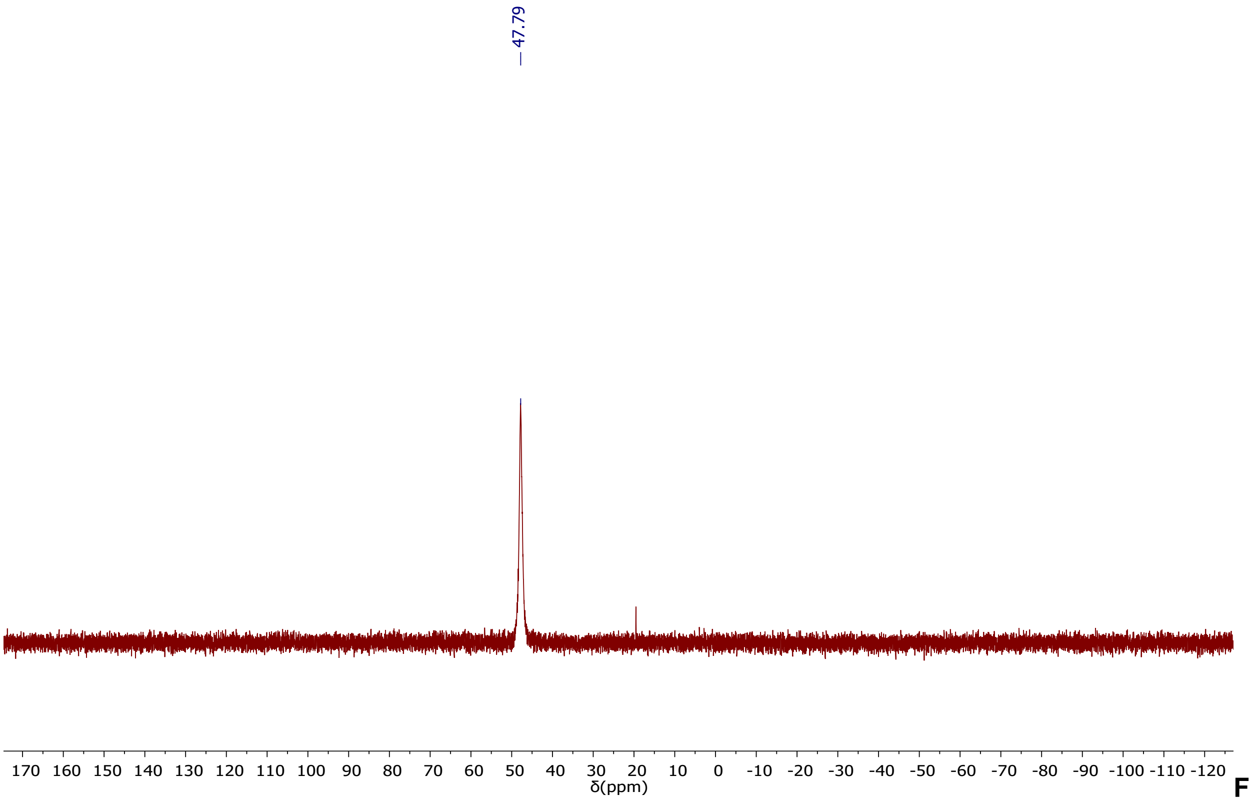


**F**

**igure S8.** $^{31}P\{^{1}H\}$ NMR spectrum of **3**.

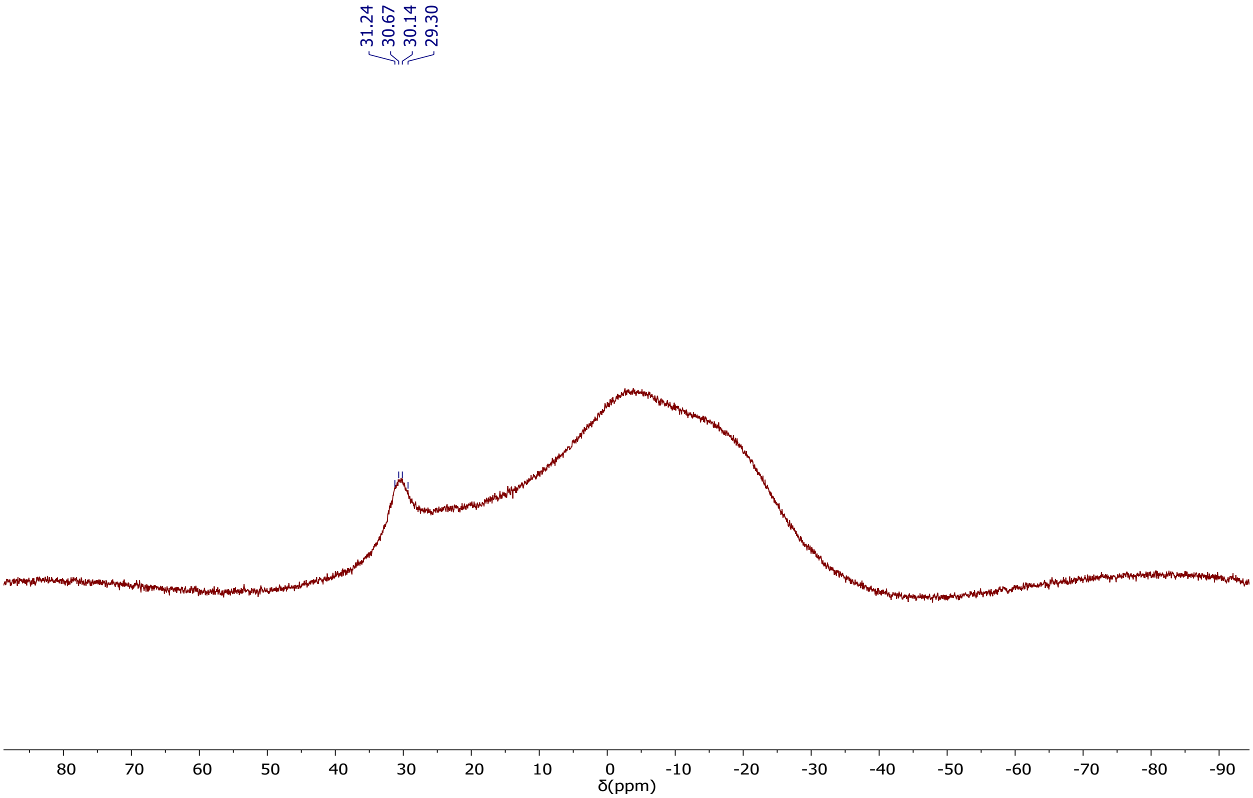


**Figure S9.** $^{11}B\{^{1}H\}$ NMR spectrum of **3**.

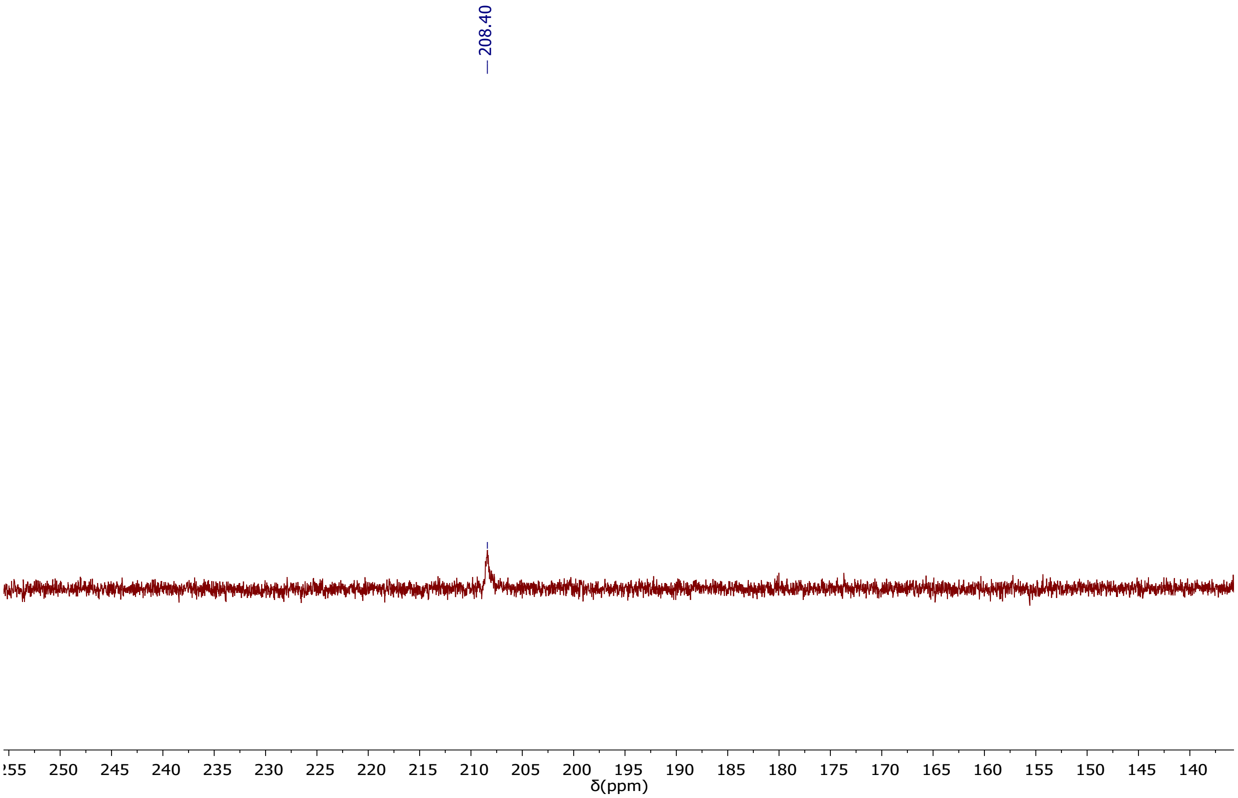


**Figure S10.** $^{29}Si\{^{1}H\}$ NMR spectrum of **3**.

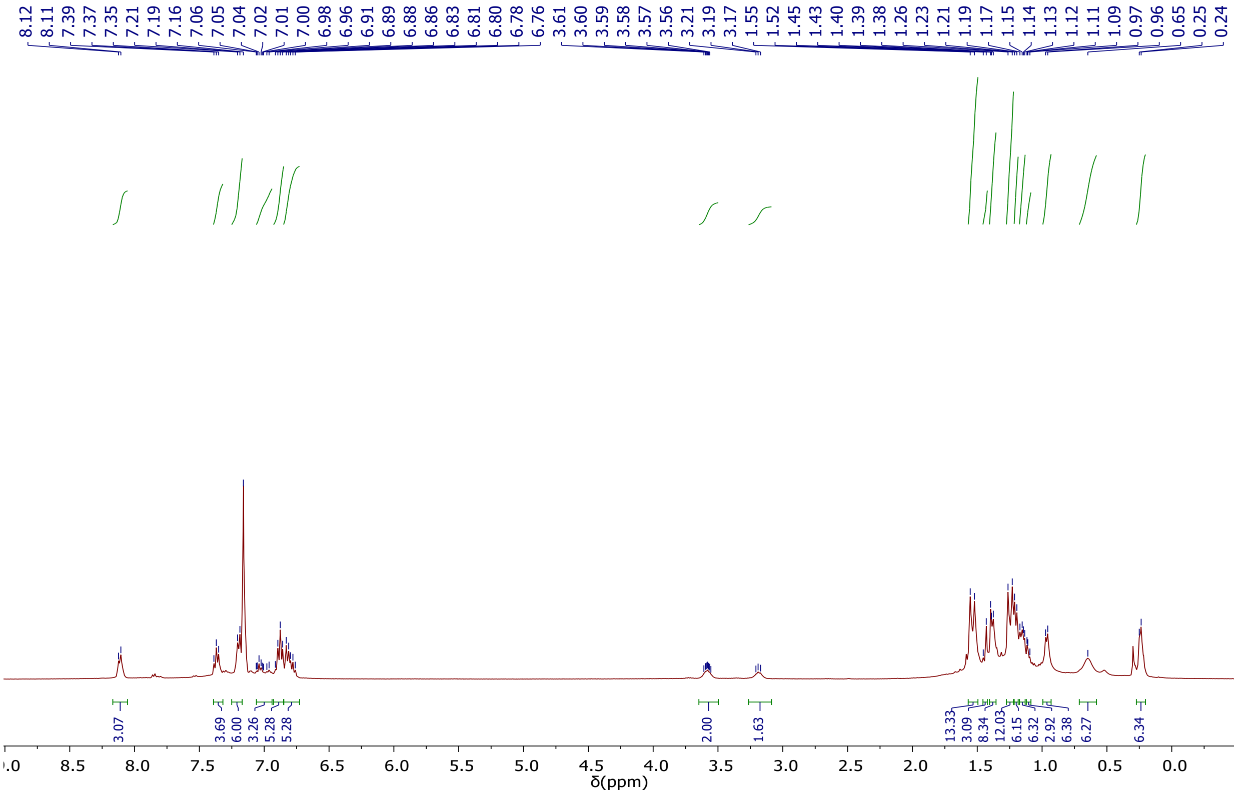


**Figure S11.** $^{1}H$ NMR spectrum of **4**.

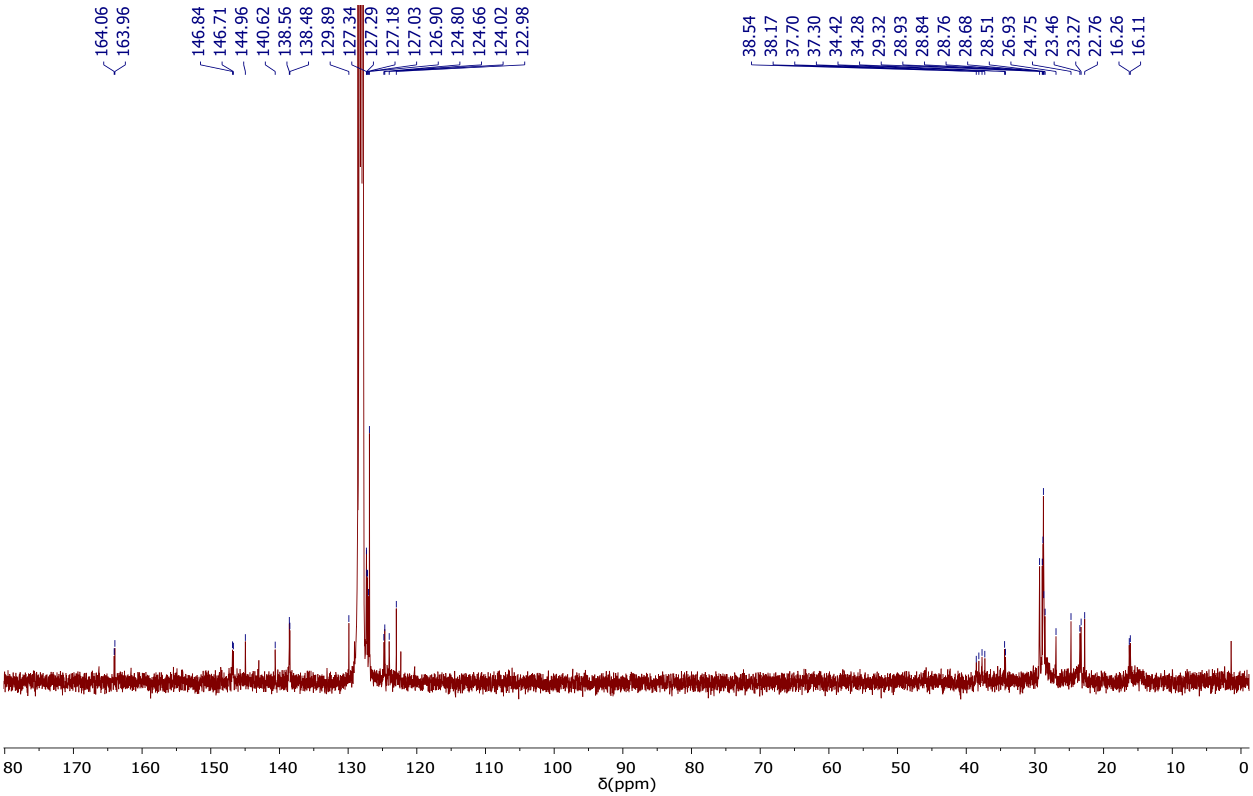


**Figure S12.** $^{13}C\{^{1}H\}$ NMR spectrum of **4**.

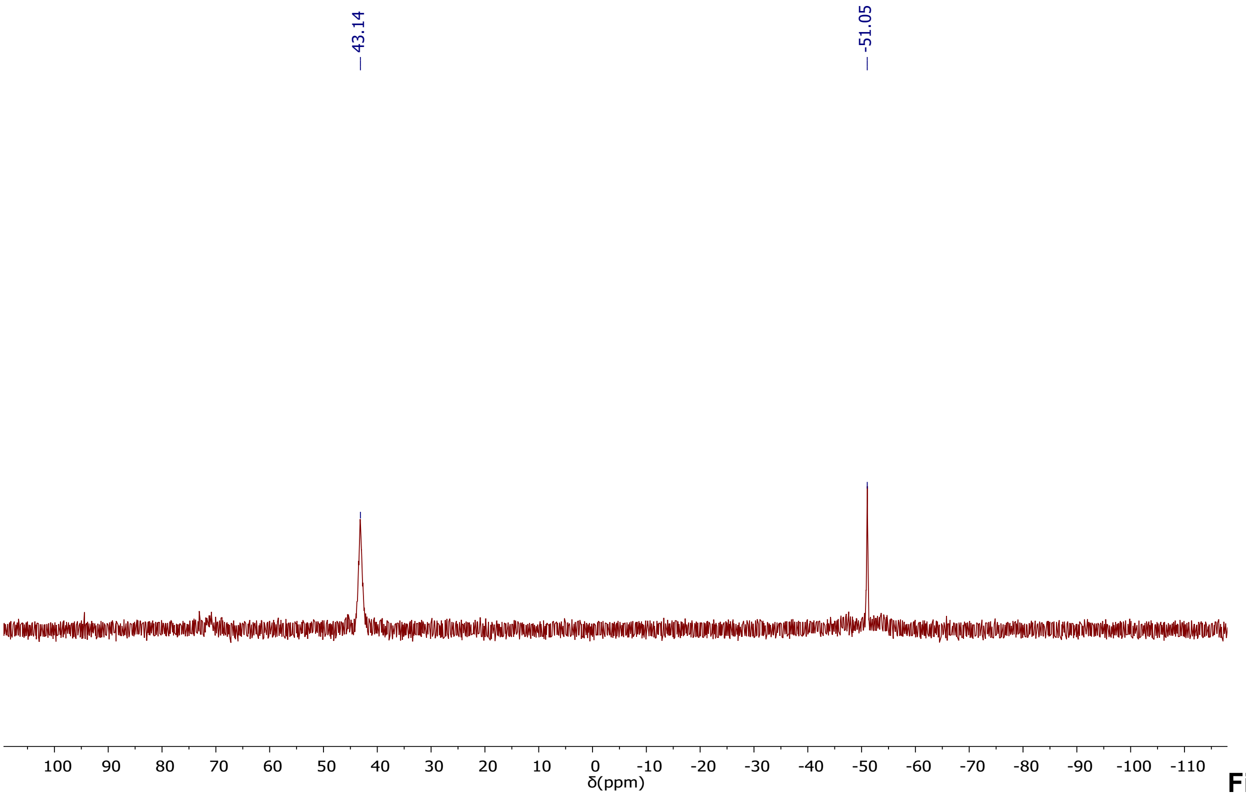


Fi

gure S13. $^{31}P\{^{1}H\}$ NMR spectrum of **4**.

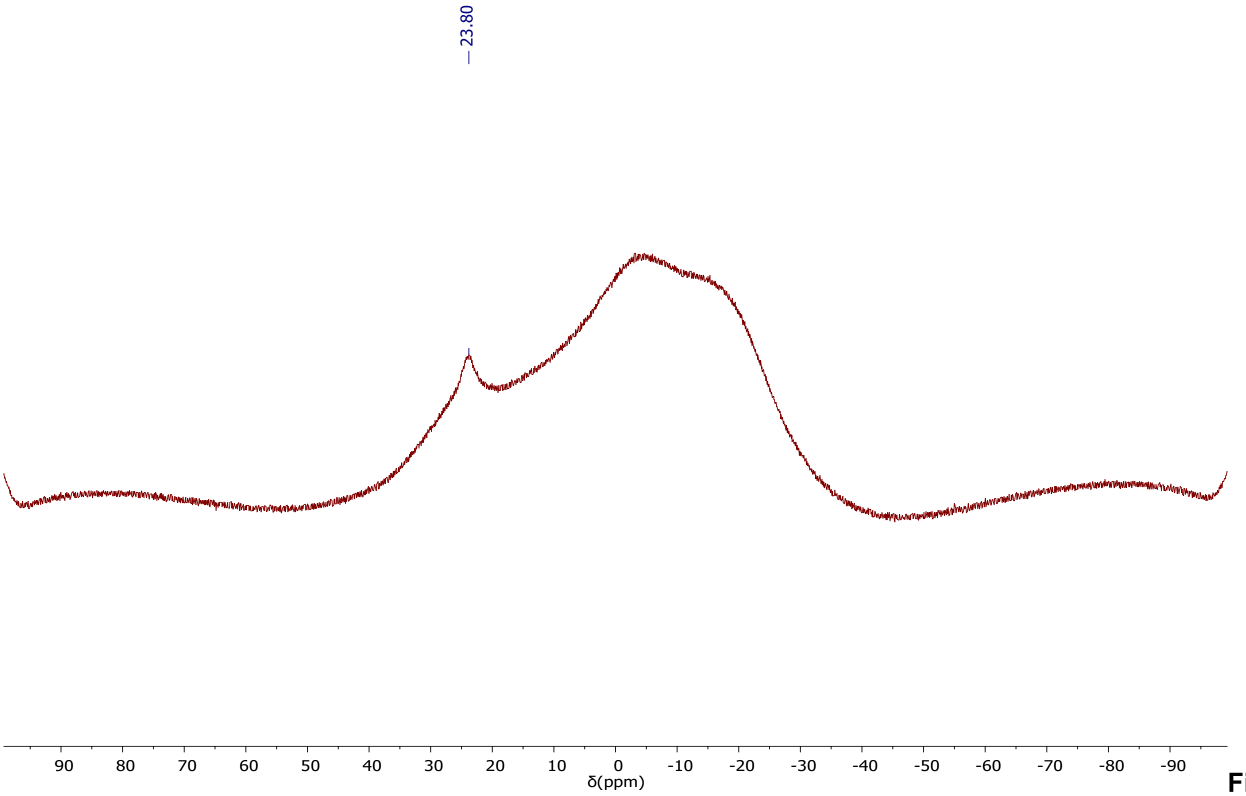


Fi

gure S14. $^{11}B\{^{1}H\}$ NMR spectrum of **4**.

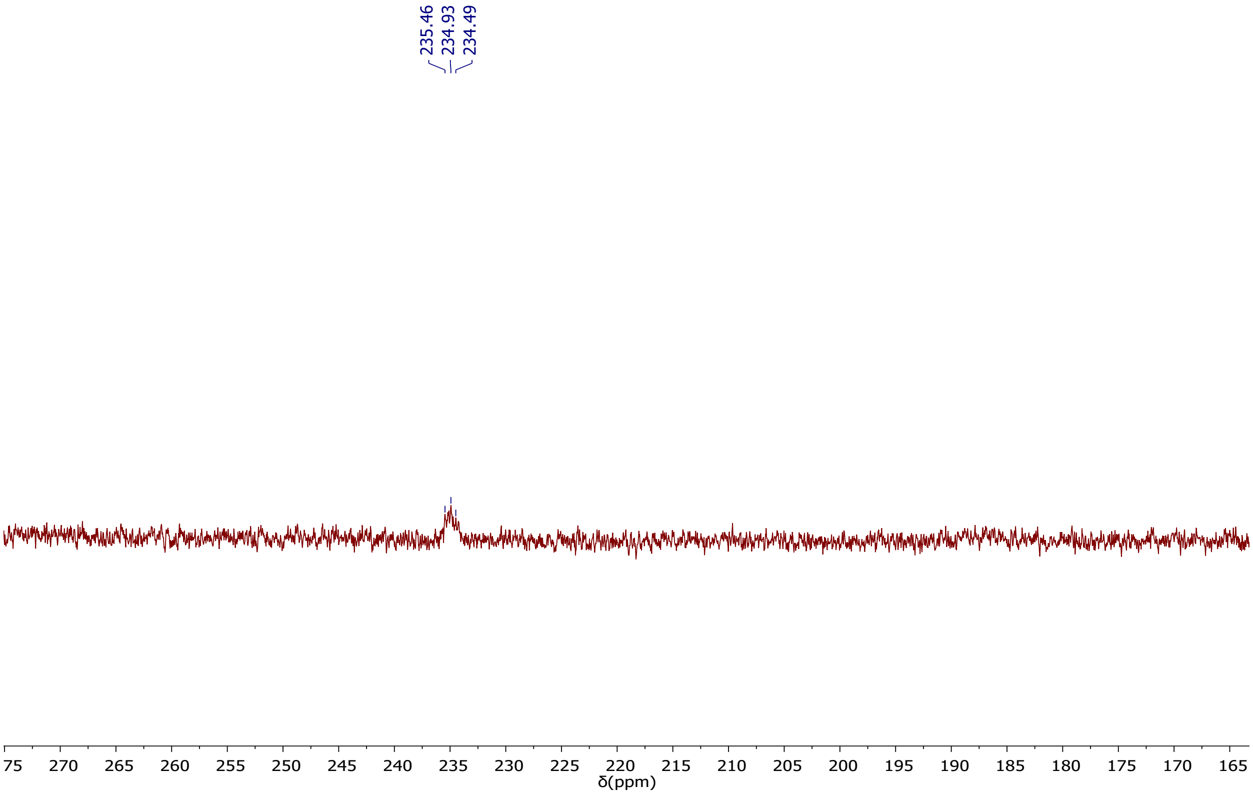

**Figure S15.** $^{29}Si\{^{1}H\}$ NMR spectrum of **4**.

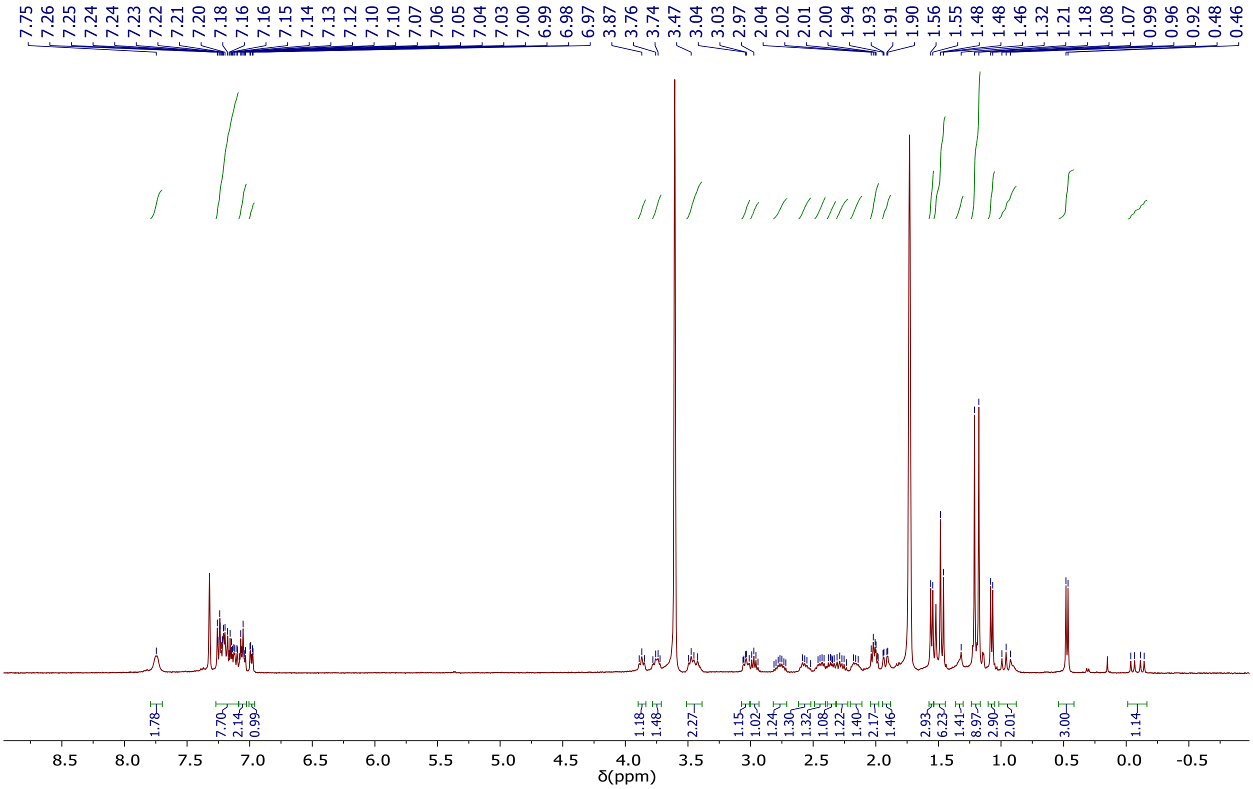

**Figure S16.** $^{1}H$ NMR spectrum of **5**.

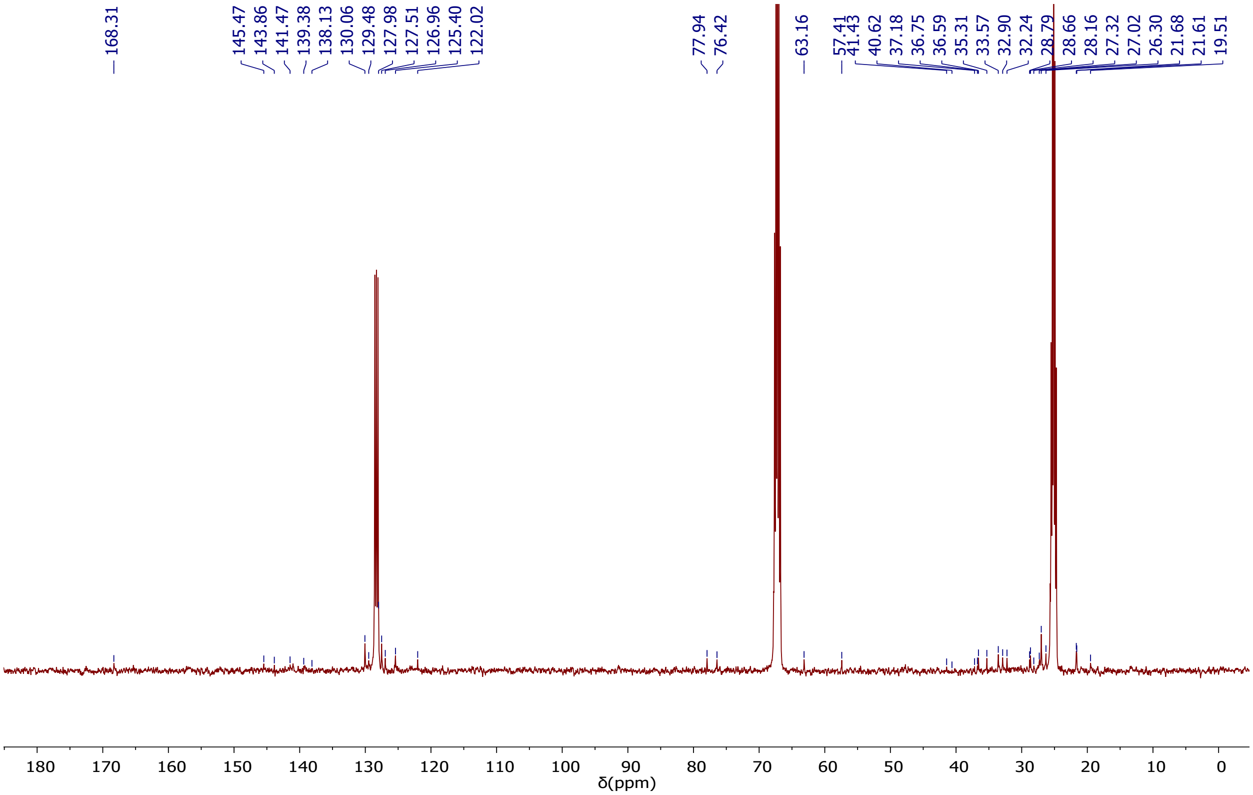


**Figure S17.** $^{13}C\{^{1}H\}$ NMR spectrum of **5**.

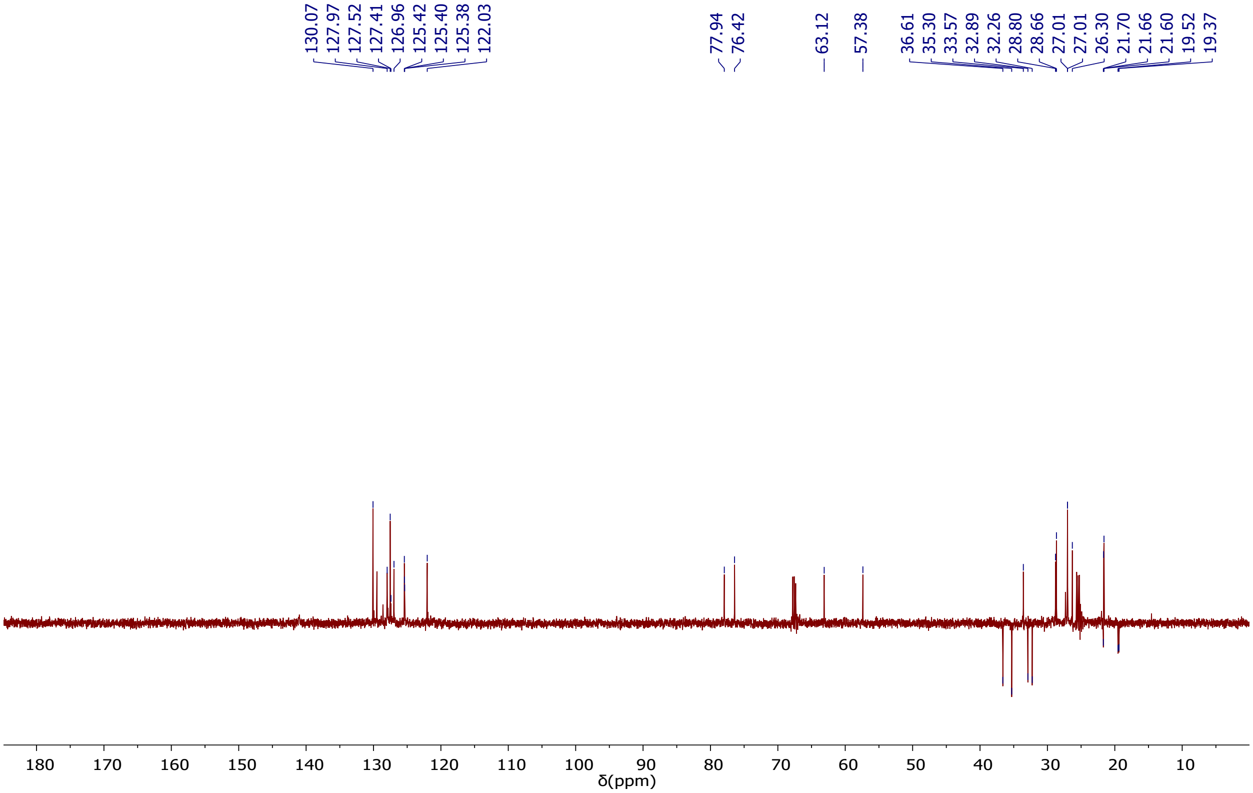


**Figure S18.** $^{13}C$ (DEPT135) NMR spectrum of **5**.

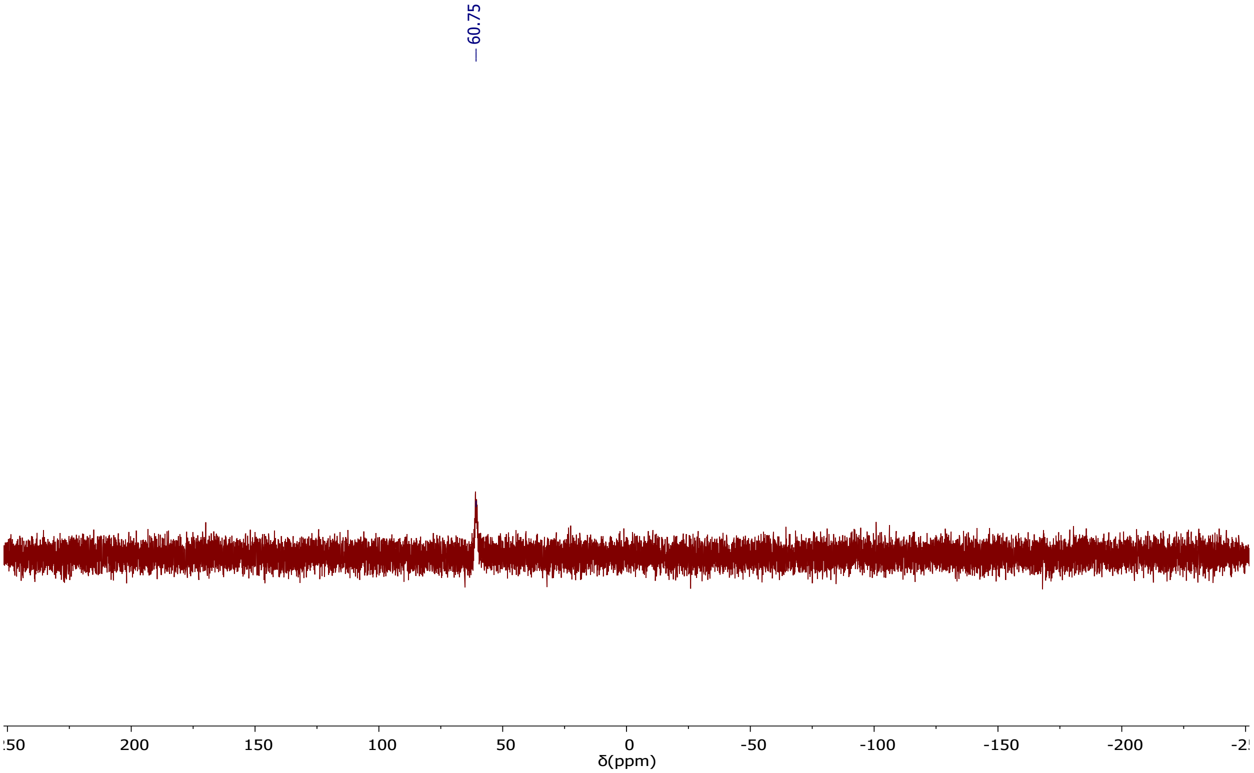


**Figure S19.** $^{31}$P NMR spectrum of **5**.

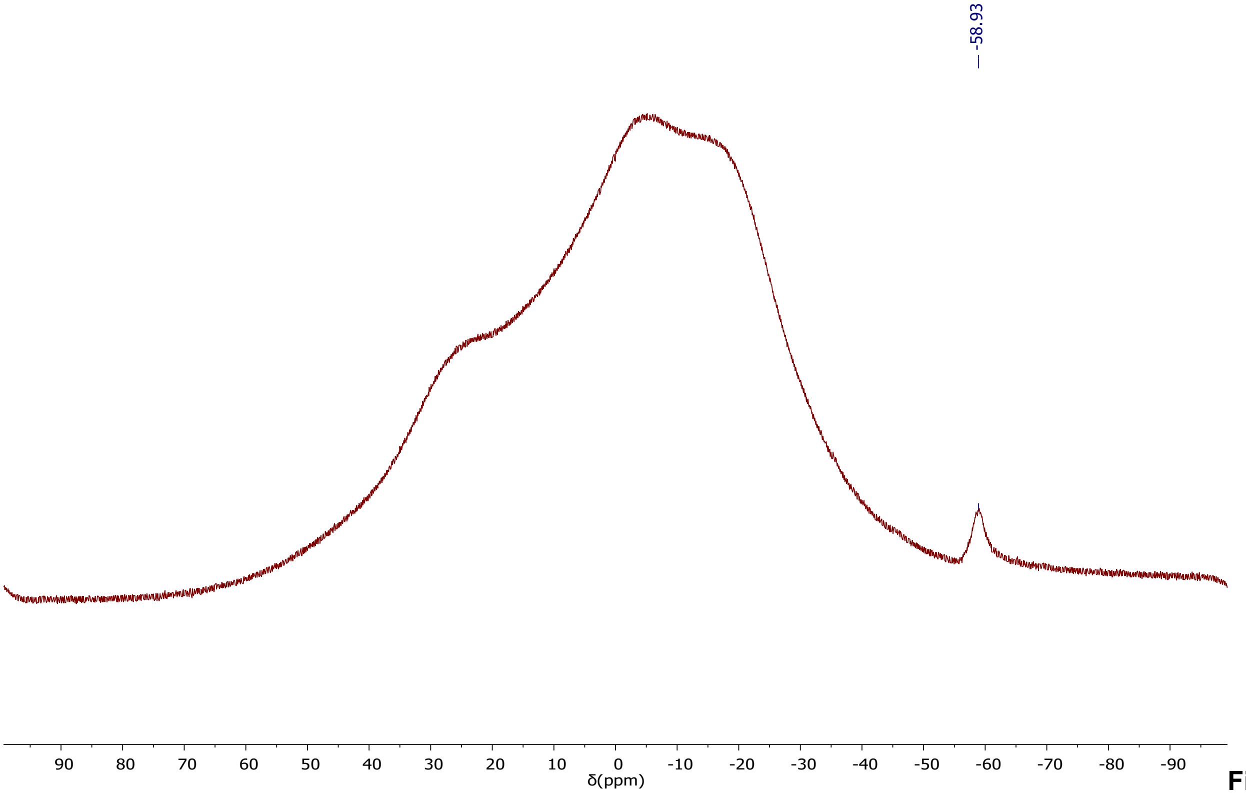


**Figure S20.** $^{11}$B{$^{1}$H} NMR spectrum of **5**.

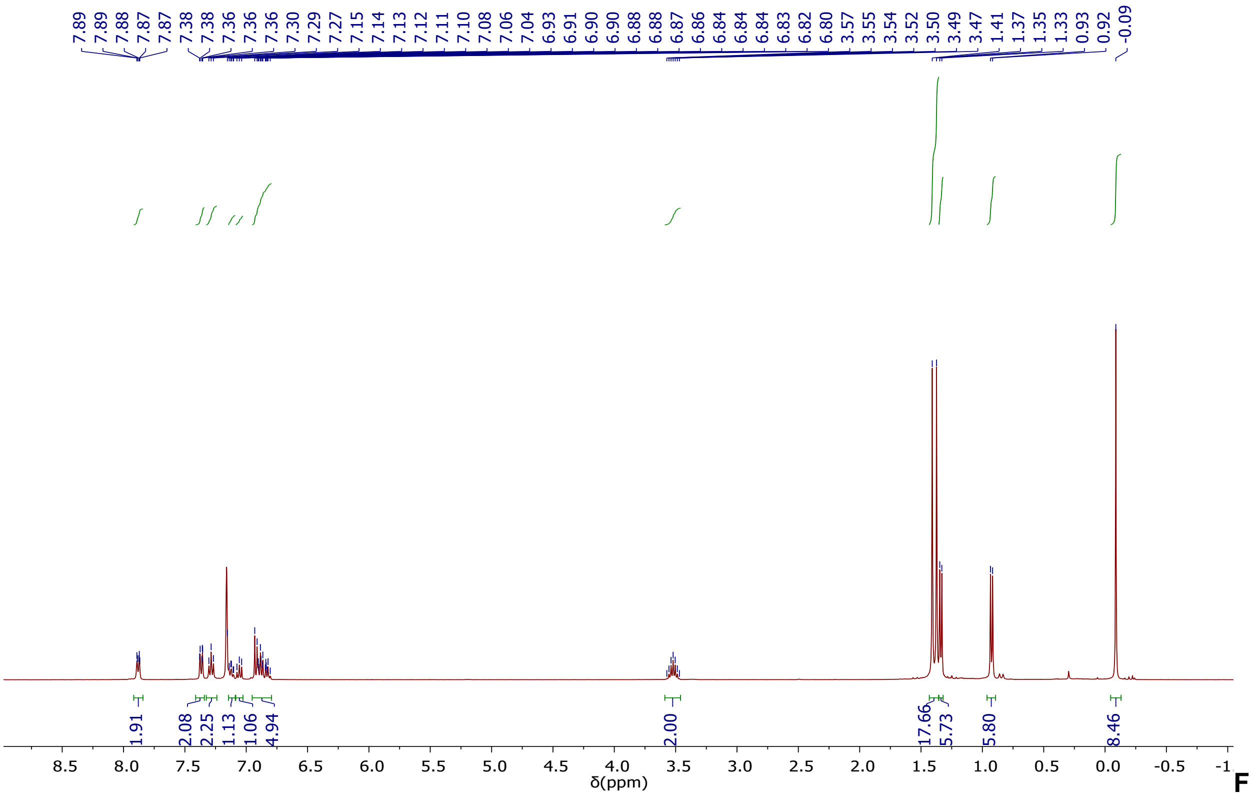


**Figure S21.** $^{1}$H NMR spectrum of **6**.

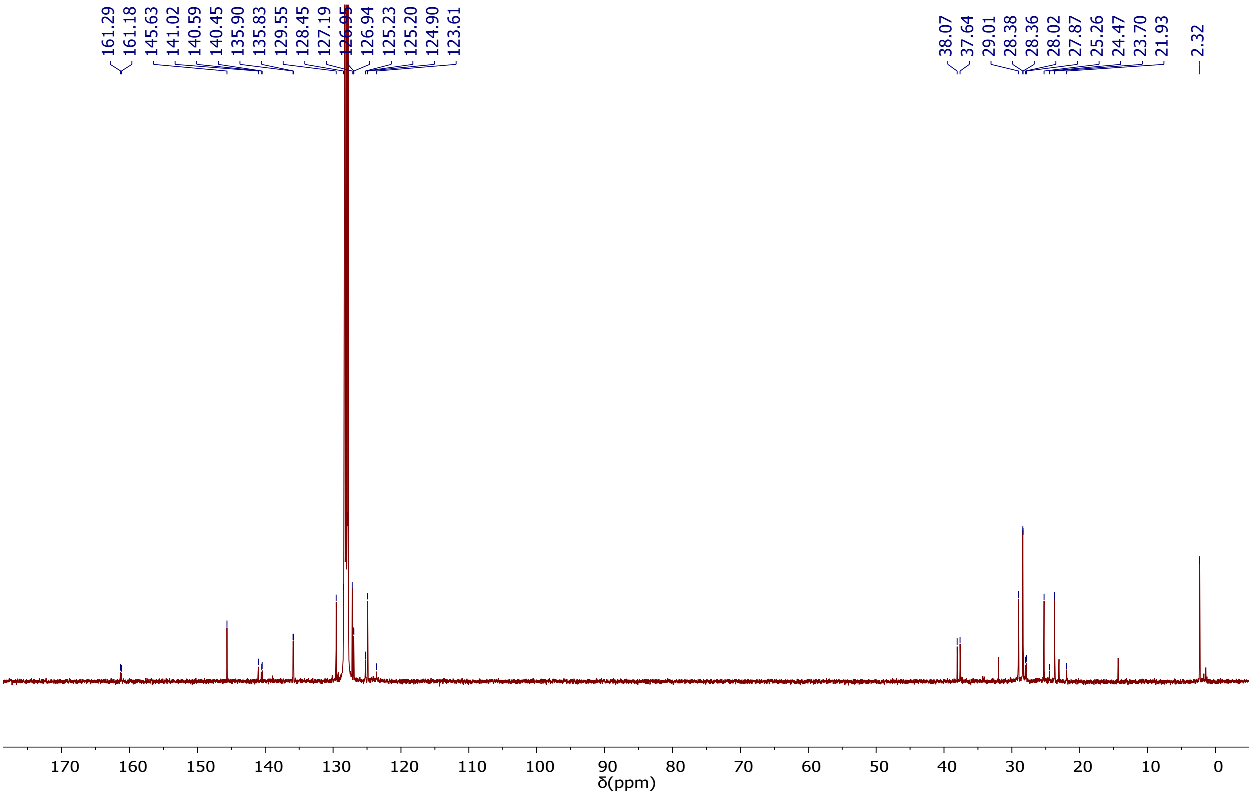


**Figure S22.** $^{13}$C{$^{1}$H} NMR spectrum of **6**.

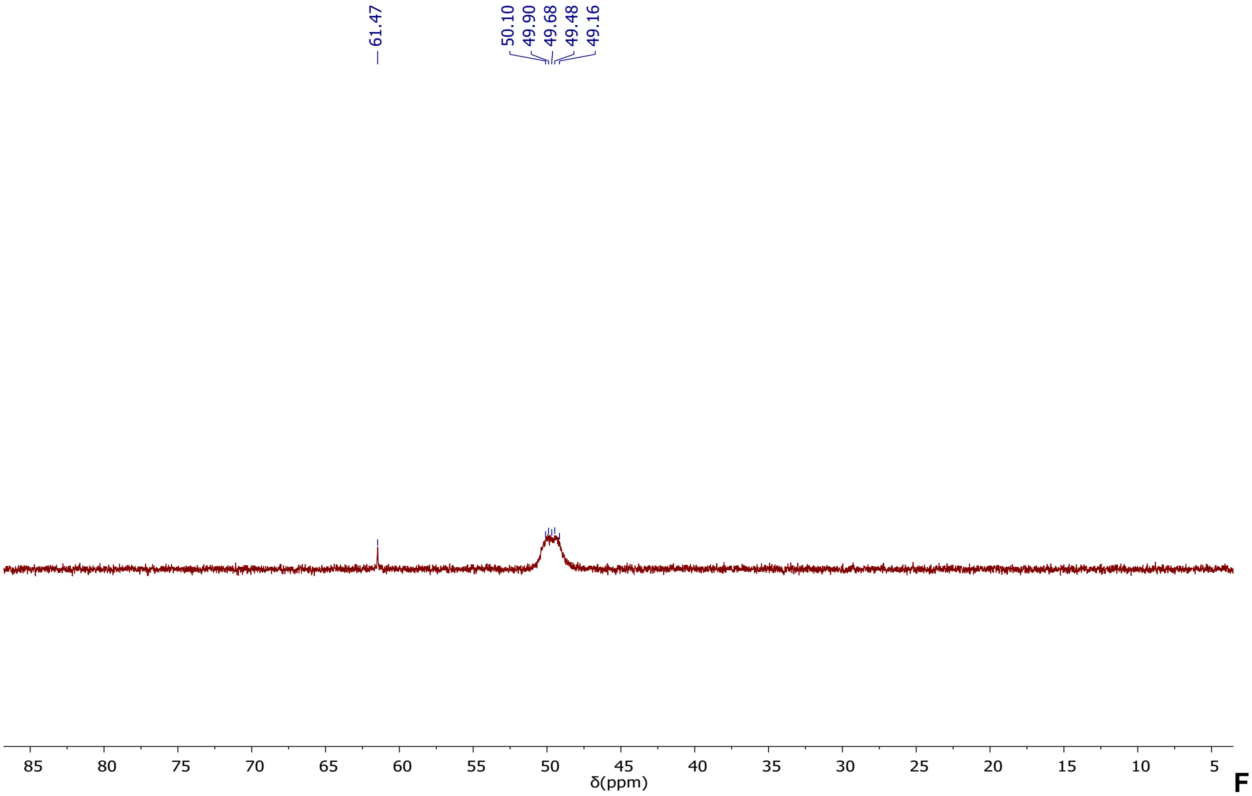


F

**igure S23.** $^{31}P\{^{1}H\}$ NMR spectrum of **6** (peak at 61.47 ppm corresponding to free *N*-phosphinoamidine ligand).

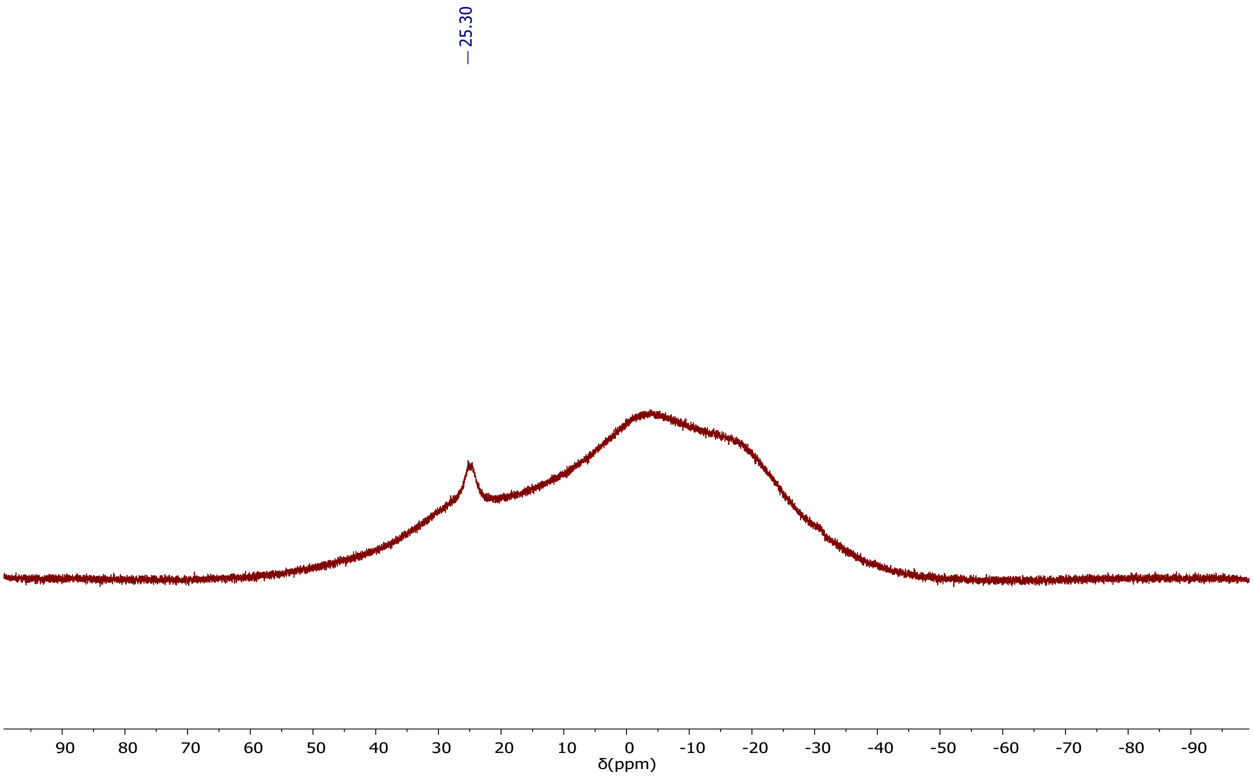


**Figure S24.** $^{11}B\{^{1}H\}$ NMR spectrum of **6**.

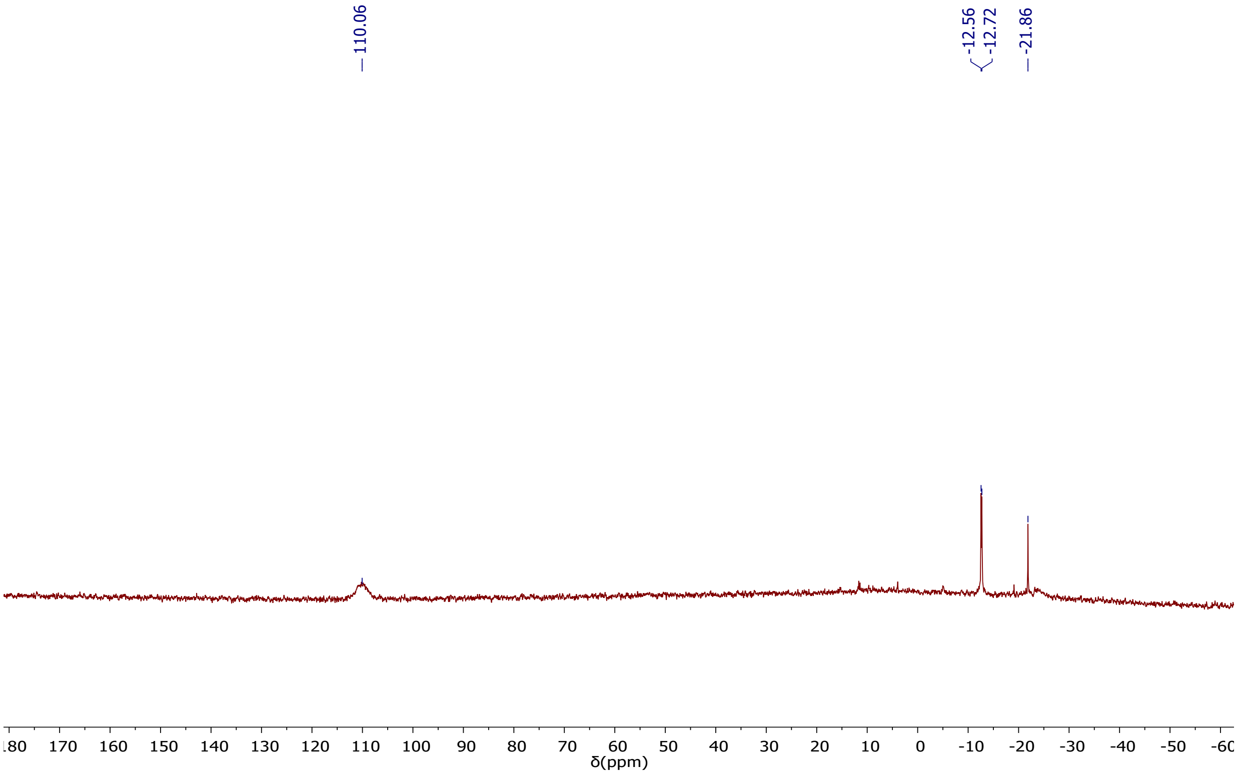


**Figure S25.** $^{29}Si\{^1H\}$ NMR spectrum of **6** (peak at -21.86 ppm corresponding to silicone grease).

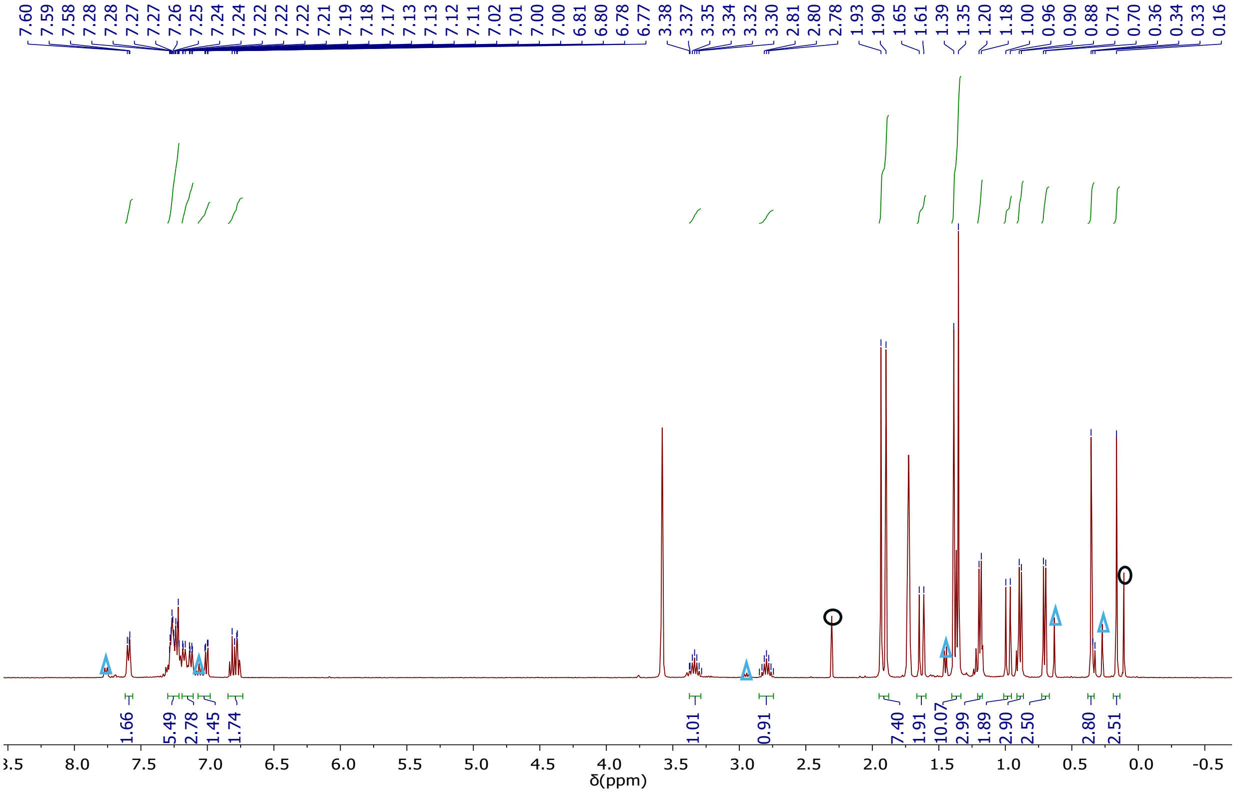


**Figure S26.** $^1H$ NMR spectrum of **7**. (Circled signals corresponds to solvent/ silicon grease, triangled signals indicate slight decomposition)

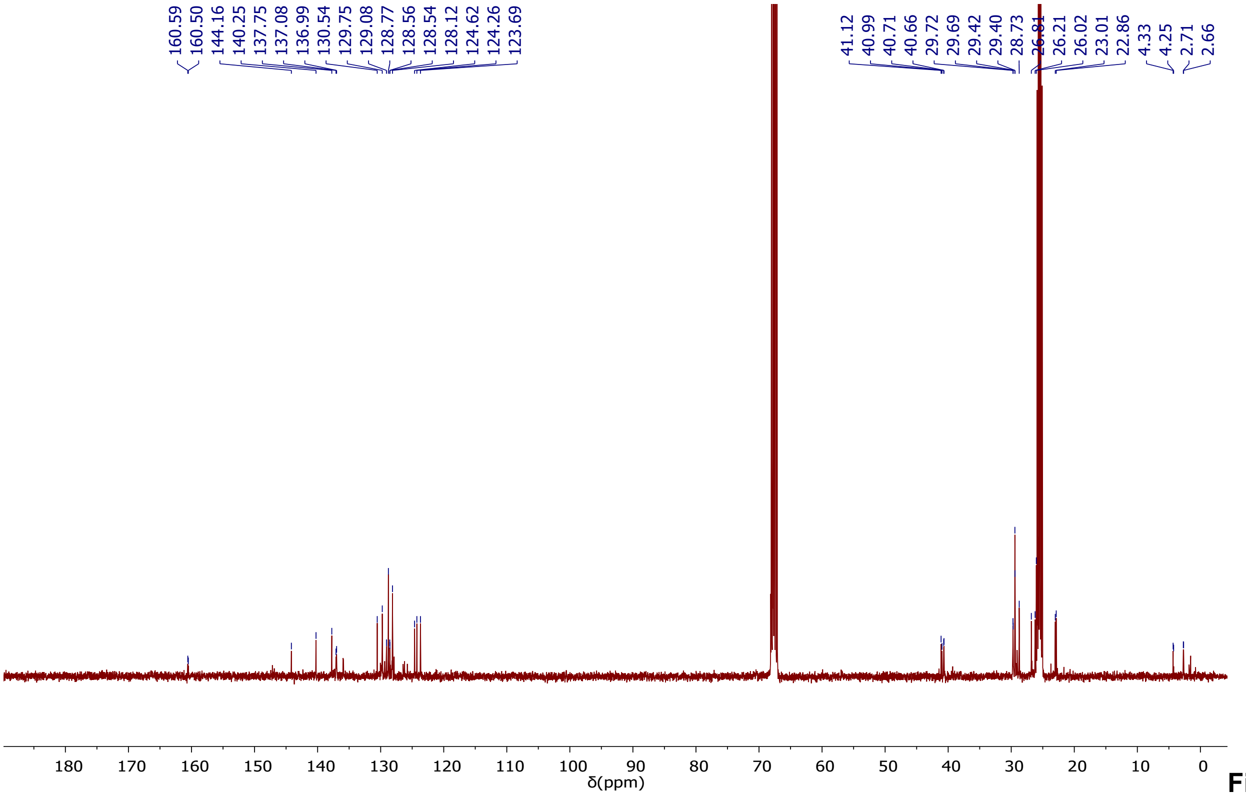


Fi

**gure S27.** $^{13}C\{^{1}H\}$ NMR spectrum of **7**.

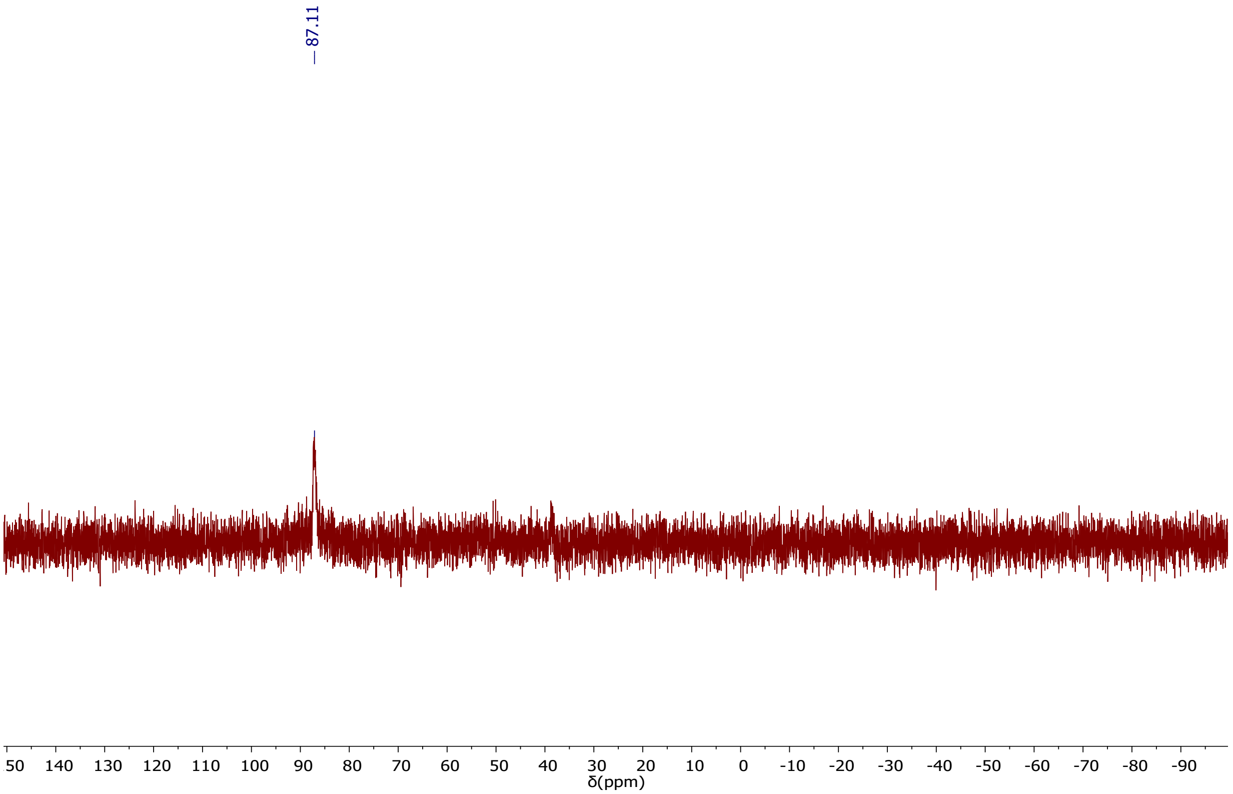


**Figure S28.** $^{31}P$ NMR spectrum of **7**.

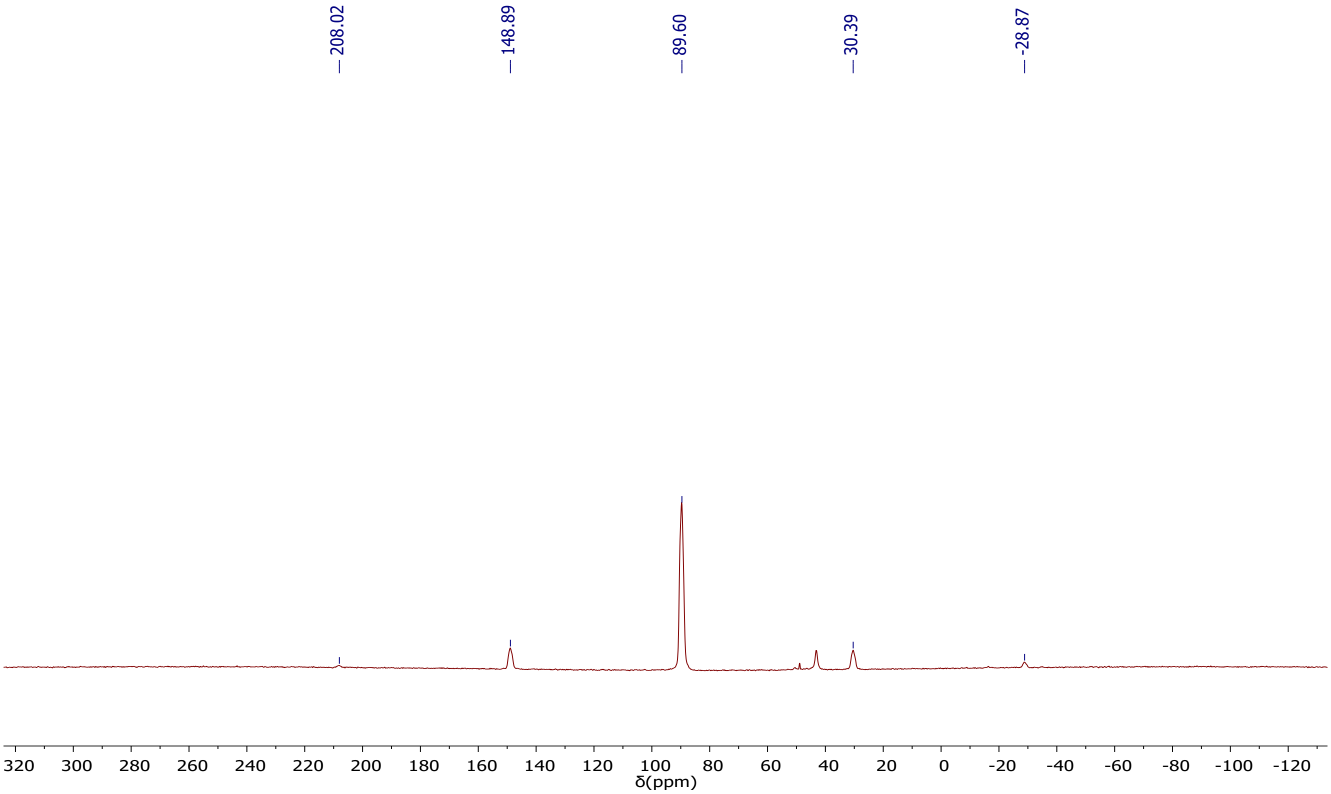


**Figure S29.** Solid-state $^{31}P$ NMR spectrum of **7**.

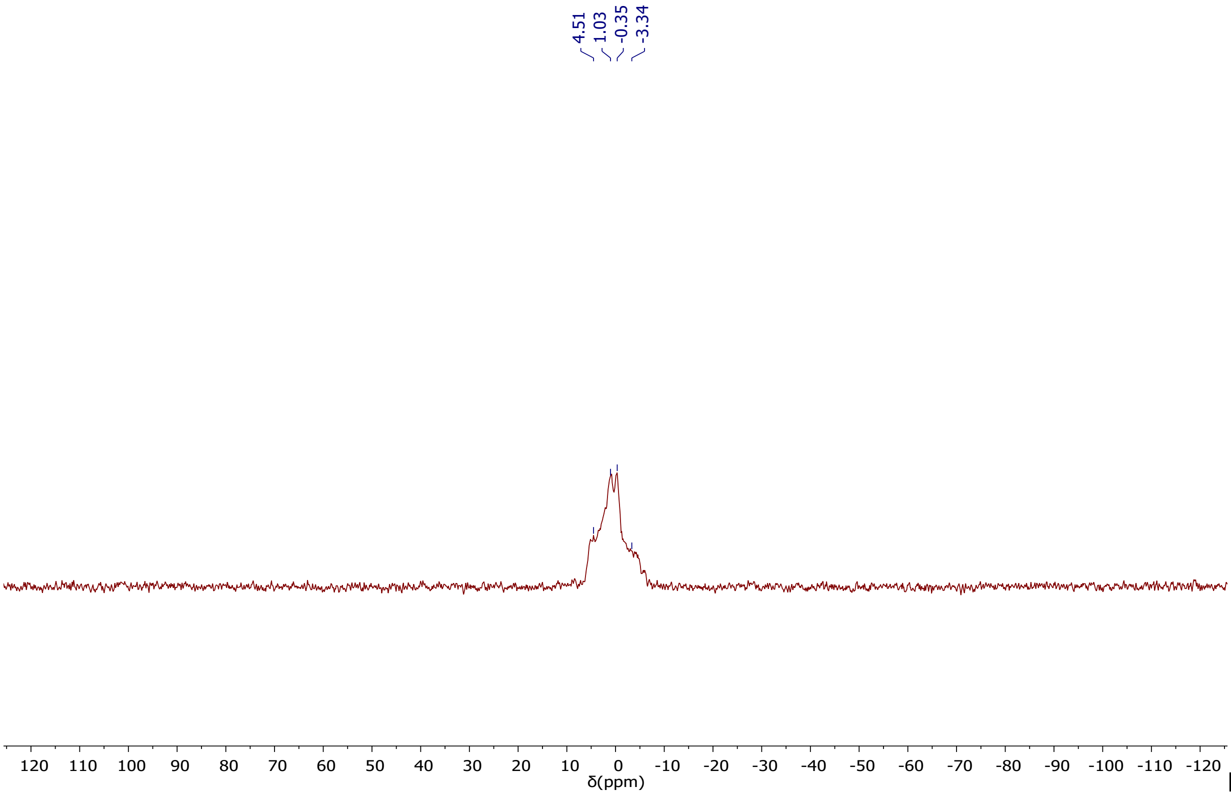


**Fi**

**gure S30.** Solid-state $^{11}B$ NMR spectrum of **7**.

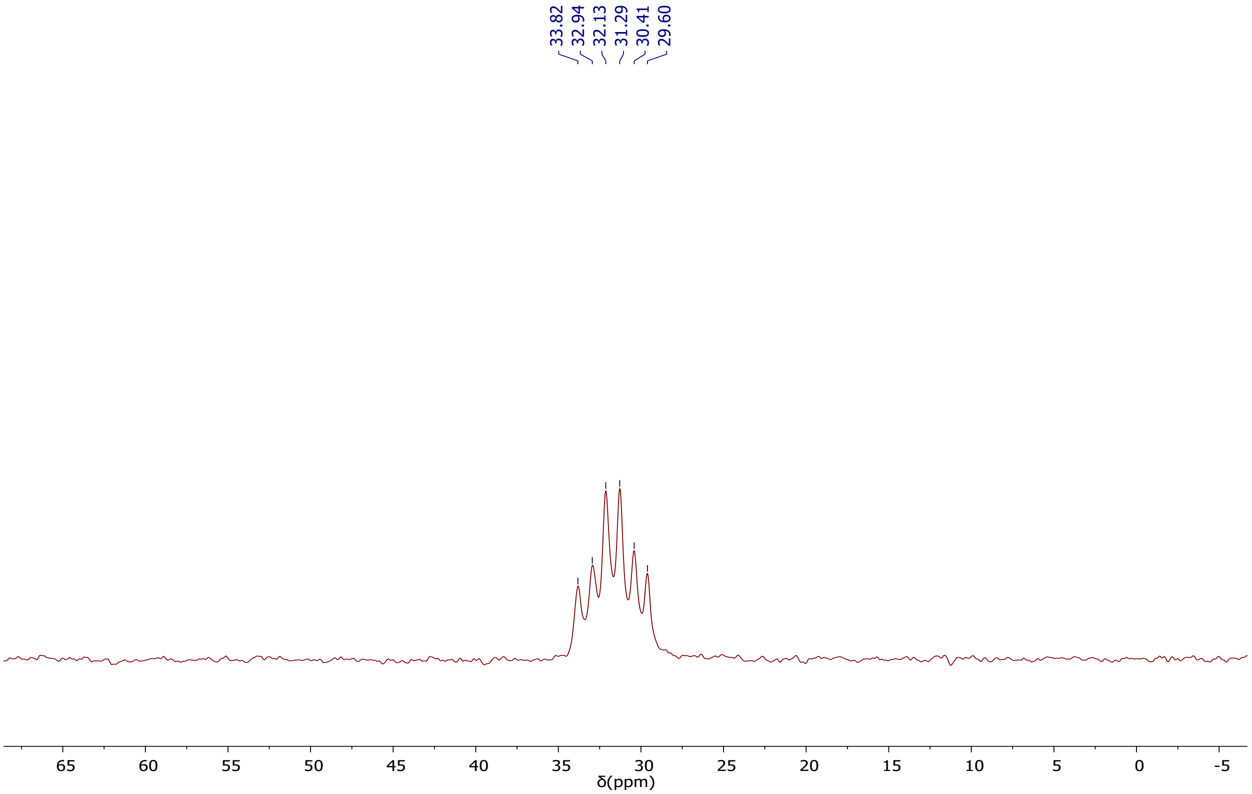


**Figure S31.** Solid-state $^{29}Si$ NMR spectrum of **7**.

**S3. UV-Vis Spectra**

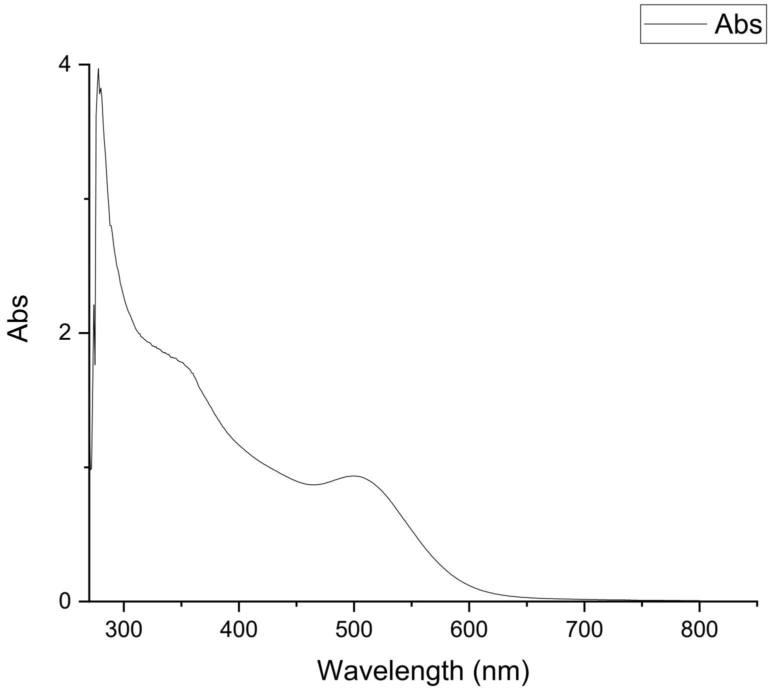


**Figure S32.** UV-vis spectrum of **3**.

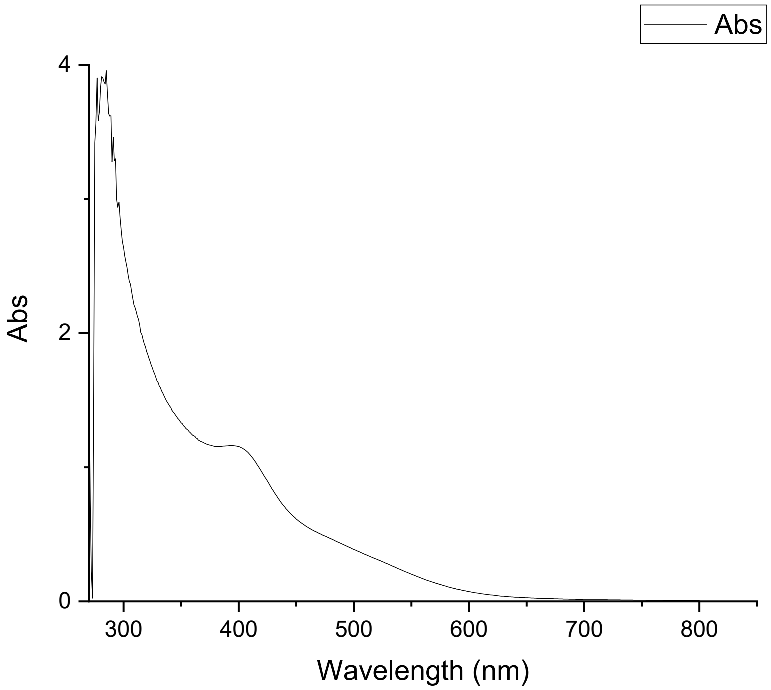


**Figure S33.** UV-vis spectrum of **4**.

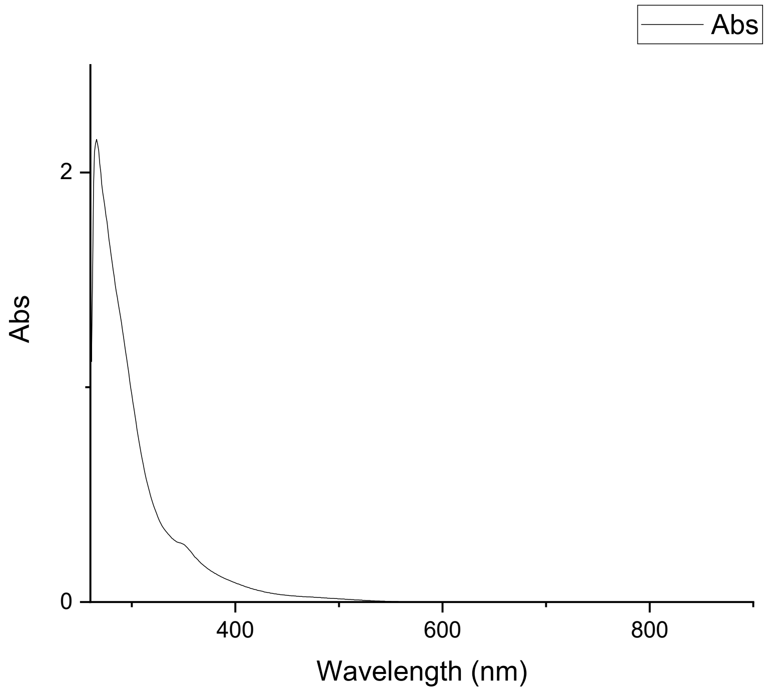


**Figure S34.** UV-vis spectrum of **5**.

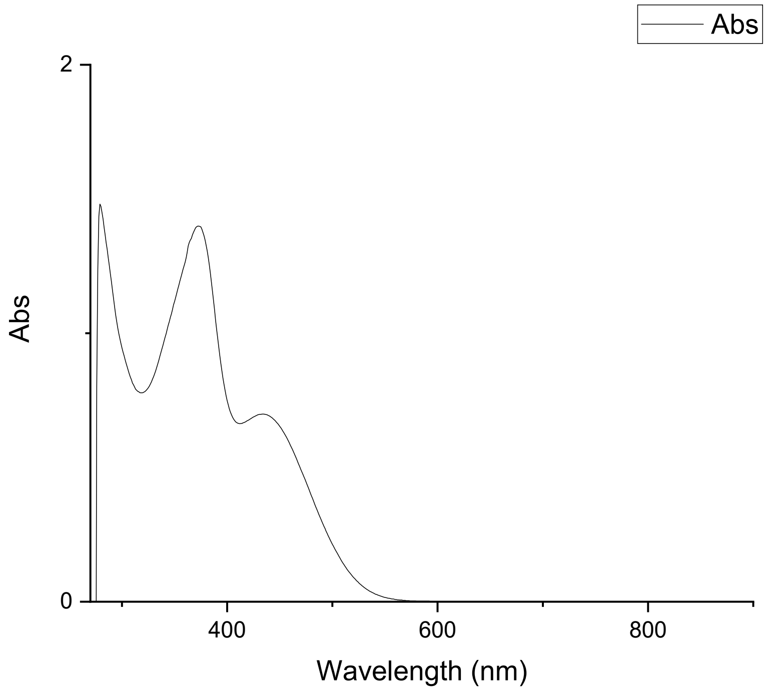


**Figure S35.** UV-vis spectrum of **6**.

## S4. X-ray Data Collection and Structural Refinement

The X-ray diffraction intensity data of all compounds were measured using a Bruker D8 Quest diffractometer equipped with a CCD detector at 100 K and employing Mo K α radiation (λ = 0.71073 Å) with the SMART suite of programs. SAINT was used to correct Lorentz and polarization effects and SADABS was used to correct absorption effects. The SHELXTL suite of programs were employed for solving of structures and structural refinement.[S3,S4] Direct methods were employed for the location of the heavier atoms, ensued by difference maps for the lighter, non-hydrogen atoms for structural solution. Anisotropic thermal parameters were used for the refinement of all non-hydrogen atoms. Deposition numbers 2394597 for **2**, 2394598 for **3**, 2394599 for **4**, 2394600 for **5**, 2394601 for **6**, 2394602 for **7** contain the supplementary crystallographic data for this paper. These data are provided free of charge by the joint Cambridge Crystallographic Data Centre and Fachinformationszentrum Karlsruhe Access Structures service.

**Table S1.** X-Ray crystallographic data for compound **2 - 4**

| | **2** | **3** | **4** |
|---|---|---|---|
| Formula | $C_{33}H_{45}BCl_3N_2PSi$ | $C_{66}H_{90}B_2K_2N_4P_2Si_2$ | $C_{81}H_{111}B_2Cu_2N_4P_3Si_2$ |
| Fw | 645.93 | 1157.35 | 1438.52 |
| Temperature/K | 100(2) | 100(2) | 100(2) |
| crystal system | triclinic | triclinic | monoclinic |
| space group | *P* -1 | *P* -1 | *P* 1 21/*c* 1 |
| *a* (Å) | 9.0296(4) | 8.9473(9) | 15.9089(9) |
| *b* (Å) | 9.2046(4) | 12.2250(11) | 12.3323(6) |
| *c* (Å) | 23.1133(12) | 15.6398(16) | 40.245(3) |
| *α* (deg) | 89.022(2) | 87.641(3) | 90 |
| *β* (deg) | 79.051(2) | 89.746(4) | 100.860(2) |
| *γ* (deg) | 63.4122(17) | 71.362(3) | 90 |
| *V* (Å$^3$) | 1681.56(14) | 1619.5(3) | 7754.4(8) |
| *Z* | 2 | 1 | 4 |
| $d_{calcd}$ (g cm$^{-3}$) | 1.276 | 1.187 | 1.232 |
| *μ* (mm$^{-1}$) | 0.381 | 0.275 | 0.686 |
| *F* (000) | 684 | 620 | 3064 |
| crystal size (mm) | 0.160 x 0.220 x 0.240 | 0.160 x 0.200 x 0.220 | 0.120 x 0.140 x 0.160 |
| 2*θ* range (deg) | 5.295 < 2*θ* < 67.31 | 4.804 < 2*θ* < 57.28 | 4.447 < 2*θ* < 61.92 |
| index range | -13 ≤ *h* ≤13,<br>-13 ≤ *k* ≤13,<br>-33 ≤ *l* ≤33 | -12 ≤ *h* ≤ 12,<br>-15 ≤ k ≤ 16,<br>-21 ≤ *l* ≤ 21 | -23 ≤ *h* ≤ 23,<br>-17 ≤ *k* ≤ 15,<br>-58 ≤ *l* ≤ 58 |
| no. of reflections collected | 34931 | 35840 | 166025 |
| no. of independent reflections | 10481 | 8351 | 24733 |
| *R1, wR2 (I > 2σ(I))* | 0.0517/0.1281 | 0.0795/0.1979 | 0.0537/0.1142 |
| *R1, wR2* (all data) | 0.0772/0.1445 | 0.1355/0.2432 | 0.1009/0.1363 |
| goodness of fit, *F*$^2$ | 1.036 | 1.023 | 1.030 |
| no. of data/restraints/parameters | 10481 / 0 / 380 | 8351 / 315 / 441 | 24733 / 0 / 846 |
| largest diff peak and hole, eÅ$^{-3}$ | 0.763 and -0.440 | 0.784 and -0.501 | 0.778 and -0.612 |

**Table S2.** X-Ray crystallographic data for compound **5** - **7**

| | **5** | **6** | **7** |
|---|---|---|---|
| Formula | $C_{47}H_{61}BIrN_2PSi$ | $C_{36}H_{54}BN_2PSi_2$ | $C_{43}H_{59}BF_3N_2O_3PSSi$ |
| Fw | 916.04 | 612.77 | 810.85 |
| Temperature/K | 100(2) | 101(2) | 100(2) |
| crystal system | triclinic | monoclinic | monoclinic |
| space group | *P* -1 | *P* 1 21/*c* 1 | *P* 1 21/*n* 1 |
| *a* (Å) | 15.2155(19) | 11.8487(6) | 16.6108(7) |
| *b* (Å) | 15.695(2) | 10.1385(7) | 10.1947(4) |
| *c* (Å) | 18.597(2) | 30.475(2) | 25.8173(13) |
| $\alpha$ (deg) | 90.735(4) | 90 | 90 |
| $\beta$ (deg) | 105.707(4) | 94.814(4) | 90.0710(15) |
| $\gamma$ (deg) | 98.278(4) | 90 | 90 |
| $V$ (Å$^3$) | 4224.5(9) | 3648.0(4) | 4372.0(3) |
| $Z$ | 4 | 4 | 4 |
| $d_{calcd}$ (g cm$^{-3}$) | 1.440 | 1.116 | 1.232 |
| $\mu$ (mm$^{-1}$) | 3.261 | 1.478 | 0.190 |
| $F$ (000) | 1872 | 1328 | 1728 |
| crystal size (mm) | 0.010 x 0.060 x 0.120 | 0.040 x 0.060 x 0.080 | 0.020 x 0.040 x 0.160 |
| 2$\theta$ range (deg) | 4.541 < 2$\theta$ < 54.14 | 5.820 < 2$\theta$ < 136.2 | 4.688 < 2$\theta$ < 52.66 |
| index range | $-19 \leq h \leq 19$, $-20 \leq k \leq 20$, $-23 \leq l \leq 23$ | $-14 \leq h \leq 14$, $-12 \leq k \leq 12$, $-36 \leq l \leq 36$ | $-20 \leq h \leq 18$, $-12 \leq k \leq 12$, $-32 \leq l \leq 32$ |
| no. of reflections collected | 135351 | 31379 | 43201 |
| no. of independent reflections | 18422 | 6688 | 8940 |
| *R1, wR2* $(I > 2\sigma(I))$ | 0.0486/0.1054 | 0.0918/0.2090 | 0.0606/0.1424 |
| *R1, wR2* (all data) | 0.0780/0.1244 | 0.1430/0.2361 | 0.1132/0.1789 |
| goodness of fit, $F^2$ | 1.044 | 1.060 | 1.020 |
| no. of data/restraints/parameters | 18422 / 0 / 971 | 6688 / 877 / 485 | 8940 / 580 / 593 |
| largest diff peak and hole, eÅ$^{-3}$ | 2.272 and -1.339 | 0.549 and -0.483 | 0.415 and -0.535 |

## S5. Theoretical Studies

Geometry optimizations were carried out using density functional theory at M06-2X level [S5] in conjunction with the def2-TZVP basis set. [S6] The single-point calculations were performed using the Gaussian 16 B.01 program. [S7] The TD-DFT [S8] and NBO [S9] analyses were all carried out at the M06-2X/def2-TZVP level of theory.

**Compound 3**

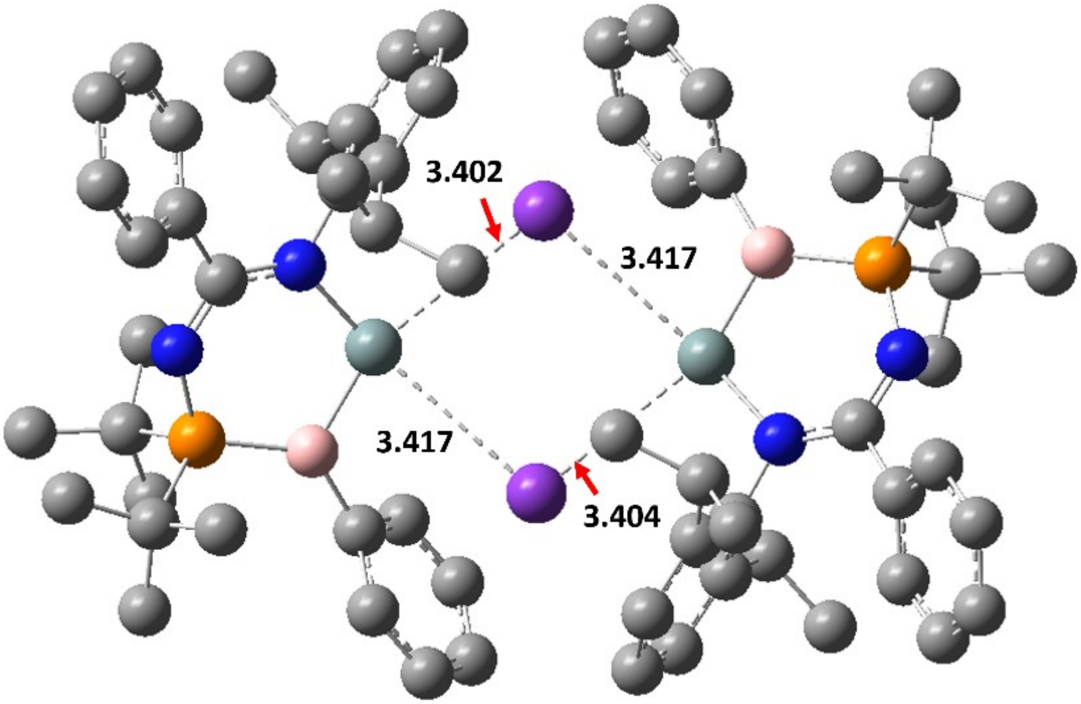


**Figure S36.** Optimized geometries of compound **3** at M06-2X/def2-TZVP level of theory. (Grey: C, Blue: N, Pink: B, Green: Si, Purple: K, Orange: P). Hydrogen atoms are omitted for clarity. The bond lengths displayed are measured in Angstroms (Å).

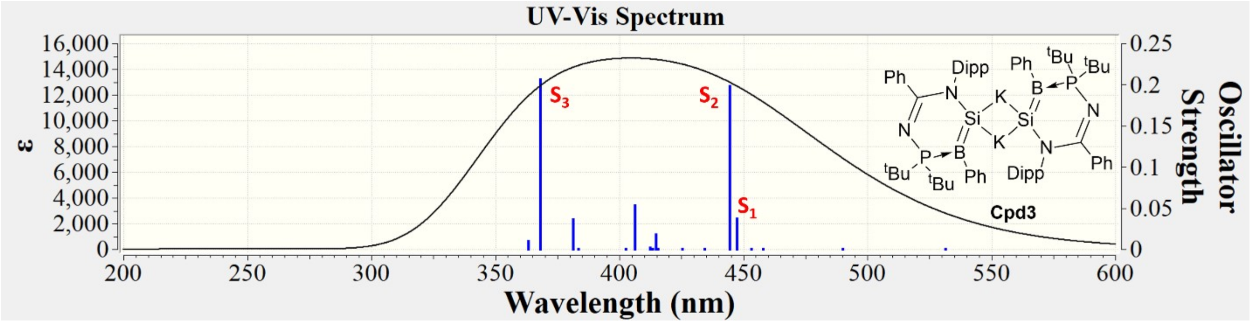


| State | l (nm) | $f_{calc}$ | nature | | | contribution |
|---|---|---|---|---|---|---|
| $S_1$ | 447.61 | 0.0367 | HOMO-1 | → | LUMO+1 | 31% |
| □ | □ | □ | HOMO-1 | → | LUMO+4 | 18% |
| | | | HOMO-1 | → | LUMO+6 | 11% |
| | | | HOMO | → | LUMO | 50% |
| | | | HOMO | → | LUMO+9 | 11% |
| $S_2$ | 444.47 | 0.1976 | HOMO-1 | → | LUMO | 47% |
| | | | HOMO-1 | → | LUMO+9 | 12% |
| | | | HOMO | → | LUMO+1 | 31% |
| | | | HOMO | → | LUMO+4 | 21% |
| | | | HOMO | → | LUMO+6 | 11% |
| $S_3$ | 368.28 | 0.2059 | HOMO | → | LUMO+1 | 20% |
| | | | HOMO | → | LUMO+2 | 22% |
| | | | HOMO | → | LUMO+4 | 46% |

**Figure S37.** UV-Vis spectrum and absorption band of compound **3** ($f_{calc}$ = oscillator strength). Details of molecular orbitals were found in Figure S38.

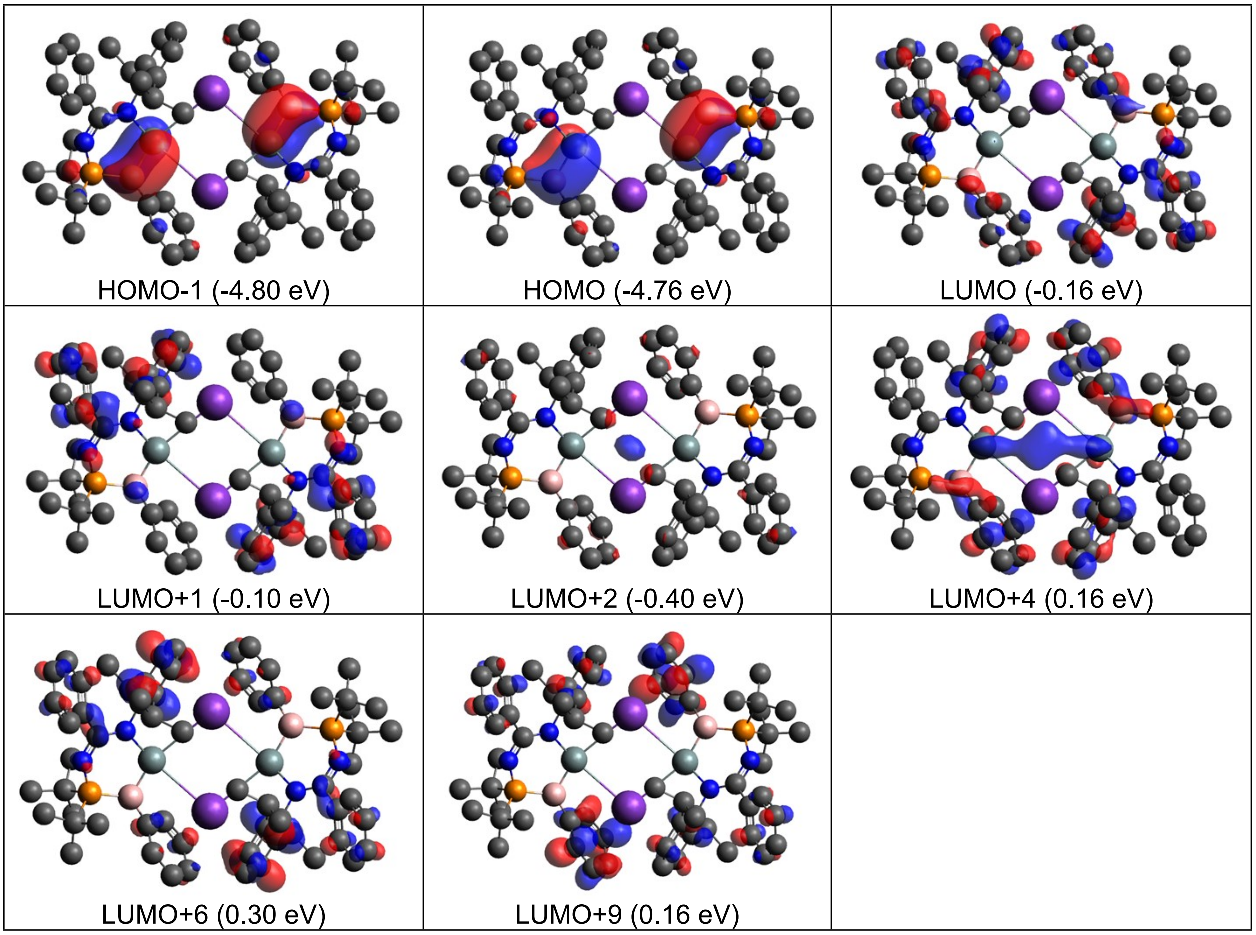


**Figure S38.** Molecular orbitals of compound **3**.

| Bond type | Occupancy | Polarization | Hybridization | WBI | NPA |
|---|---|---|---|---|---|
| $Si_1$ (Lone Pair) | 1.84 | 100.00 % $Si_1$ | Si: $sp^{0.56}$ | - | $Si_1$: +0.34<br>$Si_2$: +0.34<br>$B_1$: -0.79<br>$B_2$: -0.79<br>$K_1$: +0.86<br>$K_2$: +0.86 |
| $Si_2$ (Lone Pair) | 1.84 | 100.00 % $Si_1$ | Si: $sp^{0.56}$ | - | |
| $Si_1$-$B_1$ (σ Bond) | 1.90 | 37.39 % $Si_1$ + 62.61 % $B_1$ | Si: $sp^{2.42}$<br>B: $sp^{1.79}$ | 1.586 | |
| $Si_1$-$B_1$ (π Bond) | 1.75 | 43.63 % $Si_1$ + 56.37 % $B_1$ | Si: $sp^{99.99}$<br>B: $sp^{87.86}$ | | |
| $Si_2$-$B_2$ (σ Bond) | 1.90 | 37.39 % $Si_2$ + 62.61 % $B_2$ | Si: $sp^{2.42}$<br>B: $sp^{1.79}$ | 1.586 | |
| $Si_2$-$B_2$ (π Bond) | 1.75 | 43.61 % $Si_2$ + 56.39 % $B_2$ | Si: $sp^{99.99}$<br>B: $sp^{87.82}$ | | |
| $K_1$ (Lone Vacancy) | 0.12 | 100.00 % $K_1$ | K: s | - | |
| $K_2$ (Lone Vacancy) | 0.12 | 100.00 % $K_2$ | K: s | - | |
| $Si_1$ (Lone Vacancy) | 0.27 | 100.00 % $Si_1$ | Si: $sp^{12.73}$ | - | |
| $Si_2$ (Lone Vacancy) | 0.27 | 100.00 % $Si_2$ | Si: $sp^{12.74}$ | - | |

**Figure S39**. Natural bond orbital (NBO) analysis of compound **3** at M06-2X/Def2-TZVP level of theory.

**Compound 4**

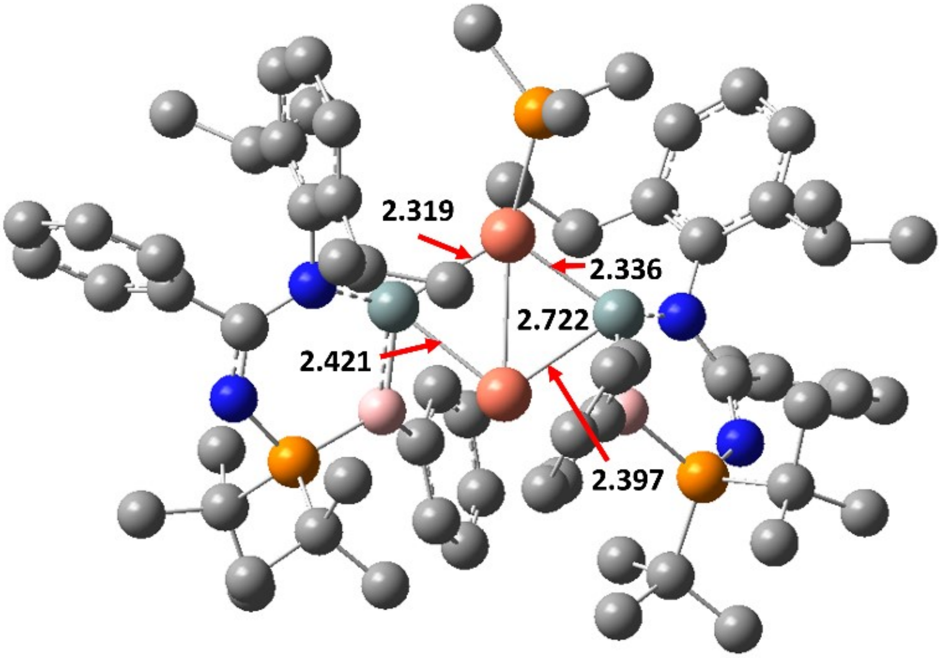


**Figure S40.** Optimized geometries of compound **4** at M06-2X/def2-TZVP level of theory. (Grey: C, Blue: N, Pink: B, Green: Si, Orange: P). Hydrogen atoms are omitted for clarity. The bond lengths displayed are measured in Angstroms (Å).

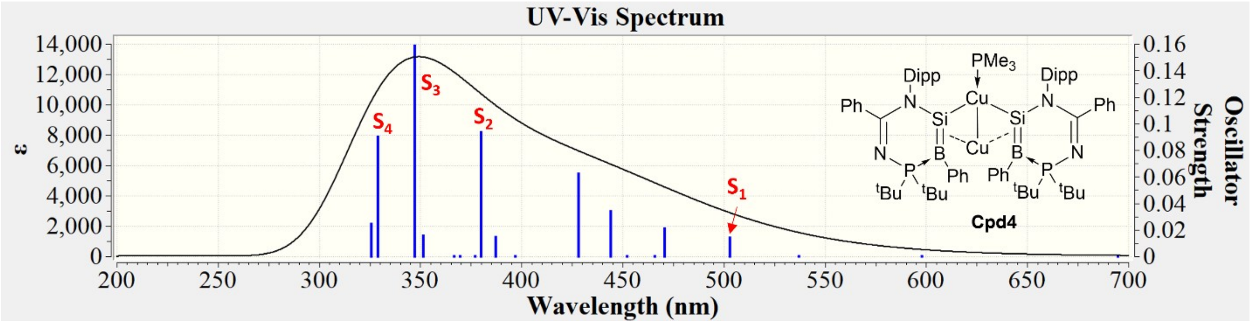


| State | l (nm) | $f_{calc}$ | nature | | | contribution |
|---|---|---|---|---|---|---|
| $S_1$ | 503.15 | 0.0141 | HOMO-2 | → | LUMO | 15% |
| □ | □ | □ | HOMO | → | LUMO | 61% |
| | | | HOMO | → | LUMO+2 | 10% |
| $S_2$ | 380.08 | 0.0932 | HOMO-2 | → | LUMO | 45% |
| | | | HOMO-1 | → | LUMO | 24% |
| | | | HOMO | → | LUMO+1 | 10% |
| $S_3$ | 347.16 | 0.1587 | HOMO-1 | → | LUMO+2 | 16% |
| □ | | | HOMO | → | LUMO+2 | 51% |
| | | | HOMO | → | LUMO+15 | 10% |
| $S_4$ | 329.06 | 0.0897 | HOMO-3 | → | LUMO+3 | 15% |
| | | | HOMO-2 | → | LUMO | 15% |
| | | | HOMO-1 | → | LUMO | 11% |
| | | | HOMO | → | LUMO+3 | 43% |

**Figure S41**. UV-Vis spectrum and absorption band of compound **4** ($f_{calc}$ = oscillator strength). Details of molecular orbitals were found in Figure S42.

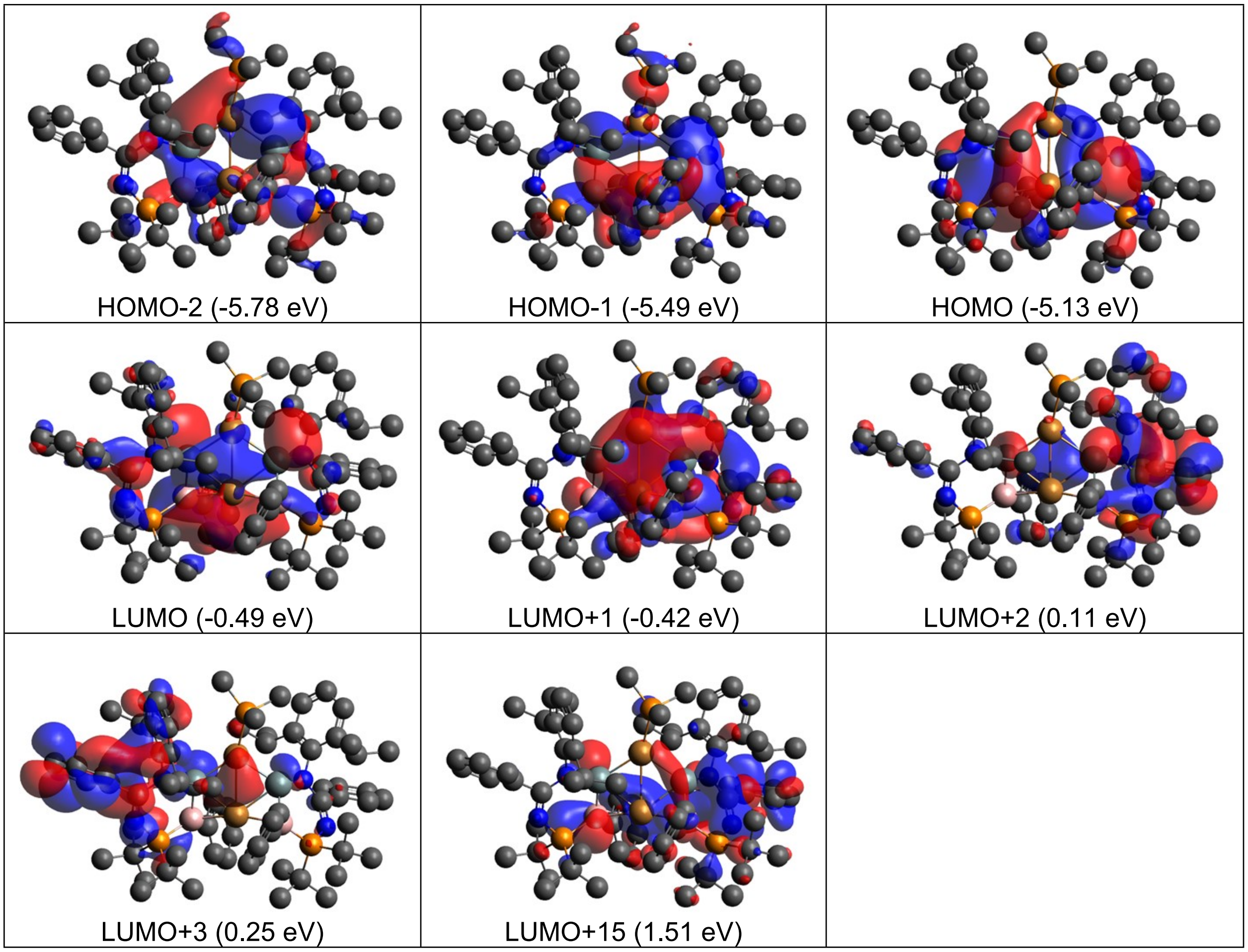


**Figure S42**. Molecular orbitals of compound **4**.

| Bond type | Occupancy | Polarization | Hybridization | WBI | NPA |
|---|---|---|---|---|---|
| $Cu_1$ (Lone Pair) | 1.99 | 100.00 % $Cu_1$ | Cu: $sp^{0.02}d^{99.99}$ | - | $Cu_1$: +0.62<br>$Cu_2$: +0.80<br>$Si_1$: +0.37<br>$Si_2$: +0.38<br>$B_1$: -0.90<br>$B_2$: -0.92 |
| $Cu_1$ (Lone Pair) | 1.99 | 100.00 % $Cu_1$ | Cu: $sp^{0.05}d^{99.99}$ | | |
| $Cu_1$ (Lone Pair) | 1.98 | 100.00 % $Cu_1$ | Cu: $sp^{0.00}d^{1.00}$ | | |
| $Cu_1$ (Lone Pair) | 1.97 | 100.00 % $Cu_1$ | Cu: $sp^{0.02}d^{99.99}$ | | |
| $Cu_1$ (Lone Pair) | 1.97 | 100.00 % $Cu_1$ | Cu: $sp^{0.02}d^{99.99}$ | | |
| $Cu_2$ (Lone Pair) | 1.99 | 100.00 % $Cu_2$ | Cu: $sp^{0.00}d^{1.00}$ | - | |
| $Cu_2$ (Lone Pair) | 1.99 | 100.00 % $Cu_2$ | Cu: $sp^{0.03}d^{99.99}$ | | |
| $Cu_2$ (Lone Pair) | 1.98 | 100.00 % $Cu_2$ | Cu: $sp^{0.01}d^{99.99}$ | | |
| $Cu_2$ (Lone Pair) | 1.97 | 100.00 % $Cu_2$ | Cu: $sp^{0.00}d^{99.99}$ | | |
| $Cu_2$ (Lone Pair) | 1.96 | 100.00 % $Cu_2$ | Cu: $sp^{1.00}d^{99.99}$ | | |
| $Si_1$ (Lone Pair) | 1.70 | 100.00 % $Si_1$ | Si: $sp^{0.68}$ | - | |
| $Si_2$ (Lone Pair) | 1.72 | 100.00 % $Si_2$ | Si: $sp^{0.67}$ | | |
| $Si_1$-$B_1$ (σ bond) | 1.92 | 38.91 % $Si_1$ + 61.09 % $B_1$ | Si: $sp^{1.73}$<br>B: $sp^{2.23}$ | 1.51 | |
| $Si_1$-$B_1$ (π bond) | 1.73 | 40.65 % $Si_1$ + 59.35 % $B_1$ | Si: $sp^{99.99}$<br>B: $sp^{30.09}$ | | |
| $Si_2$-$B_2$ (σ bond) | 1.91 | 39.39 % $Si_1$ + 60.61 % $B_1$ | Si: $sp^{1.79}$<br>B: $sp^{2.26}$ | 1.52 | |
| $Si_2$-$B_2$ (π bond) | 1.75 | 39.05 % $Si_1$ + 60.95 % $B_1$ | Si: $sp^{99.99}$<br>B: $sp^{23.85}$ | 1.52 | |
| $Cu_1$ (Lone Vacancy) | 0.43 | 100.00 % $Cu_1$ | Cu: $sp^{0.00}d^{0.01}$ | - | |
| $Cu_2$ (Lone Vacancy) | 0.26 | 100.00 % $Cu_2$ | Cu: $sp^{0.00}d^{0.01}$ | | |
| $Si_1$ (Lone Vacancy) | 0.34 | 100.00 % $Si_1$ | Si: $sp^{8.91}d^{0.06}$ | | |
| $Si_2$ (Lone Vacancy) | 0.32 | 100.00 % $Si_2$ | Si: $sp^{8.97}d^{0.07}$ | | |

**Figure S43**. Natural bond orbital (NBO) analysis of compound **4** at M06-2X/Def2-TZVP level of theory.

Compound **5**

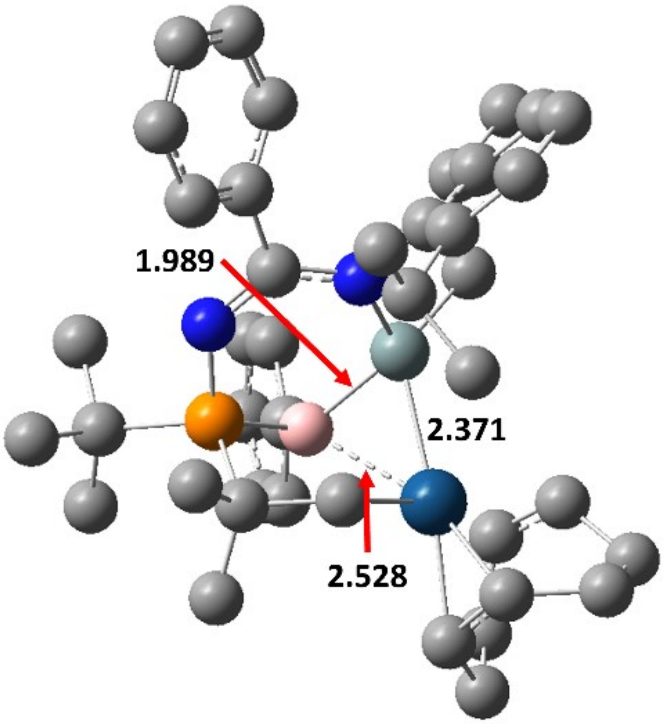


**Figure S44.** Optimized geometries of compound **5** at M06-2X/def2-TZVP level of theory. (Grey: C, Blue: N, Pink: B, Green: Si, Orange: P, Dark blue: Ir). Hydrogen atoms are omitted for clarity. The bond lengths displayed are measured in Angstroms (Å).

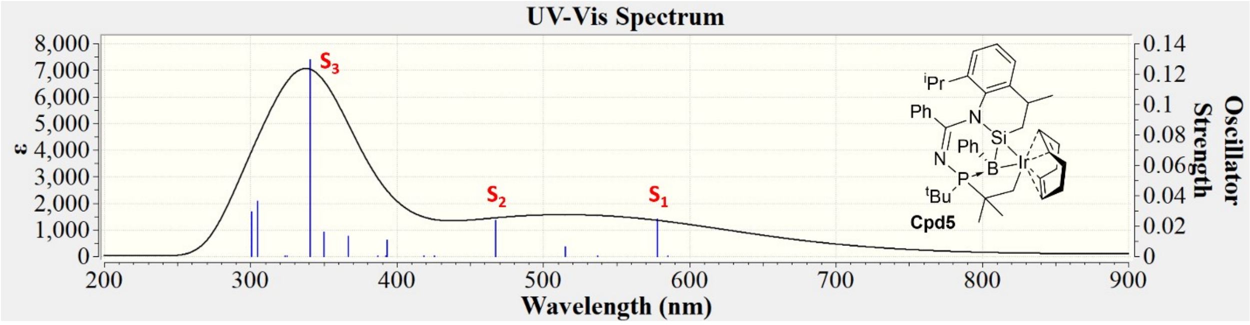


| State | l (nm) | $f_{calc}$ | nature | | | contribution |
|---|---|---|---|---|---|---|
| $S_1$ | 577.89 | 0.0245 | HOMO-4 | → | LUMO | 25% |
| □ | □ | □ | HOMO-1 | → | LUMO | 56% |
| | | | HOMO | → | LUMO | 29% |
| $S_2$ | 467.69 | 0.0233 | HOMO-5 | → | LUMO | 62% |
| | | | HOMO-4 | → | LUMO | 21% |
| | | | HOMO-3 | → | LUMO | 12% |
| $S_3$ | 340.50 | 0.1291 | HOMO-13 | → | LUMO | 15% |
| □ | | | HOMO-12 | → | LUMO | 20% |
| | | | HOMO-10 | → | LUMO | 20% |
| | | | HOMO-6 | → | LUMO | 22% |
| | | | HOMO-3 | → | LUMO | 47% |

**Figure S45.** UV-Vis spectrum and absorption band of compound **5** ($f_{calc}$ = oscillator strength). Details of molecular orbitals were found in Figure S46.

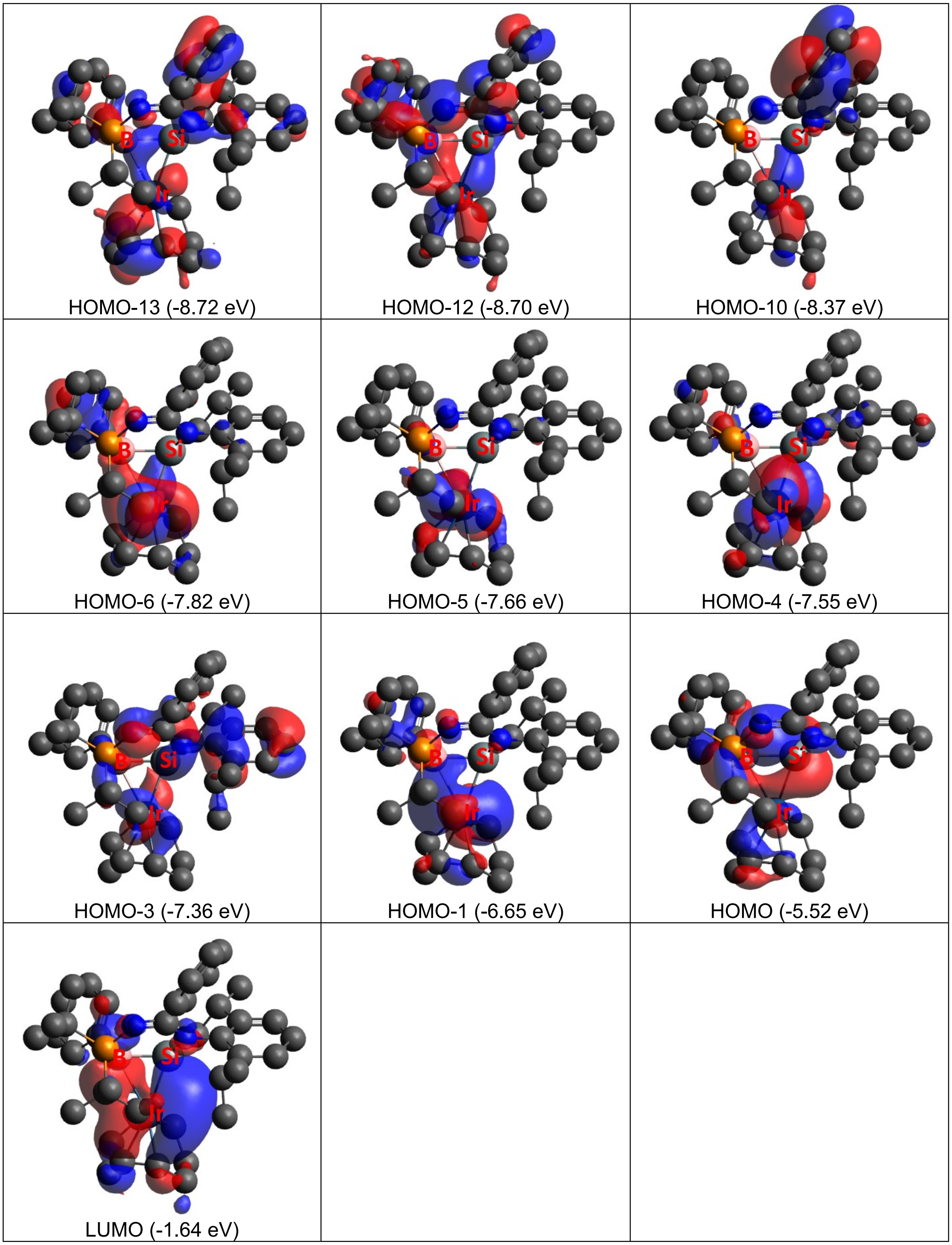


**Figure S46** Molecular orbitals of compound **5** (side view).

| Bond type | Occupancy | Polarization | Hybridization | WBI | NPA |
| --- | --- | --- | --- | --- | --- |
| Ir (Lone Pair) | 1.95 | 100.00 % Ir | Ir: $sp^{0.01}d^{99.99}f^{0.01}$ | - | Si: +1.31<br>B: -0.36<br>Ir: +0.12 |
| Ir (Lone Pair) | 1.90 | 100.00 % Ir | Ir: $sp^{0.03}d^{99.99}f^{0.01}$ | | |
| Ir (Lone Pair) | 1.68 | 100.00 % Ir | Ir: $sp^{1.00}d^{99.99}f^{0.30}$ | | |
| Si (Lone Pair) | 0.88 | 100.00 % Si | Si: $sp^{6.24}d^{0.03}f^{0.00}$ | | |
| Si-B (σ bond) | 1.87 | 40.95 % Si + 59.05 % B | Si: $sp^{2.19}d^{0.01}f^{0.00}$<br>B: $sp^{3.45}d^{0.01}f^{0.00}$ | 1.08 | |
| Si (Lone Vacancy) | 0.34 | 100.00 % Si | Si: $sp^{8.11}d^{0.05}f^{0.00}$ | - | |
| B (Lone Vacancy) | 0.68 | 100.00 % B | B: $sp^{9.50}d^{0.03}f^{0.00}$ | | |

**Figure S47**. Natural bond orbital (NBO) analysis of compound **5** at M06-2X/Def2-TZVP level of theory.

Compound **6**

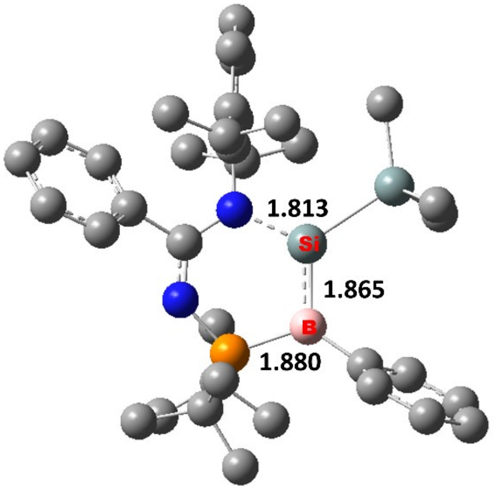


**Figure S48** Optimized geometries of compound **6** at M06-2X/def2-TZVP level of theory. (Grey: C, Blue: N, Pink: B, Green: Si, Orange: P). Hydrogen atoms are omitted for clarity. The bond lengths displayed are measured in Angstroms (Å).

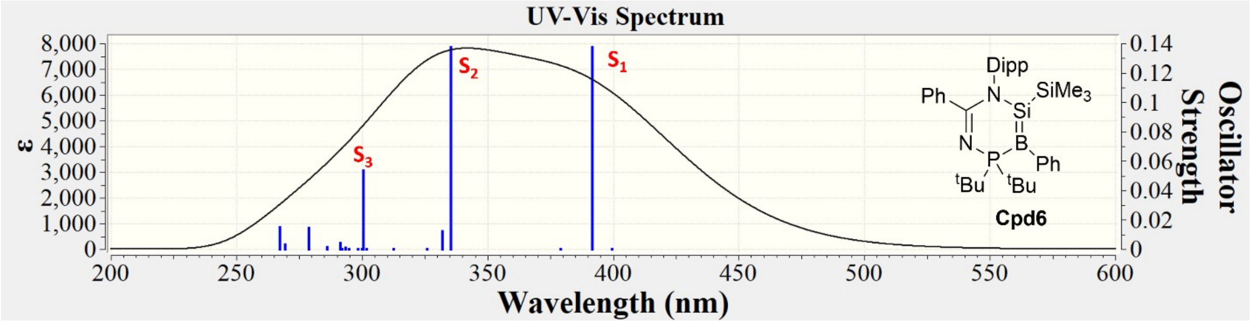


| State | l (nm) | $f_{calc}$ | nature | | | contribution |
|---|---|---|---|---|---|---|
| $S_1$ | 391.70 | 0.1374 | HOMO | → | LUMO | 67% |
| | | | HOMO | → | LUMO+1 | 10% |
| $S_2$ | 335.36 | 0.1371 | HOMO | → | LUMO+1 | 58% |
| | | | HOMO | → | LUMO+5 | 16% |
| | | | HOMO | → | LUMO+8 | 15% |
| $S_3$ | 300.49 | 0.0534 | HOMO | → | LUMO+3 | 36% |
| | | | HOMO | → | LUMO+5 | 11% |
| | | | HOMO | → | LUMO+8 | 28% |

**Figure S49**. UV-Vis spectrum and absorption band of compound **6** ($f_{calc}$ = oscillator strength). Details of molecular orbitals were found in Figure S50.

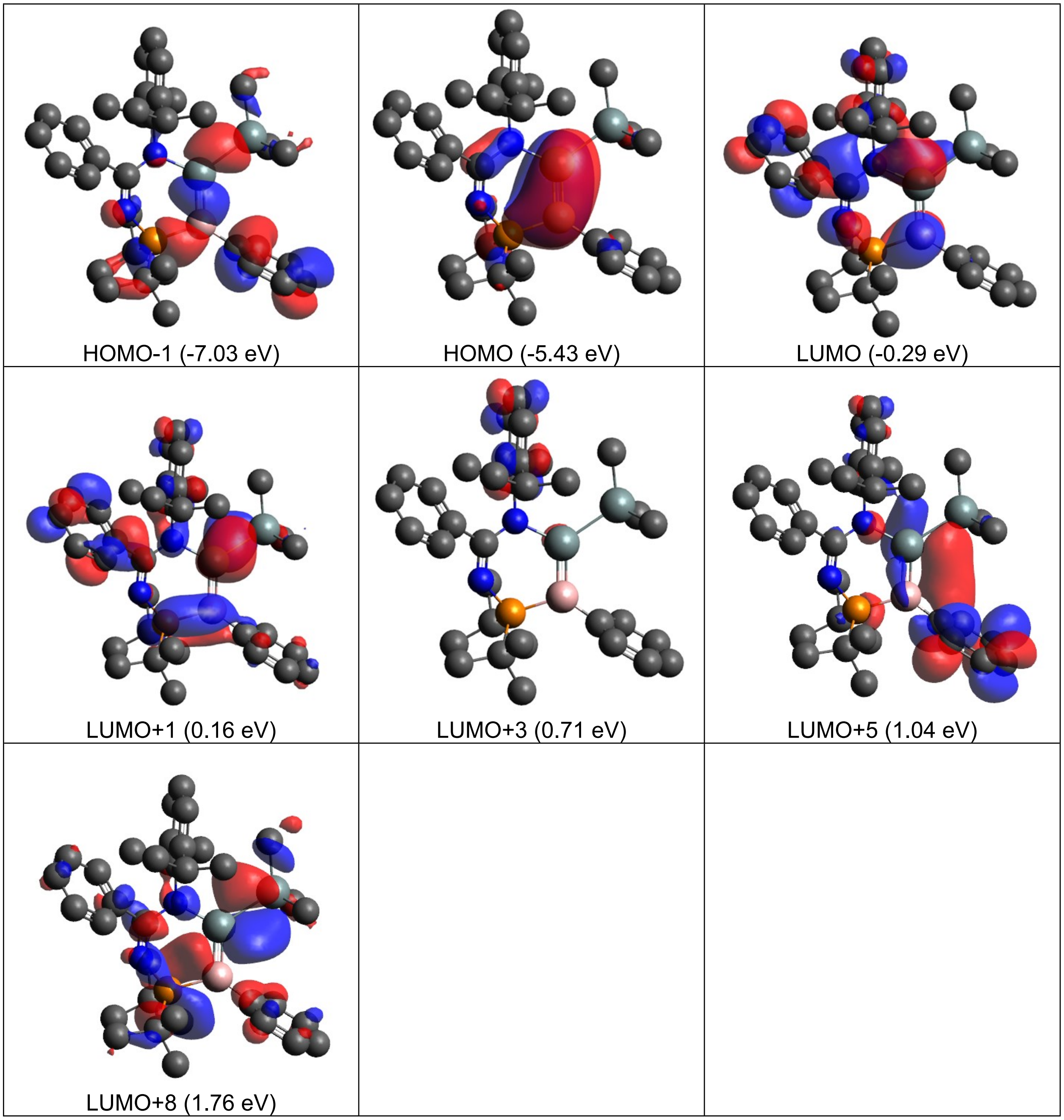


**Figure S50**. Molecular orbitals of compound **6**.

| Bond type | Occupancy | Polarization | Hybridization | WBI | NPA |
|---|---|---|---|---|---|
| Si-B (σ bond) | 1.93 | 42.18 % Si + 57.82 % B | Si: $sp^{1.28}$<br>B: $sp^{1.87}$ | 1.66 | Si: +0.75<br>B: -0.62 |
| Si-B (π bond) | 1.77 | 51.11 % Si + 48.89 % B | Si: $sp^{99.99}$<br>B: $p^{1.00}$ | | |

**Figure S51**. Natural bond orbital (NBO) analysis of compound **6** at M06-2X/Def2-TZVP level of theory.

**Table S3.** Cartesian coordinates and theoretical UV-Vis spectrum for **3**.

**M06-2X/def2-TZVP**

| Atomic Number | Coordinates (Angstroms) X | Y | Z |
|---|---|---|---|
| K | -0.33886300 | 0.56418100 | 0.06990200 |
| P | 0.03040700 | -0.06689000 | 5.38358400 |
| Si | 1.61792000 | 0.87986200 | 2.85413700 |
| N | 2.91543100 | 0.19367000 | 4.05934500 |
| N | 1.52988300 | -0.69792300 | 5.76026300 |
| C | 4.22292600 | 0.29999400 | 3.49194400 |
| C | 4.88961100 | 1.54834300 | 3.53900900 |
| C | 6.12214900 | 1.67955000 | 2.88972300 |
| H | 6.64018400 | 2.64083000 | 2.91818800 |
| C | 6.69702100 | 0.60998800 | 2.20580300 |
| H | 7.65825900 | 0.73041400 | 1.70399300 |
| C | 6.03385000 | -0.61405000 | 2.17043500 |
| H | 6.48355100 | -1.45384700 | 1.63609800 |
| C | 4.79643600 | -0.79121900 | 2.80287300 |
| C | 4.09870200 | -2.13597000 | 2.69096800 |
| H | 3.21282200 | -2.11739800 | 3.33941000 |
| C | 3.59623900 | -2.35613900 | 1.26062300 |
| H | 3.06754900 | -3.31746900 | 1.17534000 |
| H | 4.42847400 | -2.36313500 | 0.53738500 |
| H | 2.88775100 | -1.56184700 | 0.97870400 |
| C | 5.00339700 | -3.28010200 | 3.15686700 |
| H | 4.43580300 | -4.22152900 | 3.19778400 |
| H | 5.41097500 | -3.07992100 | 4.15873800 |
| H | 5.84643400 | -3.43192200 | 2.46505000 |
| C | 4.30912300 | 2.74801500 | 4.27290600 |
| H | 3.35992400 | 2.42987500 | 4.72406500 |
| C | 4.00779500 | 3.89656800 | 3.30424000 |
| H | 3.56443900 | 4.74674400 | 3.84372400 |
| H | 3.28990700 | 3.58148700 | 2.53098600 |
| H | 4.92705900 | 4.25169800 | 2.81081700 |
| C | 5.23250800 | 3.22418700 | 5.39989500 |
| H | 4.76614200 | 4.06002700 | 5.94168500 |
| H | 6.19751500 | 3.57864100 | 5.00575600 |
| H | 5.43648200 | 2.42587900 | 6.12764200 |
| C | 2.68152100 | -0.52826900 | 5.18706300 |
| C | 3.80961300 | -1.28500600 | 5.84893600 |
| C | 5.07757300 | -0.74512900 | 6.09158200 |
| H | 5.30551100 | 0.27565900 | 5.79012300 |
| C | 6.05963800 | -1.50202500 | 6.73092100 |
| H | 7.03973400 | -1.06104400 | 6.91851800 |
| C | 5.79273500 | -2.81026400 | 7.12950500 |
| H | 6.56546200 | -3.40264500 | 7.62190600 |
| C | 4.52736000 | -3.35326400 | 6.90285200 |
| H | 4.30505000 | -4.37431400 | 7.21676500 |
| C | 3.54350800 | -2.59263200 | 6.27818400 |
| H | 2.54676600 | -3.00168900 | 6.10895000 |
| C | -0.33354000 | 0.80674600 | 8.08582500 |
| H | -0.26621500 | 1.65959300 | 8.78083600 |
| H | -1.21712500 | -3.23764400 | 7.10971900 |
| H | 0.38716500 | -2.44875700 | 7.11589300 |
| C | -0.20364700 | 1.32054900 | 6.65149300 |
| C | 1.05127300 | 2.19954000 | 6.55744700 |
| H | 0.92246200 | 3.07577400 | 7.21285100 |
| H | 1.20673600 | 2.55383800 | 5.52675000 |
| H | 1.94728300 | 1.65185400 | 6.88620000 |
| H | 0.47423800 | 0.10162000 | 8.33064400 |
| H | -1.30132800 | 0.31715100 | 8.26199000 |
| C | -1.42188900 | 2.16231000 | 6.25667400 |
| H | -1.53057400 | 2.99573500 | 6.96966100 |
| H | -1.29936000 | 2.58616300 | 5.24924200 |
| C | -1.08426800 | -1.54049200 | 5.78658600 |
| C | -0.69516500 | -2.26711600 | 7.07972500 |

| | | | |
|---|---|---|---|
| H | -0.98281200 | -1.71257200 | 7.97968700 |
| C | -2.55217600 | -1.10715200 | 5.83989200 |
| H | -2.83972700 | -0.49598400 | 4.97126400 |
| H | -2.76863200 | -0.52982500 | 6.75037400 |
| C | -0.86345900 | -2.52179800 | 4.62456700 |
| H | -1.49494600 | -3.41197200 | 4.77517800 |
| H | -1.10149900 | -2.07522300 | 3.64948100 |
| C | -1.34301900 | 1.11331500 | 2.82916900 |
| C | -1.35563700 | 2.43051200 | 2.30493500 |
| H | -0.55983900 | 3.11473000 | 2.60815700 |
| C | -2.33002300 | 2.86963100 | 1.40429800 |
| H | -2.28564800 | 3.88997000 | 1.01701100 |
| C | -3.35645600 | 2.01453800 | 1.00091300 |
| H | -4.12223300 | 2.35516500 | 0.30162400 |
| C | -3.39963900 | 0.72005700 | 1.52919100 |
| H | -4.20146900 | 0.03836400 | 1.23684700 |
| C | -2.41769000 | 0.28652200 | 2.41991900 |
| H | -2.46582500 | -0.74364800 | 2.77970300 |
| B | -0.07884400 | 0.61157400 | 3.63686700 |
| H | 0.18887800 | -2.84332800 | 4.58603300 |
| H | -3.19773800 | -2.00038100 | 5.85557000 |
| H | -2.35663500 | 1.58478400 | 6.27105900 |
| K | 3.98197600 | 1.32317900 | 0.44523000 |
| P | 3.67201400 | 1.61053100 | -4.90505000 |
| Si | 1.94629200 | 1.42992800 | -2.29734500 |
| N | 0.93612600 | 0.56613500 | -3.65373000 |
| N | 2.48673600 | 0.56479100 | -5.44351900 |
| C | -0.30410000 | 0.09492600 | -3.12208900 |
| C | -1.37008000 | 1.01264300 | -2.95895400 |
| C | -2.54605600 | 0.57727500 | -2.33834800 |
| H | -3.37062300 | 1.28126600 | -2.20496900 |
| C | -2.68200200 | -0.73326400 | -1.88421500 |
| H | -3.60572800 | -1.05528300 | -1.40117000 |
| C | -1.62834300 | -1.62771500 | -2.05413100 |
| H | -1.73336300 | -2.65565700 | -1.70009600 |
| C | -0.43082900 | -1.23585800 | -2.66668300 |
| C | 0.70009400 | -2.24339000 | -2.78277900 |
| H | 1.50067200 | -1.79183000 | -3.38332400 |
| C | 1.29411200 | -2.53747200 | -1.40169400 |
| H | 2.13010200 | -3.24884300 | -1.47917500 |
| H | 0.54183600 | -2.97317700 | -0.72354200 |
| H | 1.68580500 | -1.61298500 | -0.94994400 |
| C | 0.24292200 | -3.52358900 | -3.48764700 |
| H | 1.10441700 | -4.17955900 | -3.68174400 |
| H | -0.23982900 | -3.29574800 | -4.44938900 |
| H | -0.47068800 | -4.08909400 | -2.86846400 |
| C | -1.27687300 | 2.45391100 | -3.43703800 |
| H | -0.29137500 | 2.58267200 | -3.90346700 |
| C | -1.37287800 | 3.43414200 | -2.26304200 |
| H | -1.27729100 | 4.47036500 | -2.62015200 |
| H | -0.56592900 | 3.25697300 | -1.53483700 |
| H | -2.34232800 | 3.34276900 | -1.74687100 |
| C | -2.34372700 | 2.77077800 | -4.49097600 |
| H | -2.22267500 | 3.80261000 | -4.85206300 |
| H | -3.35898900 | 2.67924200 | -4.07492200 |
| H | -2.27217900 | 2.10143400 | -5.36014600 |
| C | 1.37238300 | 0.19980300 | -4.88784900 |
| C | 0.56845800 | -0.77527000 | -5.71620900 |
| C | -0.81631400 | -0.69238200 | -5.89936500 |
| H | -1.38567300 | 0.10436600 | -5.42394300 |
| C | -1.48182400 | -1.62056800 | -6.70057800 |
| H | -2.56075700 | -1.53503700 | -6.83777500 |
| C | -0.77634800 | -2.64845000 | -7.32303500 |
| H | -1.30094400 | -3.37780300 | -7.94215900 |
| C | 0.60654800 | -2.73212300 | -7.15616400 |
| H | 1.17012700 | -3.52875400 | -7.64428500 |
| C | 1.27188800 | -1.79731300 | -6.36872600 |
| H | 2.35419700 | -1.84448300 | -6.24302200 |
| C | 3.59493100 | 3.05140700 | -7.37839500 |
| H | 3.20068300 | 3.93869200 | -7.90014600 |
| H | 5.90861800 | -0.51097200 | -7.15988100 |

| | | | |
|---|---|---|---|
| H | 4.12909300 | -0.36581900 | -7.06899300 |
| C | 3.34261500 | 3.20220900 | -5.87768500 |
| C | 1.86003200 | 3.53510900 | -5.66065300 |
| H | 1.63937700 | 4.50487800 | -6.13477300 |
| H | 1.62764200 | 3.61016700 | -4.58718600 |
| H | 1.20918100 | 2.77307700 | -6.11519300 |
| H | 3.08501700 | 2.16377300 | -7.78059900 |
| H | 4.66666100 | 2.98440700 | -7.61171900 |
| C | 4.18957100 | 4.33573400 | -5.28922300 |
| H | 3.96290100 | 5.27120100 | -5.82617500 |
| H | 3.96104700 | 4.48737500 | -4.22412000 |
| C | 5.22572400 | 0.74255100 | -5.54416800 |
| C | 5.07453300 | 0.18261300 | -6.96366200 |
| H | 5.10932000 | 0.96251400 | -7.73223100 |
| C | 6.43634800 | 1.67721000 | -5.46557600 |
| H | 6.51831900 | 2.17451300 | -4.48744800 |
| H | 6.39529300 | 2.45508700 | -6.24172300 |
| C | 5.41681800 | -0.45251700 | -4.59699700 |
| H | 6.32063000 | -1.00967300 | -4.89119700 |
| H | 5.51348400 | -0.14173000 | -3.54775500 |
| C | 4.62661100 | 2.69831100 | -2.12824600 |
| C | 4.18564000 | 3.80760100 | -1.36340100 |
| H | 3.18663300 | 4.20453000 | -1.55788000 |
| C | 4.97032200 | 4.38789800 | -0.36302300 |
| H | 4.57750100 | 5.23126900 | 0.20921200 |
| C | 6.24914300 | 3.89795800 | -0.09436200 |
| H | 6.86710200 | 4.35165900 | 0.68263100 |
| C | 6.73403800 | 2.83042800 | -0.85721100 |
| H | 7.73714800 | 2.43961800 | -0.67242000 |
| C | 5.94065800 | 2.25066700 | -1.84708800 |
| H | 6.34198000 | 1.39477900 | -2.39428300 |
| B | 3.59707400 | 1.93880700 | -3.05855900 |
| H | 4.55307300 | -1.13277500 | -4.65553000 |
| H | 7.35880000 | 1.09677000 | -5.62997200 |
| H | 5.26838300 | 4.14887000 | -5.38233700 |

---------------------------------------------------------------------

```
Excited State   1:      Triplet-?Sym    1.5837 eV  782.88 nm  f=0.0000  <S**2>=2.000
     307 -> 315        0.10759
     309 -> 312        0.27306
     309 -> 313       -0.19849
     309 -> 315        0.32327
     309 -> 319        0.12185
     309 -> 323       -0.10685
     309 -> 325       -0.14028
     310 -> 311        0.30252
     310 -> 320        0.10013
     310 -> 324       -0.12967
     310 -> 326       -0.21408
 This state for optimization and/or second-order correction.
 Total Energy, E(TD-HF/TD-DFT) =  -5299.36534358
 Copying the excited state density for this state as the 1-particle RhoCI density.
 -----------------------------------------------------------------------------------------------
---------Excited state symmetry could not be determined.
 Excited State   2:      Triplet-?Sym    1.7264 eV  718.17 nm  f=0.0000  <S**2>=2.000
     309 -> 311        0.34271
     309 -> 324       -0.14389
     309 -> 326       -0.22971
     310 -> 312        0.31148
     310 -> 313       -0.17771
     310 -> 315        0.29603
     310 -> 319        0.11742
     310 -> 325       -0.10959
     310 -> 329       -0.11009
  ----------------------------------------------------------------------------------------------
----------
 Excited state symmetry could not be determined.
 Excited State   3:      Triplet-?Sym    2.3322 eV  531.62 nm  f=0.0000  <S**2>=2.000
     307 -> 312        0.18841
     307 -> 313       -0.15179
     307 -> 315        0.27770
     307 -> 319        0.11370
     307 -> 323       -0.10129
     307 -> 325       -0.13109
     308 -> 311        0.22317
     308 -> 324       -0.11073
     308 -> 326       -0.15053
     309 -> 312       -0.19043
     309 -> 327       -0.10041
     310 -> 311       -0.19252
  ----------------------------------------------------------------------------------------------
----------
 Excited state symmetry could not be determined.
 Excited State   4:      Triplet-?Sym    2.5299 eV  490.08 nm  f=0.0000  <S**2>=2.000
     307 -> 311        0.27654
     307 -> 324       -0.13231
     307 -> 326       -0.19664
     308 -> 312        0.32824
     308 -> 313       -0.15360
     308 -> 315        0.33085
     308 -> 319        0.14058
     308 -> 323       -0.10687
     308 -> 325       -0.11572
     308 -> 329       -0.11835
  ----------------------------------------------------------------------------------------------
----------
 Excited state symmetry could not be determined.
 Excited State   5:      Triplet-?Sym    2.7069 eV  458.03 nm  f=0.0000  <S**2>=2.000
     307 -> 312        0.16850
     307 -> 315        0.12145
     308 -> 311        0.25985
     308 -> 326       -0.14055
     309 -> 312        0.24238
     309 -> 313        0.16676
     309 -> 315       -0.24796
     310 -> 311        0.13017
     310 -> 314        0.23879
     310 -> 318        0.15563
```

```
    310 -> 320       -0.11859
  ----------------------------------------------------------------------------------------------
----------
 Excited state symmetry could not be determined.
 Excited State   6:      Triplet-?Sym    2.7345 eV  453.41 nm  f=0.0000  <S**2>=2.000
     309 -> 314        0.29506
     309 -> 318        0.18577
     309 -> 320       -0.18570
     310 -> 312        0.32603
     310 -> 313        0.23555
     310 -> 315       -0.26835
     310 -> 321        0.10244
  ----------------------------------------------------------------------------------------------
----------
 Excited state symmetry could not be determined.
 Excited State   7:      Singlet-?Sym    2.7699 eV  447.61 nm  f=0.0367  <S**2>=0.000
     309 -> 312        0.31883
     309 -> 313       -0.20684
     309 -> 315        0.18040
     309 -> 317        0.11907
     310 -> 311        0.50134
     310 -> 316       -0.10505
     310 -> 320        0.11421
  ----------------------------------------------------------------------------------------------
----------
 Excited state symmetry could not be determined.
 Excited State   8:      Singlet-?Sym    2.7895 eV  444.47 nm  f=0.1976  <S**2>=0.000
     309 -> 311        0.47281
     309 -> 320        0.12656
     310 -> 312        0.31816
     310 -> 313       -0.24142
     310 -> 315        0.21013
     310 -> 317        0.11797
  ----------------------------------------------------------------------------------------------
----------
 Excited state symmetry could not be determined.
 Excited State   9:      Triplet-?Sym    2.8535 eV  434.50 nm  f=0.0000  <S**2>=2.000
     308 -> 311       -0.10363
     309 -> 312        0.11269
     309 -> 313        0.19554
     309 -> 317       -0.17426
     309 -> 323       -0.22280
     309 -> 325       -0.26681
     310 -> 311       -0.26775
     310 -> 314        0.13307
     310 -> 316        0.16806
     310 -> 320       -0.16576
     310 -> 322       -0.10209
     310 -> 326       -0.21586

  ----------------------------------------------------------------------------------------------
----------Excited state symmetry could not be determined.
 Excited State  10:      Triplet-?Sym    2.9146 eV  425.38 nm  f=0.0000  <S**2>=2.000
     309 -> 311        0.29307
     309 -> 316       -0.10246
     309 -> 326        0.27270
     309 -> 328        0.11435
     310 -> 313       -0.17410
     310 -> 315       -0.17123
     310 -> 317        0.20305
     310 -> 323        0.22928
     310 -> 325        0.25956
------------------------------------------------------------------------------------------------
-------- Excited state symmetry could not be determined.
 Excited State  11:      Triplet-?Sym    2.9826 eV  415.69 nm  f=0.0000  <S**2>=2.000
     309 -> 319        0.18669
     309 -> 321        0.37683
     310 -> 311       -0.10549
     310 -> 314       -0.16455
     310 -> 316       -0.30217
     310 -> 318        0.10925
```

```
     310 -> 320       -0.20505
     310 -> 322        0.11961
     310 -> 324       -0.18556
------------------------------------------------------------------------------------------------
-------- Excited state symmetry could not be determined.
 Excited State  12:      Singlet-?Sym    2.9895 eV  414.73 nm  f=0.0177  <S**2>=0.000
     307 -> 311        0.17356
     308 -> 312        0.20642
     308 -> 315        0.17279
     309 -> 314        0.22448
     309 -> 318        0.15032
     309 -> 320       -0.18778
     310 -> 312        0.33448
     310 -> 313        0.20885
     310 -> 315       -0.21761
     310 -> 321        0.13907
------------------------------------------------------------------------------------------------
-------- Excited state symmetry could not be determined.
 Excited State  13:      Triplet-?Sym    2.9986 eV  413.48 nm  f=0.0000  <S**2>=2.000
     309 -> 314       -0.15907
     309 -> 316       -0.32067
     309 -> 318        0.12211
     309 -> 320       -0.19956
     309 -> 322        0.13006
     309 -> 324       -0.17950
     310 -> 319        0.19625
     310 -> 321        0.38664
------------------------------------------------------------------------------------------------
-------- Excited state symmetry could not be determined.
 Excited State  14:      Singlet-?Sym    3.0061 eV  412.44 nm  f=0.0014  <S**2>=0.000
     307 -> 312        0.13156
     308 -> 311        0.20511
     309 -> 312        0.31900
     309 -> 313        0.23434
     309 -> 315       -0.24396
     309 -> 321        0.12760
     310 -> 314        0.26054
     310 -> 318        0.16010
     310 -> 320       -0.20183
------------------------------------------------------------------------------------------------
-------- Excited state symmetry could not be determined.
 Excited State  15:      Singlet-?Sym    3.0507 eV  406.41 nm  f=0.0531  <S**2>=0.000
     307 -> 311        0.25560
     307 -> 326       -0.12053
     308 -> 312        0.32770
     308 -> 313       -0.13707
     308 -> 315        0.25014
     309 -> 314       -0.19990
     309 -> 316       -0.12925
     310 -> 312       -0.22044
     310 -> 313       -0.15106
     310 -> 315        0.14433
------------------------------------------------------------------------------------------------
-------- Excited state symmetry could not be determined.
 Excited State  16:      Singlet-?Sym    3.0800 eV  402.54 nm  f=0.0002  <S**2>=0.000
     307 -> 312        0.25005
     307 -> 313       -0.10674
     307 -> 315        0.17266
     308 -> 311        0.42541
     308 -> 324       -0.10967
     308 -> 326       -0.13396
     309 -> 312       -0.20954
     309 -> 313       -0.10898
     310 -> 314       -0.18249
     310 -> 316       -0.14181
------------------------------------------------------------------------------------------------
-------- Excited state symmetry could not be determined.
 Excited State  17:      Singlet-?Sym    3.2332 eV  383.47 nm  f=0.0004  <S**2>=0.000
     308 -> 311       -0.15661
     309 -> 319        0.12814
     309 -> 321        0.36500
```

```
     310 -> 314       -0.18719
     310 -> 316       -0.34682
     310 -> 318        0.17991
     310 -> 320       -0.22481
     310 -> 322        0.11465
     310 -> 324       -0.12622
-----------------------------------------------------------------------------------------------
-------- Excited state symmetry could not be determined.
 Excited State  18:      Singlet-?Sym    3.2500 eV  381.49 nm  f=0.0357  <S**2>=0.000
     308 -> 312       -0.14411
     309 -> 314       -0.18330
     309 -> 316       -0.32534
     309 -> 318        0.16980
     309 -> 320       -0.21580
     309 -> 322        0.11794
     309 -> 324       -0.12807
     310 -> 319        0.13534
     310 -> 321        0.38202
     310 -> 323        0.11169
-----------------------------------------------------------------------------------------------
-------- Excited state symmetry could not be determined.
 Excited State  19:      Singlet-?Sym    3.3665 eV  368.28 nm  f=0.2059  <S**2>=0.000
     309 -> 311       -0.16894
     309 -> 316       -0.10180
     309 -> 318       -0.11635
     309 -> 326       -0.14853
     310 -> 312        0.20312
     310 -> 313        0.22343
     310 -> 315        0.46018
     310 -> 317       -0.23166
     310 -> 323       -0.13261
     310 -> 325       -0.13127
-----------------------------------------------------------------------------------------------
-------- Excited state symmetry could not be determined.
 Excited State  20:      Singlet-?Sym    3.4122 eV  363.35 nm  f=0.0098  <S**2>=0.000
     309 -> 312        0.21174
     309 -> 313        0.22844
     309 -> 315        0.47084
     309 -> 317       -0.19992
     309 -> 323       -0.11071
     309 -> 325       -0.10096
     310 -> 311       -0.16511
     310 -> 316       -0.13743
     310 -> 318       -0.12373
     310 -> 326       -0.14433
--------------------------------------------------------------------
```

**Table S4.** Cartesian coordinates and theoretical UV-Vis spectrum for **4**.

**M06-2X/def2-TZVP**

| Atomic Number | Coordinates (Angstroms) X | Y | Z |
|---|---|---|---|
| C | -3.90808300 | 1.74088000 | 0.54458300 |
| C | -3.91979700 | 1.91772200 | 1.94205100 |
| C | -4.27786200 | 3.16236700 | 2.44782400 |
| H | -4.26227900 | 3.30657500 | 3.38668000 |
| C | -4.65717600 | 4.19702200 | 1.60503700 |
| H | -4.91214000 | 5.03789900 | 1.96560700 |
| C | -4.66155000 | 3.99513600 | 0.23208200 |
| H | -4.93703700 | 4.70241500 | -0.33924300 |
| C | -4.27245000 | 2.78274500 | -0.32870000 |
| C | -4.26500100 | 2.63980100 | -1.84097600 |
| H | -4.11853800 | 1.67528100 | -2.06049700 |
| C | -5.59265800 | 3.07618100 | -2.47561700 |
| H | -5.57550900 | 2.88104800 | -3.43620500 |
| H | -5.71862200 | 4.03901200 | -2.34082800 |
| H | -6.33163600 | 2.58835600 | -2.05651900 |
| C | -3.11016100 | 3.44753600 | -2.45121100 |
| H | -3.10026800 | 3.31998100 | -3.42298100 |
| H | -2.26027900 | 3.13975400 | -2.07271800 |
| H | -3.23325700 | 4.39826300 | -2.24783300 |
| C | -3.62593200 | 0.75253600 | 2.88257900 |
| H | -3.86995900 | -0.08778900 | 2.39865600 |
| C | -4.44391500 | 0.78395000 | 4.17870400 |
| H | -4.30776600 | -0.05199100 | 4.67149500 |
| H | -5.39456100 | 0.88485000 | 3.96273100 |
| H | -4.15261600 | 1.53967300 | 4.73080400 |
| C | -2.14425600 | 0.66552000 | 3.21798200 |
| H | -1.97059500 | -0.15914600 | 3.71889800 |
| H | -1.88630600 | 1.43838900 | 3.76267700 |
| H | -1.62110800 | 0.66042300 | 2.38950000 |
| C | -4.56975700 | -0.42815500 | -0.26614600 |
| C | -5.99729100 | 0.06395000 | -0.21416700 |
| C | -6.62662400 | 0.51992500 | 0.93176800 |
| H | -6.13883100 | 0.58285900 | 1.74471800 |
| C | -7.96813200 | 0.88472900 | 0.90099000 |
| H | -8.39488300 | 1.18477700 | 1.69497400 |
| C | -8.68072600 | 0.81575200 | -0.27315600 |
| H | -9.59042400 | 1.08794400 | -0.29442400 |
| C | -8.07002800 | 0.34746100 | -1.42221400 |
| H | -8.56132100 | 0.29481100 | -2.23359300 |
| C | -6.73625000 | -0.04665000 | -1.38994300 |
| H | -6.32685300 | -0.39285600 | -2.17399700 |
| C | -3.76275300 | -3.47138300 | -2.55992900 |
| C | -5.27378900 | -3.78885700 | -2.46466100 |
| H | -5.58411400 | -4.15958100 | -3.31700400 |
| H | -5.76852500 | -2.96688500 | -2.26599900 |
| H | -5.42551300 | -4.44237500 | -1.74992200 |
| C | -3.57019000 | -2.47955300 | -3.71556000 |
| H | -3.93446800 | -2.86417400 | -4.53978200 |
| H | -2.61439800 | -2.29845800 | -3.83382300 |
| H | -4.03828100 | -1.64381400 | -3.50961700 |
| C | -3.00770600 | -4.77046800 | -2.86932700 |
| H | -3.37589100 | -5.17634100 | -3.68187900 |
| H | -3.10715800 | -5.39250400 | -2.11910600 |
| H | -2.05789600 | -4.57117500 | -3.00533600 |
| C | -3.02214000 | -3.74138300 | 0.45674400 |
| C | -3.02228100 | -2.84467400 | 1.70374700 |
| H | -2.83705800 | -3.38823000 | 2.49738000 |
| H | -3.89893100 | -2.41653700 | 1.79812400 |
| H | -2.33118300 | -2.15575200 | 1.61072100 |
| C | -1.69263000 | -4.50728500 | 0.40842200 |
| H | -1.59340700 | -5.04134400 | 1.22412900 |
| H | -0.95086100 | -3.87025500 | 0.34275000 |

| | | | |
|---|---|---|---|
| H | -1.68489000 | -5.09934400 | -0.37258400 |
| C | -4.19700500 | -4.72351400 | 0.57994600 |
| H | -4.13046500 | -5.20753000 | 1.42923600 |
| H | -4.16889300 | -5.36137700 | -0.16322400 |
| H | -5.04176600 | -4.22686000 | 0.55221200 |
| C | -0.47053600 | -1.92218200 | -2.36492700 |
| C | 0.01829500 | -0.97097600 | -3.28346200 |
| H | -0.29064200 | -0.07433700 | -3.22527500 |
| C | 0.93644500 | -1.30237400 | -4.27130400 |
| H | 1.25350400 | -0.63015100 | -4.86287400 |
| C | 1.39486700 | -2.60190500 | -4.40332100 |
| H | 2.01305100 | -2.83251200 | -5.08703100 |
| C | 0.93310300 | -3.55547100 | -3.51811200 |
| H | 1.23264800 | -4.45329100 | -3.59889100 |
| C | 0.04162200 | -3.22280700 | -2.51655200 |
| H | -0.23547500 | -3.89768600 | -1.90804500 |
| C | 3.65092500 | 2.09664400 | -0.89898200 |
| C | 3.08277900 | 2.67524900 | -2.04831400 |
| C | 3.42599700 | 3.99574800 | -2.36938500 |
| H | 3.05490700 | 4.39036900 | -3.14985000 |
| C | 4.28515400 | 4.74024400 | -1.58917800 |
| H | 4.48567300 | 5.64068900 | -1.81587100 |
| C | 4.84986600 | 4.15491800 | -0.47140400 |
| H | 5.45112500 | 4.65946200 | 0.06389300 |
| C | 4.55643300 | 2.84127500 | -0.11305400 |
| C | 5.20316600 | 2.29457600 | 1.15131600 |
| H | 5.04687100 | 1.30721500 | 1.17256800 |
| C | 6.71979300 | 2.53071800 | 1.19073000 |
| H | 7.10788200 | 2.03711400 | 1.94296500 |
| H | 6.89847000 | 3.48812400 | 1.30137700 |
| H | 7.12080800 | 2.21831600 | 0.35303200 |
| C | 4.53411400 | 2.90265700 | 2.39093400 |
| H | 4.96223500 | 2.54914000 | 3.19912300 |
| H | 3.58273900 | 2.66807900 | 2.39653700 |
| H | 4.63096600 | 3.87704200 | 2.36969000 |
| C | 2.09173200 | 1.97882600 | -2.96220400 |
| H | 2.01486800 | 1.02186700 | -2.68224600 |
| C | 0.71171400 | 2.63541100 | -2.85393500 |
| H | 0.09107000 | 2.19437000 | -3.47113800 |
| H | 0.78275700 | 3.58498200 | -3.08532900 |
| H | 0.37862500 | 2.54650800 | -1.93678500 |
| C | 2.54053500 | 2.02893700 | -4.43466500 |
| H | 1.91582500 | 1.51091000 | -4.98406200 |
| H | 3.43974600 | 1.64760800 | -4.51507700 |
| H | 2.55138100 | 2.95935900 | -4.74141200 |
| C | 4.28749600 | -0.20758300 | -0.50170400 |
| C | 5.36218100 | -0.06485100 | -1.53601700 |
| C | 5.00877500 | 0.00808900 | -2.87921000 |
| H | 4.09314500 | 0.07077400 | -3.12453900 |
| C | 5.98649400 | -0.00983600 | -3.86132700 |
| H | 5.73861900 | 0.03874100 | -4.77705300 |
| C | 7.32341500 | -0.09963900 | -3.51246600 |
| H | 7.99383700 | -0.10695700 | -4.18554800 |
| C | 7.67578200 | -0.17825200 | -2.17690900 |
| H | 8.59287100 | -0.23019000 | -1.93377100 |
| C | 6.70503400 | -0.18356700 | -1.19357000 |
| H | 6.95548300 | -0.26773300 | -0.28097200 |
| C | 3.31063700 | -3.70408900 | 0.64952000 |
| C | 2.87526800 | -3.51385100 | -0.80692300 |
| H | 2.66215500 | -4.38607400 | -1.20023900 |
| H | 3.60301500 | -3.09642000 | -1.31320500 |
| H | 2.08285500 | -2.93872900 | -0.83847700 |
| C | 4.64287800 | -4.45839200 | 0.64777500 |
| H | 4.54217900 | -5.29791300 | 0.15237500 |
| H | 4.90889200 | -4.65340900 | 1.57052200 |
| H | 5.33002600 | -3.90653200 | 0.21925100 |
| C | 2.23299300 | -4.52754100 | 1.36635300 |
| H | 2.25588100 | -5.45099800 | 1.03903400 |
| H | 1.35132000 | -4.13898100 | 1.18580400 |
| H | 2.40271800 | -4.51834900 | 2.33114000 |
| C | 4.52348700 | -1.97334900 | 2.89438400 |

| | | | |
|---|---|---|---|
| C | 4.44101500 | -0.57028000 | 3.50246800 |
| H | 4.99174100 | -0.53295100 | 4.31238600 |
| H | 3.50992700 | -0.36795400 | 3.73092900 |
| H | 4.76891400 | 0.08634000 | 2.85360000 |
| C | 4.01646800 | -2.98464800 | 3.92709500 |
| H | 4.52341500 | -2.88349800 | 4.75963300 |
| H | 4.13574400 | -3.89335400 | 3.58028400 |
| H | 3.06568600 | -2.82386700 | 4.10214800 |
| C | 6.00552100 | -2.24355000 | 2.58423300 |
| H | 6.54466500 | -2.06280000 | 3.38285100 |
| H | 6.29832400 | -1.66034500 | 1.85366200 |
| H | 6.11841000 | -3.18062800 | 2.31987000 |
| C | 0.95815500 | -1.24894300 | 2.97467000 |
| C | 0.95840800 | -0.27020200 | 3.98322800 |
| H | 1.39816800 | 0.55498800 | 3.81764400 |
| C | 0.34045200 | -0.46257300 | 5.21747000 |
| H | 0.37629100 | 0.22258400 | 5.87478600 |
| C | -0.32330400 | -1.63978800 | 5.49069800 |
| H | -0.72627600 | -1.78239600 | 6.33916000 |
| C | -0.39137600 | -2.60980800 | 4.50651900 |
| H | -0.86717500 | -3.41540700 | 4.67137900 |
| C | 0.23150300 | -2.41574600 | 3.27550900 |
| H | 0.16324300 | -3.09565400 | 2.61565400 |
| C | -0.27850000 | 3.86087200 | 2.68783500 |
| H | -0.06483600 | 4.75730200 | 3.02229100 |
| H | 0.34812100 | 3.21186800 | 3.06975900 |
| H | -1.19306700 | 3.62485300 | 2.94689400 |
| C | -1.28791700 | 5.16338300 | 0.38432200 |
| H | -1.03082800 | 6.00092500 | 0.82367700 |
| H | -2.19878200 | 4.92056500 | 0.65156200 |
| H | -1.25361900 | 5.28149900 | -0.58835300 |
| C | 1.45668300 | 4.67345700 | 0.62483300 |
| H | 1.44655300 | 5.54336300 | 1.07608300 |
| H | 1.60976700 | 4.80439000 | -0.33407400 |
| H | 2.17484000 | 4.12050200 | 0.99783300 |
| B | -1.58026800 | -1.51530700 | -1.29908600 |
| B | 1.79609800 | -1.03576200 | 1.62613700 |
| Cu | -0.18367600 | 1.71751500 | 0.11209400 |
| Cu | 0.08813600 | -0.99172600 | 0.14067400 |
| N | -3.54179800 | 0.43236300 | 0.02466400 |
| N | -4.47149800 | -1.65530500 | -0.66851100 |
| N | 3.29526300 | 0.74556800 | -0.45405600 |
| N | 4.40373300 | -1.27873500 | 0.21337700 |
| P | -3.13762000 | -2.58532700 | -1.01600500 |
| P | 3.43558100 | -1.97356700 | 1.36137700 |
| P | -0.13879800 | 3.84295000 | 0.87544800 |
| Si | -1.83565200 | 0.26468000 | -0.62208900 |
| Si | 1.80872000 | 0.60747200 | 0.61833800 |

---------------------------------------------------------------------

```
Excitation energies and oscillator strengths:
 ----------------------------------------------------------------------------------
 Excited state symmetry could not be determined.
 Excited State   1:      Triplet-?Sym    1.6816 eV  737.30 nm  f=0.0000  <S**2>=2.000
     338 -> 342       -0.10870
     338 -> 343        0.13056
     340 -> 342        0.38968
     340 -> 343       -0.28373
     340 -> 344        0.13606
     341 -> 342        0.20487
     341 -> 343       -0.33274
     341 -> 344        0.11281
 This state for optimization and/or second-order correction.
 Total Energy, E(TD-HF/TD-DFT) =  -7840.49279251
 Copying the excited state density for this state as the 1-particle RhoCI density.
----------------------------------------------------------------------------------
 Excited state symmetry could not be determined.
 Excited State   2:      Triplet-?Sym    1.7845 eV  694.79 nm  f=0.0000  <S**2>=2.000
     339 -> 342       -0.16126
     340 -> 342       -0.18436
     340 -> 343       -0.14575
     341 -> 342        0.54959
     341 -> 343        0.24668
     341 -> 345       -0.11246
----------------------------------------------------------------------------------
 Excited state symmetry could not be determined.
 Excited State   3:      Triplet-?Sym    2.0738 eV  597.85 nm  f=0.0000  <S**2>=2.000
     339 -> 342       -0.17219
     339 -> 343        0.28204
     339 -> 344       -0.10702
     340 -> 342        0.23584
     340 -> 343       -0.19475
     340 -> 344        0.11266
     341 -> 342       -0.18483
     341 -> 343        0.39061
     341 -> 344       -0.14965
----------------------------------------------------------------------------------
 Excited state symmetry could not be determined.
 Excited State   4:      Triplet-?Sym    2.3087 eV  537.02 nm  f=0.0000  <S**2>=2.000
     338 -> 342        0.19625
     338 -> 343        0.10663
     339 -> 342        0.33588
     340 -> 342        0.32468
     340 -> 343        0.30711
     340 -> 345       -0.10005
     341 -> 342        0.15891
     341 -> 343        0.19779
----------------------------------------------------------------------------------
 Excited state symmetry could not be determined.
 Excited State   5:      Singlet-?Sym    2.4642 eV  503.15 nm  f=0.0141  <S**2>=0.000
     339 -> 342        0.15788
     339 -> 343       -0.12439
     341 -> 342        0.61397
     341 -> 343       -0.18832
     341 -> 344        0.10884
----------------------------------------------------------------------------------
 Excited state symmetry could not be determined.
 Excited State   6:      Singlet-?Sym    2.6337 eV  470.76 nm  f=0.0208  <S**2>=0.000
     338 -> 342        0.14285
     339 -> 343        0.18011
     340 -> 342        0.11201
```

```
     341 -> 342        0.23632
     341 -> 343        0.57950
     341 -> 344       -0.10680
 ---------------------------------------------------------------------------------
 Excited state symmetry could not be determined.
 Excited State   7:      Triplet-?Sym    2.6619 eV  465.77 nm  f=0.0000  <S**2>=2.000
     338 -> 342        0.14393
     339 -> 342        0.15978
     339 -> 343        0.48896
     339 -> 344       -0.13025
     339 -> 345       -0.13819
     340 -> 350        0.14801
     341 -> 342        0.10797
     341 -> 343       -0.10758
 ---------------------------------------------------------------------------------
 Excited state symmetry could not be determined.
 Excited State   8:      Triplet-?Sym    2.7409 eV  452.34 nm  f=0.0000  <S**2>=2.000
     339 -> 342        0.44199
     339 -> 344        0.10588
     339 -> 345       -0.10204
     340 -> 342       -0.13236
     340 -> 343       -0.33563
     340 -> 344        0.10441
     340 -> 345        0.12214
 ---------------------------------------------------------------------------------
 Excited state symmetry could not be determined.
 Excited State   9:      Singlet-?Sym    2.7923 eV  444.02 nm  f=0.0336  <S**2>=0.000
     338 -> 342        0.10272
     339 -> 342        0.13902
     340 -> 342        0.20844
     340 -> 343        0.58060
     340 -> 344       -0.11605
     340 -> 345       -0.10099
     341 -> 342       -0.12495
 ---------------------------------------------------------------------------------
 Excited state symmetry could not be determined.
 Excited State  10:      Singlet-?Sym    2.8959 eV  428.14 nm  f=0.0621  <S**2>=0.000
     338 -> 342        0.21674
     339 -> 343        0.34058
     340 -> 342        0.45662
     340 -> 343       -0.12523
     341 -> 343       -0.24393
 ---------------------------------------------------------------------------------
 Excited state symmetry could not be determined.
 Excited State  11:      Triplet-?Sym    3.1225 eV  397.06 nm  f=0.0000  <S**2>=2.000
     338 -> 342        0.45062
     338 -> 343        0.13821
     338 -> 345       -0.10175
     339 -> 343       -0.12535
     340 -> 343       -0.22187
     341 -> 342       -0.10859
     341 -> 345        0.16194
     341 -> 350        0.11851
     341 -> 355        0.10541
 ---------------------------------------------------------------------------------
 Excited state symmetry could not be determined.
 Excited State  12:      Singlet-?Sym    3.2003 eV  387.41 nm  f=0.0144  <S**2>=0.000
     339 -> 342        0.38975
     339 -> 343        0.43988
     339 -> 345       -0.13496
     340 -> 342       -0.24922
```

```
 ---------------------------------------------------------------------------------
 Excited state symmetry could not be determined.
 Excited State  13:      Singlet-?Sym    3.2621 eV  380.08 nm  f=0.0932  <S**2>=0.000
     339 -> 342        0.45074
     339 -> 343       -0.23383
     340 -> 342        0.24019
     340 -> 343       -0.25303
     341 -> 342       -0.14218
     341 -> 343        0.10833
     341 -> 344       -0.11021
     341 -> 345       -0.10438
 ---------------------------------------------------------------------------------
 Excited state symmetry could not be determined.
 Excited State  14:      Triplet-?Sym    3.2879 eV  377.09 nm  f=0.0000  <S**2>=2.000
     338 -> 342       -0.15621
     338 -> 343        0.45356
     338 -> 344       -0.10092
     340 -> 344       -0.14949
     341 -> 344       -0.32886
 ---------------------------------------------------------------------------------
 Excited state symmetry could not be determined.
 Excited State  15:      Triplet-?Sym    3.3530 eV  369.77 nm  f=0.0000  <S**2>=2.000
     338 -> 342       -0.12126
     338 -> 344       -0.15251
     339 -> 345       -0.12348
     340 -> 342       -0.10693
     340 -> 344        0.12019
     340 -> 345       -0.10283
     341 -> 343        0.15051
     341 -> 344        0.21848
     341 -> 345        0.35553
     341 -> 348       -0.12064
     341 -> 355        0.11882
     341 -> 359        0.11335
 ---------------------------------------------------------------------------------
 Excited state symmetry could not be determined.
 Excited State  16:      Triplet-?Sym    3.3813 eV  366.68 nm  f=0.0000  <S**2>=2.000
     338 -> 343        0.18983
     338 -> 344       -0.17607
     338 -> 345       -0.10713
     340 -> 345        0.12032
     341 -> 342       -0.15139
     341 -> 343        0.13308
     341 -> 344        0.35673
     341 -> 345       -0.27846
     341 -> 350        0.15157
     341 -> 357        0.14986
 ---------------------------------------------------------------------------------
 Excited state symmetry could not be determined.
 Excited State  17:      Singlet-?Sym    3.5265 eV  351.57 nm  f=0.0155  <S**2>=0.000
     338 -> 342        0.47334
     339 -> 342        0.12768
     340 -> 342       -0.21819
     340 -> 344        0.10649
     341 -> 344        0.11595
     341 -> 345        0.29548
     341 -> 355        0.11411
 ---------------------------------------------------------------------------------
 Excited state symmetry could not be determined.
 Excited State  18:      Singlet-?Sym    3.5714 eV  347.16 nm  f=0.1587  <S**2>=0.000
     338 -> 343       -0.34280
```

```
    340 -> 344         0.16890
    341 -> 344         0.51269
    341 -> 357         0.10433
---------------------------------------------------------------------------------
 Excited state symmetry could not be determined.
 Excited State  19:      Singlet-?Sym    3.7678 eV  329.06 nm  f=0.0897  <S**2>=0.000
    338 -> 342        -0.15314
    338 -> 343        -0.32321
    338 -> 345         0.15067
    339 -> 342         0.15410
    340 -> 342         0.11043
    340 -> 345        -0.11473
    341 -> 344        -0.15978
    341 -> 345         0.43614
    341 -> 350        -0.11228
---------------------------------------------------------------------------------
 Excited state symmetry could not be determined.
 Excited State  20:      Singlet-?Sym    3.8043 eV  325.90 nm  f=0.0245  <S**2>=0.000
    338 -> 342        -0.27301
    338 -> 343         0.33401
    338 -> 344        -0.19364
    339 -> 344         0.12976
    340 -> 342         0.10558
    341 -> 343         0.13853
    341 -> 344         0.27483
    341 -> 345         0.28422
---------------------------------------------------------------------------------
```

**Table S5.** Cartesian coordinates and theoretical UV-Vis spectrum for **5**.

**M06-2X/def2-TZVP**

| Atomic Number | X | Y | Z |
|---|---|---|---|
| C | -0.22754904 | -0.75063164 | 2.81814832 |
| H | 0.07079416 | -1.68335597 | 2.95616730 |
| H | 0.32670949 | -0.16369575 | 3.39174765 |
| C | -1.71123970 | -0.61976409 | 3.23780491 |
| H | -1.97278804 | 0.33395277 | 3.09368865 |
| C | -2.57121260 | -1.46317465 | 2.30993539 |
| C | -3.35250346 | -2.50702906 | 2.76461967 |
| H | -3.41971192 | -2.67275230 | 3.69667847 |
| C | -4.04595318 | -3.32221329 | 1.86296259 |
| H | -4.60283610 | -4.02086445 | 2.18601628 |
| C | -3.92220659 | -3.11149019 | 0.50420863 |
| H | -4.37135179 | -3.69327639 | -0.09788373 |
| C | -3.15150751 | -2.06243005 | -0.00733468 |
| C | -2.50229352 | -1.21198563 | 0.92147571 |
| C | -2.99248012 | -1.90636369 | -1.50076711 |
| H | -2.57204684 | -1.01722763 | -1.67961690 |
| C | -2.05647746 | -3.00730706 | -2.06121414 |
| H | -1.93873068 | -2.87670739 | -3.02573288 |
| H | -2.45257027 | -3.88853891 | -1.89737931 |
| H | -1.18498415 | -2.95302236 | -1.61610812 |
| C | -4.35220398 | -1.95274740 | -2.23350646 |
| H | -4.22158054 | -1.73043368 | -3.17870334 |
| H | -4.96452990 | -1.30494251 | -1.82668390 |
| H | -4.73255746 | -2.85309137 | -2.15908489 |
| C | -2.19670070 | 0.95454832 | -0.18225383 |
| C | -3.70130769 | 1.14320462 | -0.21698221 |
| C | -4.30848613 | 1.46461276 | -1.41299874 |
| H | -3.79045579 | 1.50741697 | -2.20907082 |
| C | -5.67964867 | 1.72570805 | -1.46239631 |
| H | -6.09527189 | 1.91873891 | -2.29463500 |
| C | -6.42485274 | 1.70515324 | -0.31762527 |
| H | -7.34951103 | 1.92349561 | -0.34390478 |
| C | -5.82179949 | 1.36243200 | 0.88156625 |
| H | -6.34475383 | 1.33049649 | 1.67329495 |
| C | -4.47513071 | 1.06812111 | 0.94897629 |
| H | -4.07944572 | 0.81798102 | 1.77559616 |
| C | 0.54657038 | -0.46551569 | -2.10770663 |
| H | 0.86204878 | -0.97767295 | -2.89378039 |
| H | -0.38893513 | -0.74442328 | -1.94177018 |
| C | 0.50728020 | 1.01823247 | -2.52206331 |
| C | -0.58726302 | 1.18110454 | -3.59873528 |
| H | -0.41693257 | 0.55862425 | -4.33565612 |
| H | -0.57687947 | 2.10062161 | -3.93852514 |
| H | -1.46420131 | 0.98915202 | -3.20479686 |
| C | 1.86206656 | 1.43474016 | -3.11295458 |
| H | 2.15076297 | 0.76702658 | -3.77117365 |
| H | 2.52765372 | 1.49375539 | -2.39611262 |
| H | 1.77429939 | 2.30671206 | -3.55050007 |
| C | 0.34574519 | 3.84290180 | -1.18649814 |
| C | -0.36653631 | 4.49710005 | 0.00502407 |
| H | -0.30399853 | 5.47257188 | -0.07171535 |
| H | 0.05996873 | 4.20852922 | 0.83975138 |
| H | -1.30861792 | 4.22956850 | 0.01054809 |
| C | 1.82797525 | 4.23527289 | -1.16894513 |
| H | 1.91036208 | 5.20775484 | -1.25454889 |
| H | 2.28998987 | 3.80155033 | -1.91750850 |
| H | 2.23322538 | 3.94589187 | -0.32475488 |
| C | -0.32410213 | 4.33998479 | -2.47254496 |
| H | -0.34428744 | 5.31973744 | -2.47319582 |
| H | -1.24138541 | 3.99519485 | -2.51671118 |
| H | 0.18123845 | 4.02250397 | -3.24910035 |
| C | 1.94746065 | 2.08165163 | 1.51908762 |

| | | | |
|---|---|---|---|
| C | 1.30233617 | 2.64732298 | 2.62575288 |
| H | 0.37823680 | 2.47204975 | 2.75453370 |
| C | 1.96395372 | 3.45471594 | 3.54288618 |
| H | 1.48528771 | 3.83846508 | 4.26727917 |
| C | 3.32426408 | 3.69951202 | 3.40058574 |
| H | 3.78020385 | 4.25473184 | 4.02248574 |
| C | 4.00404318 | 3.12298291 | 2.33812434 |
| H | 4.93850942 | 3.27067940 | 2.24045629 |
| C | 3.32893377 | 2.33095621 | 1.41540271 |
| H | 3.81500738 | 1.94758558 | 0.69425812 |
| C | 3.32677456 | -1.59435965 | -1.73170559 |
| H | 3.31266390 | -1.09844214 | -2.59999742 |
| C | 2.38622707 | -2.66627323 | -1.66144109 |
| H | 1.84800058 | -2.77926205 | -2.49740216 |
| C | 2.66722840 | -3.97666259 | -0.94201310 |
| H | 1.84526473 | -4.52804437 | -0.94026817 |
| H | 3.36511779 | -4.47435144 | -1.43749336 |
| C | -1.90350514 | -0.91657032 | 4.71763355 |
| H | -2.84296254 | -0.77930294 | 4.95787413 |
| H | -1.33868363 | -0.31585808 | 5.24673573 |
| H | -1.65170758 | -1.84631461 | 4.89919257 |
| C | 3.12675709 | -3.77064516 | 0.48502707 |
| H | 4.11672143 | -3.78058385 | 0.50669353 |
| H | 2.80600334 | -4.52703602 | 1.03672531 |
| C | 2.63730145 | -2.47614777 | 1.09860746 |
| H | 2.09610058 | -2.59419069 | 1.93030121 |
| C | 3.36410910 | -1.28579457 | 1.05040688 |
| H | 3.25691367 | -0.70614639 | 1.85846078 |
| C | 4.69295947 | -1.14135038 | 0.35895080 |
| H | 5.37890369 | -1.63101081 | 0.87923758 |
| H | 4.94776565 | -0.18471309 | 0.35261594 |
| C | 4.70322876 | -1.66282650 | -1.07786210 |
| H | 5.34170502 | -1.12680207 | -1.61174552 |
| H | 5.01675954 | -2.60064950 | -1.07986237 |
| B | 1.18756904 | 1.21592129 | 0.40969281 |
| Ir | 1.70046460 | -1.11273560 | -0.43250744 |
| N | -1.68085804 | -0.12005132 | 0.49732220 |
| N | -1.53327117 | 1.88505371 | -0.80240432 |
| P | 0.13323428 | 2.00090436 | -0.98872266 |
| Si | 0.02433855 | -0.27739130 | 1.02201979 |

```
Excitation energies and oscillator strengths:
Excited state symmetry could not be determined.
 Excited State   1:      Triplet-?Sym    0.5965 eV 2078.48 nm  f=0.0000  <S**2>=2.000
     183 -> 184        0.72208
     183 <- 184        0.18298
 This state for optimization and/or second-order correction.
 Total Energy, E(TD-HF/TD-DFT) =  -2464.25900166
 Copying the excited state density for this state as the 1-particle RhoCI density.
---------------------------------------------------------------------
 Excited state symmetry could not be determined.
 Excited State   2:      Triplet-?Sym    0.7983 eV 1553.02 nm  f=0.0000  <S**2>=2.000
     177 -> 184       -0.24998
     178 -> 184       -0.11118
     179 -> 184        0.10838
     182 -> 184        0.67837
     177 <- 184       -0.10434
     182 <- 184        0.23601
---------------------------------------------------------------------
 Excited state symmetry could not be determined.
 Excited State   3:      Singlet-?Sym    1.1643 eV 1064.84 nm  f=0.0017  <S**2>=0.000
     177 -> 184        0.10133
     182 -> 184       -0.27622
     183 -> 184        0.63634
---------------------------------------------------------------------
 Excited state symmetry could not be determined.
 Excited State   4:      Triplet-?Sym    2.1190 eV  585.10 nm  f=0.0000  <S**2>=2.000
     178 -> 184        0.40020
     179 -> 184        0.53824
     181 -> 184        0.14502
---------------------------------------------------------------------
 Excited state symmetry could not be determined.
 Excited State   5:      Singlet-?Sym    2.1455 eV  577.89 nm  f=0.0245  <S**2>=0.000
     179 -> 184        0.25077
     182 -> 184        0.56978
     183 -> 184        0.29663
---------------------------------------------------------------------
 Excited state symmetry could not be determined.
 Excited State   6:      Triplet-?Sym    2.3087 eV  537.03 nm  f=0.0000  <S**2>=2.000
     177 -> 184       -0.28157
     178 -> 184        0.46859
     179 -> 184       -0.32725
     180 -> 184        0.24313
---------------------------------------------------------------------
 Excited state symmetry could not be determined.
 Excited State   7:      Singlet-?Sym    2.4073 eV  515.04 nm  f=0.0059  <S**2>=0.000
     177 -> 184        0.24674
     179 -> 184        0.54563
     180 -> 184       -0.19552
     181 -> 184        0.13467
     182 -> 184       -0.20794
---------------------------------------------------------------------
 Excited state symmetry could not be determined.
 Excited State   8:      Singlet-?Sym    2.6510 eV  467.69 nm  f=0.0233  <S**2>=0.000
     177 -> 184       -0.15833
     178 -> 184        0.62077
     179 -> 184        0.21086
     180 -> 184        0.12872
---------------------------------------------------------------------
 Excited state symmetry could not be determined.
 Excited State   9:      Triplet-?Sym    2.9137 eV  425.52 nm  f=0.0000  <S**2>=2.000
     177 -> 184       -0.29674
     178 -> 184       -0.20374
     180 -> 184        0.16888
     181 -> 184        0.52029
     182 -> 184       -0.13246
---------------------------------------------------------------------
 Excited state symmetry could not be determined.
 Excited State  10:      Triplet-?Sym    2.9629 eV  418.46 nm  f=0.0000  <S**2>=2.000
     170 -> 184       -0.20288
     171 -> 184       -0.28639
     172 -> 184        0.35494
```

```
     173 -> 184       -0.25004
     177 -> 184       -0.24749
     180 -> 184       -0.24729
 ---------------------------------------------------------------------
 Excited state symmetry could not be determined.
 Excited State  11:      Singlet-?Sym    3.1520 eV  393.35 nm  f=0.0106  <S**2>=0.000
     177 -> 184       -0.41940
     178 -> 184       -0.22101
     180 -> 184        0.12788
     181 -> 184        0.46920
 ---------------------------------------------------------------------
 Excited state symmetry could not be determined.
 Excited State  12:      Triplet-?Sym    3.1609 eV  392.25 nm  f=0.0000  <S**2>=2.000
     170 -> 184        0.15923
     172 -> 184        0.21850
     173 -> 184       -0.10585
     177 -> 184        0.32381
     178 -> 184        0.15715
     179 -> 184       -0.17872
     180 -> 184       -0.11991
     181 -> 184        0.34542
     182 -> 184        0.23438
     183 -> 189        0.12294
 ---------------------------------------------------------------------
 Excited state symmetry could not be determined.
 Excited State  13:      Triplet-?Sym    3.2017 eV  387.25 nm  f=0.0000  <S**2>=2.000
     183 -> 185        0.62467
     183 -> 191        0.17974
     183 -> 192       -0.15066
 ---------------------------------------------------------------------
 Excited state symmetry could not be determined.
 Excited State  14:      Singlet-?Sym    3.3814 eV  366.66 nm  f=0.0132  <S**2>=0.000
     183 -> 185        0.65480
     183 -> 191        0.15596
     183 -> 192       -0.12938
 ---------------------------------------------------------------------
 Excited state symmetry could not be determined.
 Excited State  15:      Singlet-?Sym    3.5406 eV  350.17 nm  f=0.0157  <S**2>=0.000
     172 -> 184        0.15134
     177 -> 184        0.29713
     178 -> 184        0.15015
     179 -> 184       -0.20129
     180 -> 184       -0.17717
     181 -> 184        0.45984
     182 -> 184        0.18019
 ---------------------------------------------------------------------
 Excited state symmetry could not be determined.
 Excited State  16:      Singlet-?Sym    3.6413 eV  340.50 nm  f=0.1291  <S**2>=0.000
     170 -> 184        0.15803
     171 -> 184        0.20636
     172 -> 184       -0.24110
     173 -> 184        0.20483
     174 -> 184       -0.10820
     177 -> 184        0.22403
     180 -> 184        0.47403
 ---------------------------------------------------------------------
 Excited state symmetry could not be determined.
 Excited State  17:      Triplet-?Sym    3.8132 eV  325.15 nm  f=0.0000  <S**2>=2.000
     169 -> 184       -0.13103
     170 -> 184       -0.12961
     171 -> 184       -0.13983
     172 -> 184        0.10362
     180 -> 184        0.35854
     181 -> 189       -0.12860
     183 -> 187        0.14582
     183 -> 188        0.12897
     183 -> 189        0.35255
     183 -> 190        0.13051
 ---------------------------------------------------------------------
 Excited state symmetry could not be determined.
 Excited State  18:      Triplet-?Sym    3.8308 eV  323.65 nm  f=0.0000  <S**2>=2.000
```

```
     172 -> 184         0.25483
     174 -> 184        -0.10598
     177 -> 184         0.10533
     180 -> 184         0.37128
     183 -> 187        -0.15649
     183 -> 188        -0.16324
     183 -> 189        -0.23886
     183 -> 190        -0.21326
---------------------------------------------------------------------
 Excited state symmetry could not be determined.
 Excited State  19:      Singlet-?Sym    4.0667 eV  304.88 nm  f=0.0362  <S**2>=0.000
     183 -> 187         0.22695
     183 -> 188         0.18958
     183 -> 189         0.58522
     183 -> 190         0.14606
---------------------------------------------------------------------
 Excited state symmetry could not be determined.
 Excited State  20:      Singlet-?Sym    4.1245 eV  300.60 nm  f=0.0292  <S**2>=0.000
     169 -> 184        -0.12762
     171 -> 184        -0.22254
     172 -> 184         0.42914
     173 -> 184        -0.19147
     174 -> 184        -0.14006
     177 -> 184         0.10660
     180 -> 184         0.35895
---------------------------------------------------------------------
```

**Table S6.** Cartesian coordinates and theoretical UV-Vis spectrum for **6**.

**M06-2X/def2-TZVP**

| Atomic Number | X | Y | Z |
|---|---|---|---|
| C | 0.00000000 | 0.00000000 | 0.00000000 |
| C | 0.00000000 | 0.00000000 | 1.38989100 |
| C | 1.20511300 | 0.00000000 | 2.08459500 |
| H | 1.20486000 | -0.00031200 | 3.03462400 |
| C | 2.40783500 | -0.00022100 | 1.38932400 |
| H | 3.23091700 | 0.00003800 | 1.86390200 |
| C | 2.40695900 | 0.00007700 | -0.00077300 |
| H | 3.23007700 | 0.00038600 | -0.47606400 |
| C | 1.20336200 | 0.00059800 | -0.69559900 |
| C | 1.27597600 | -0.05539200 | -2.22709600 |
| H | 0.34029400 | -0.11194800 | -2.57435500 |
| C | 1.91052400 | 1.21946500 | -2.79962400 |
| H | 1.94289200 | 1.15762600 | -3.77757200 |
| H | 2.82070300 | 1.31781700 | -2.44910800 |
| H | 1.37456700 | 1.99721600 | -2.53939000 |
| C | 2.03601300 | -1.30085600 | -2.72037900 |
| H | 1.99808500 | -1.33929800 | -3.69935300 |
| H | 1.62210600 | -2.10562300 | -2.34568300 |
| H | 2.97087500 | -1.24863100 | -2.43187000 |
| C | -1.85361200 | -1.01382800 | -1.23239800 |
| C | -0.97560400 | -2.26263400 | -1.38671700 |
| C | -0.34683500 | -2.89895500 | -0.32454300 |
| C | 0.32749300 | -4.09690800 | -0.52946300 |
| H | 0.75680200 | -4.53142400 | 0.19692100 |
| C | 0.37307300 | -4.65777000 | -1.79980100 |
| H | 0.83500700 | -5.47645500 | -1.94231800 |
| C | -0.25492600 | -4.02200200 | -2.86487200 |
| H | -0.22268800 | -4.40545700 | -3.73389400 |
| C | -0.92925400 | -2.82404900 | -2.65995200 |
| H | -1.35859500 | -2.38940700 | -3.38626800 |
| C | -4.73528700 | -0.40428500 | -3.83243600 |
| C | -3.58078100 | -0.02578400 | -4.76292100 |
| H | -3.79171800 | -0.30863500 | -5.67734400 |
| H | -2.76070900 | -0.47114900 | -4.46422000 |
| H | -3.45068400 | 0.94552300 | -4.74358900 |
| C | -5.01483200 | -1.88873800 | -4.01096200 |
| H | -5.07077000 | -2.09750600 | -4.96812400 |
| H | -5.86358300 | -2.11673400 | -3.57813000 |
| H | -4.29047500 | -2.40927700 | -3.60614000 |
| C | -5.98398600 | 0.41439200 | -4.20180000 |
| H | -6.15533400 | 0.33254100 | -5.16312700 |
| H | -5.83499800 | 1.35633100 | -3.97556800 |
| H | -6.75591900 | 0.07642600 | -3.70201700 |
| C | -5.48687100 | -0.23660500 | -0.83929500 |
| C | -6.27055900 | -1.52221800 | -1.04773000 |
| H | -6.75399800 | -1.74815500 | -0.22615500 |
| H | -5.65275300 | -2.24891500 | -1.27380600 |
| H | -6.91118500 | -1.39978500 | -1.78017000 |
| C | -6.42350800 | 0.96777300 | -0.77344500 |
| H | -7.12316200 | 0.80102700 | -0.10801200 |
| H | -6.83421100 | 1.11049100 | -1.65130100 |
| H | -5.91299500 | 1.76462400 | -0.51793000 |
| C | -4.75114000 | -0.33347800 | 0.52172700 |
| H | -5.40827100 | -0.35758400 | 1.24797100 |
| H | -4.16785900 | 0.44621900 | 0.63451000 |
| H | -4.21041100 | -1.15093500 | 0.54312700 |
| C | -4.11130200 | 3.13659500 | -2.68356700 |
| C | -4.69991900 | 4.07426400 | -1.84198000 |
| C | -5.15462400 | 5.28266300 | -2.35659000 |
| C | -5.02071200 | 5.55339400 | -3.71278700 |
| C | -4.43269100 | 4.61693700 | -4.55530100 |
| C | -3.97814000 | 3.40864800 | -4.04011200 |

| | | | |
|---|---|---|---|
| C | -0.42046400 | 4.68277700 | -1.91977300 |
| H | -0.05717000 | 5.55685600 | -1.66644300 |
| H | -1.26623100 | 4.80722200 | -2.39944700 |
| H | 0.21921700 | 4.22084500 | -2.50294600 |
| C | -1.88475700 | 4.64619600 | 0.70785200 |
| H | -1.46049700 | 5.49368800 | 0.95672000 |
| H | -2.08723400 | 4.13121600 | 1.51522100 |
| H | -2.71418500 | 4.82768700 | 0.21794300 |
| C | 0.89357600 | 3.38883500 | 0.51811300 |
| H | 1.24575600 | 4.25065300 | 0.82506600 |
| H | 1.54076700 | 2.96756500 | -0.08473600 |
| H | 0.73961700 | 2.80578400 | 1.29113600 |
| B | -3.47807800 | 1.82218800 | -2.04222800 |
| N | -1.26220500 | 0.08327900 | -0.69773900 |
| N | -3.00633800 | -1.06513300 | -1.82131900 |
| P | -4.15969800 | 0.07167400 | -2.12344000 |
| Si | -1.86133000 | 1.74363300 | -1.11523000 |
| Si | -0.71645100 | 3.66088400 | -0.38677400 |
| C | -1.35143400 | 0.13368700 | 2.09679700 |
| H | -2.14871300 | 0.21255600 | 1.34890600 |
| C | -1.59335000 | -1.09854000 | 2.97261400 |
| H | -0.79607100 | -1.17740900 | 3.72050500 |
| H | -1.59635700 | -1.99749000 | 2.34564100 |
| H | -2.56080300 | -1.00283600 | 3.47866800 |
| C | -1.34723300 | 1.38942900 | 2.97261300 |
| H | -0.54995400 | 1.31056100 | 3.72050500 |
| H | -2.31468700 | 1.48513200 | 3.47866700 |
| H | -1.17405300 | 2.27154600 | 2.34564000 |
| H | -0.38403100 | -2.45669300 | 0.67757300 |
| H | -4.80478100 | 3.86037000 | -0.77218200 |
| H | -3.51394800 | 2.66963500 | -4.70313700 |
| H | -4.32686300 | 4.83036200 | -5.62509800 |
| H | -5.61818900 | 6.02177700 | -1.69323900 |
| H | -5.37874700 | 6.50584300 | -4.12005600 |

---------------------------------------------------------------------

```
Excitation energies and oscillator strengths:
----------------------------------------------------------------------------------------------------
---------
 Excited state symmetry could not be determined.
 Excited State   1:      Triplet-?Sym    1.6702 eV  742.32 nm  f=0.0000  <S**2>=2.000
     166 -> 167        0.52897
     166 -> 168        0.40219
     166 -> 170       -0.19554
     166 -> 171       -0.13690
     166 <- 167        0.11138
     166 <- 168        0.10025
 This state for optimization and/or second-order correction.
 Total Energy, E(TD-HF/TD-DFT) =  -2458.47637398
 Copying the excited state density for this state as the 1-particle RhoCI density.
----------------------------------------------------------------------------------------------------
---------
 Excited state symmetry could not be determined.
 Excited State   2:      Triplet-?Sym    3.1021 eV  399.68 nm  f=0.0000  <S**2>=2.000
     166 -> 167       -0.38804
     166 -> 168        0.43179
     166 -> 170       -0.19227
     166 -> 172        0.13532
     166 -> 174       -0.23907
     166 -> 176        0.10382
----------------------------------------------------------------------------------------------------
---------
 Excited state symmetry could not be determined.
 Excited State   3:      Singlet-?Sym    3.1653 eV  391.70 nm  f=0.1374  <S**2>=0.000
     166 -> 167        0.67108
     166 -> 168        0.10818
----------------------------------------------------------------------------------------------------
---------
 Excited state symmetry could not be determined.
 Excited State   4:      Triplet-?Sym    3.2706 eV  379.09 nm  f=0.0000  <S**2>=2.000
     166 -> 168       -0.24298
     166 -> 170       -0.29291
     166 -> 171        0.15387
     166 -> 172        0.43648
     166 -> 173        0.10933
     166 -> 174       -0.10082
     166 -> 176       -0.21211
     166 -> 177        0.14393
     166 -> 179        0.11451
----------------------------------------------------------------------------------------------------
---------
 Excited state symmetry could not be determined.
 Excited State   5:      Singlet-?Sym    3.6970 eV  335.36 nm  f=0.1371  <S**2>=0.000
     166 -> 168        0.58911
     166 -> 170       -0.16101
     166 -> 172        0.16769
     166 -> 174       -0.20947
     166 -> 175        0.15066
----------------------------------------------------------------------------------------------------
---------
 Excited state symmetry could not be determined.
 Excited State   6:      Singlet-?Sym    3.7340 eV  332.04 nm  f=0.0123  <S**2>=0.000
     166 -> 168       -0.29712
     166 -> 170       -0.30612
     166 -> 171        0.13927
     166 -> 172        0.44825
     166 -> 175        0.22510
     166 -> 176       -0.10517
----------------------------------------------------------------------------------------------------
---------
 Excited state symmetry could not be determined.
 Excited State   7:      Triplet-?Sym    3.8046 eV  325.88 nm  f=0.0000  <S**2>=2.000
     166 -> 169        0.10170
     166 -> 170        0.12230
     166 -> 171       -0.14964
     166 -> 172        0.13580
     166 -> 173       -0.22332
```

```
    166 -> 174       -0.15160
    166 -> 175        0.55080
    166 -> 180       -0.10912
-------------------------------------------------------------------------------------------------
---------
 Excited state symmetry could not be determined.
 Excited State   8:      Triplet-?Sym    3.9664 eV  312.58 nm  f=0.0000  <S**2>=2.000
    159 -> 167        0.13763
    165 -> 167        0.47390
    165 -> 168        0.35166
    165 -> 171       -0.15509
-------------------------------------------------------------------------------------------------
---------
 Excited state symmetry could not be determined.
 Excited State   9:      Triplet-?Sym    4.1055 eV  301.99 nm  f=0.0000  <S**2>=2.000
    163 -> 167        0.10611
    163 -> 171        0.15122
    165 -> 167        0.11234
    166 -> 169        0.40992
    166 -> 170       -0.22836
    166 -> 172       -0.15499
    166 -> 173        0.17543
    166 -> 175        0.14685
    166 -> 176        0.16272
-------------------------------------------------------------------------------------------------
---------
 Excited state symmetry could not be determined.
 Excited State  10:      Singlet-?Sym    4.1260 eV  300.49 nm  f=0.0534  <S**2>=0.000
    166 -> 170        0.36787
    166 -> 171       -0.20785
    166 -> 172        0.11540
    166 -> 173       -0.36296
    166 -> 174       -0.16738
    166 -> 175        0.28742
    166 -> 176       -0.16052
-------------------------------------------------------------------------------------------------
---------
 Excited state symmetry could not be determined.
 Excited State  11:      Triplet-?Sym    4.1307 eV  300.15 nm  f=0.0000  <S**2>=2.000
    158 -> 167       -0.15782
    158 -> 168        0.15765
    158 -> 169        0.12344
    158 -> 174        0.11542
    160 -> 167        0.15262
    160 -> 168       -0.10401
    160 -> 169        0.16351
    160 -> 171       -0.12891
    161 -> 167        0.18550
    164 -> 167        0.27519
    164 -> 168       -0.16623
    165 -> 168       -0.11221
    166 -> 172        0.10503
    166 -> 174        0.18828
-------------------------------------------------------------------------------------------------
---------
 Excited state symmetry could not be determined.
 Excited State  12:      Triplet-?Sym    4.1548 eV  298.41 nm  f=0.0000  <S**2>=2.000
    157 -> 170        0.10628
    163 -> 167        0.25174
    163 -> 169        0.14566
    163 -> 170        0.10627
    163 -> 171        0.32495
    164 -> 168        0.11160
    164 -> 170        0.23278
    166 -> 170        0.15294
    166 -> 173       -0.16130
    166 -> 175       -0.11098
-------------------------------------------------------------------------------------------------
---------
 Excited state symmetry could not be determined.
 Excited State  13:      Triplet-?Sym    4.2057 eV  294.80 nm  f=0.0000  <S**2>=2.000
```

```
     159 -> 168         0.10276
     161 -> 167        -0.10812
     162 -> 172         0.13802
     162 -> 173        -0.17599
     164 -> 167        -0.11156
     165 -> 167         0.13943
     165 -> 170        -0.15871
     165 -> 172         0.21445
     165 -> 173         0.11033
     165 -> 174         0.11666
     166 -> 170         0.23561
     166 -> 171         0.19610
     166 -> 172         0.22267
     166 -> 176         0.17129
---------------------------------------------------------------------------------------------------
---------
 Excited state symmetry could not be determined.
 Excited State  14:      Singlet-?Sym    4.2252 eV  293.44 nm  f=0.0009  <S**2>=0.000
     159 -> 167         0.10506
     165 -> 167         0.47156
     165 -> 168         0.29053
     165 -> 170        -0.13265
     165 -> 171        -0.10126
     166 -> 169         0.25686
     166 -> 173        -0.12667
     166 -> 175         0.13331
---------------------------------------------------------------------------------------------------
---------
 Excited state symmetry could not be determined.
 Excited State  15:      Triplet-?Sym    4.2448 eV  292.08 nm  f=0.0000  <S**2>=2.000
     165 -> 173        -0.11128
     166 -> 167         0.11914
     166 -> 169         0.41071
     166 -> 170         0.11212
     166 -> 171         0.23997
     166 -> 173        -0.28700
     166 -> 174        -0.12759
     166 -> 175        -0.19183
---------------------------------------------------------------------------------------------------
---------
```

## References


[S1] Synthesis of compound **1**: $LiN(SiMe_3)_2.Et_2O$ (0.579 g, 2.4 mmol) was added to N-phosphinoamidinato dichlorosilane (1.05 g, 2 mmol) in a reaction flask and cooled to -78 °C, followed by addition of toluene (50 ml). The reaction mixture was allowed to warm to room temperature and stirred overnight. The resulting suspension was filtered, and volatiles were removed. Crude product was extracted with heptane and decanted to obtain yellow solids. For details, see https://hdl.handle.net/10356/170104

[S2] C. Gienger, L. Schynowski, J. Schaefer, C. Schrenk and A. Schnepf, *Eur. J. Inorg. Chem.*, 2023, **26**, e202200738.

[S3] G. M. Sheldrick, SADABS V2014/4 (Bruker AXS Inc.), University of Göttingen, Germany, 2014.

[S4] G. M. Sheldrick, SHELXL-2014/6 (Sheldrick, 2014); Bruker AXS Inc., Madison, WI, USA, 2014.

[S5] Y. Zhao and D. G. Truhlar, *Theor. Chem. Acc.*, 2008, **120**, 215-241.

[S6] F. Weigend and R. Ahlrichs, *Phys. Chem. Chem. Phys.*, 2005, **7**, 3297−3305.

[S7] Gaussian 16, Revision C.01, M. J. Frisch, G. W. Trucks, H. B. Schlegel, G. E. Scuseria, M. A. Robb, J. R. Cheeseman, G. Scalmani, V. Barone, G. A. Petersson, H. Nakatsuji, X. Li, M. Caricato, A. V. Marenich, J. Bloino, B. G. Janesko, R. Gomperts, B. Mennucci, H. P. Hratchian, J. V. Ortiz, A. F. Izmaylov, J. L. Sonnenberg, D. Williams-Young, F. Ding, F. Lipparini, F. Egidi, J. Goings, B. Peng, A. Petrone, T. Henderson, D. Ranasinghe, V. G. Zakrzewski, J. Gao, N. Rega, G. Zheng, W. Liang, M. Hada, M. Ehara, K. Toyota, R. Fukuda, J. Hasegawa, M. Ishida, T. Nakajima, Y. Honda, O. Kitao, H. Nakai, T. Vreven, K. Throssell, J. A. Montgomery, Jr., J. E. Peralta, F. Ogliaro, M. J. Bearpark, J. J. Heyd, E. N. Brothers, K. N. Kudin, V. N. Staroverov, T. A. Keith, R. Kobayashi, J. Normand, K. Raghavachari, A. P. Rendell, J. C. Burant, S. S. Iyengar, J. Tomasi, M. Cossi, J. M. Millam, M. Klene, C. Adamo, R. Cammi, J. W. Ochterski, R. L. Martin, K. Morokuma, O. Farkas, J. B. Foresman, and D. J. Fox, Gaussian, Inc., Wallingford CT, 2016.

[S8] R. E. Stratmann, G. E. Scuseria and M. J. Frisch, *J. Chem. Phys.*, 1998, **109**, 8218-8224.

[S9] E. D. Glendening, C. R. Landis and F. Weinhold, *J. Comput. Chem.*, 2013, **34**, 1429-1437.
